# Intra- and inter-tumor heterogeneity in a vemurafenib-resistant melanoma patient and derived xenografts

**DOI:** 10.15252/emmm.201404914

**Published:** 2015-06-23

**Authors:** Kristel Kemper, Oscar Krijgsman, Paulien Cornelissen-Steijger, Aida Shahrabi, Fleur Weeber, Ji-Ying Song, Thomas Kuilman, Daniel J Vis, Lodewyk F Wessels, Emile E Voest, Ton NM Schumacher, Christian U Blank, David J Adams, John B Haanen, Daniel S Peeper

**Affiliations:** 1 Division of Molecular Oncology, The Netherlands Cancer InstituteAmsterdam, The Netherlands; 2 Division of Experimental Animal Pathology, The Netherlands Cancer InstituteAmsterdam, The Netherlands; 3 Computational Cancer Biology, The Netherlands Cancer InstituteAmsterdam, The Netherlands; 4 Division of Immunology, The Netherlands Cancer InstituteAmsterdam, The Netherlands; 5 Experimental Cancer Genetics, Wellcome Trust Sanger InstituteHinxton, Cambridgeshire, UK

**Keywords:** Melanoma, drug resistance, tumor heterogeneity, patient-derived xenografts

## Abstract

The development of targeted inhibitors, like vemurafenib, has greatly improved the clinical outcome of BRAF^V600E^ metastatic melanoma. However, resistance to such compounds represents a formidable problem. Using whole-exome sequencing and functional analyses, we have investigated the nature and pleiotropy of vemurafenib resistance in a melanoma patient carrying multiple drug-resistant metastases. Resistance was caused by a plethora of mechanisms, all of which reactivated the MAPK pathway. In addition to three independent amplifications and an aberrant form of *BRAF*^*V600E*^, we identified a new activating insertion in *MEK1*. This *MEK1*^*T55delins*^^*RT*^ mutation could be traced back to a fraction of the pre-treatment lesion and not only provided protection against vemurafenib but also promoted local invasion of transplanted melanomas. Analysis of patient-derived xenografts (PDX) from therapy-refractory metastases revealed that multiple resistance mechanisms were present within one metastasis. This heterogeneity, both inter- and intra-tumorally, caused an incomplete capture in the PDX of the resistance mechanisms observed in the patient. In conclusion, vemurafenib resistance in a single patient can be established through distinct events, which may be preexisting. Furthermore, our results indicate that PDX may not harbor the full genetic heterogeneity seen in the patient’s melanoma.

See also: **C Wellbrock** (September 2015)

## Introduction

Until recently, for *BRAF*^*V600E*^ metastatic melanoma, few if any treatments have been available, because it poorly responds to chemotherapy. The development of inhibitors specifically targeting the mutant BRAF^V600E^ protein, such as vemurafenib and dabrafenib, has changed the clinical outcome for patients with this type of tumor. In a randomized phase III clinical trial (BRIM 3), vemurafenib was compared to dacarbazine, the former standard of care. Vemurafenib showed an impressive response rate of 48% compared to 5%, with an increased progression-free survival (5.3 months vs. 1.6 months) (Chapman *et al*, 2011). However, the initial success of the treatment was soon overshadowed by the discovery that almost all patients developed therapy resistance after a period of approximately 6 months (Trunzer *et al*, 2013). The cause of resistance has since then been elaborately studied by many research groups.

This type of research is commonly performed by comparing patient’s samples taken before the start of the targeted treatment with a BRAF inhibitor (pre) and after resistance had occurred (post) (Trunzer *et al*, 2013). Analysis by immunohistochemistry (IHC) revealed that in tumors with acquired resistance, the MAPK pathway was often reactivated (Trunzer *et al*, 2013). Whole-exome sequencing (WES) of matched patient’s samples treated with a BRAF inhibitor (pre and post of a single patient) confirmed these finding (Shi *et al*, 2014b; Van Allen *et al*, 2014). Identified genetic events conferring resistance to BRAF inhibition are activating mutations in *NRAS* (Nazarian *et al*, 2010; Shi *et al*, 2014b; Van Allen *et al*, 2014), *KRAS* (Su *et al*, 2012a; Shi *et al*, 2014b), or *MEK1/2* (Wagle *et al*, 2011; Villanueva *et al*, 2013) or amplification of mutant *BRAF* (Das Thakur *et al*, 2013; Shi *et al*, 2014b; Van Allen *et al*, 2014). Also, alternative splicing of *BRAF*^*V600E*^, by splicing out the RAS-binding domain (RBD) (Poulikakos *et al*, 2011; Van Allen *et al*, 2014), or switching from BRAF to CRAF (Villanueva *et al*, 2010) can be responsible for BRAF inhibitor resistance. A minority of resistant patient’s samples displays reactivation of the AKT/PI3K pathway (but not the MAPK pathway), through activating mutations in *AKT1/3* (Shi *et al*, 2012, 2014a) and *PIK3CA* (Shi *et al*, 2014b; Van Allen *et al*, 2014) or through loss or mutational inactivation of *PTEN*(Shi *et al*, 2014b; Van Allen *et al*, 2014).

As most of the resistance to the single BRAF inhibition is due to reactivation of the MAPK pathway, combining two inhibitors targeting this pathway might be more efficient. Indeed, companion treatment of a specific BRAF inhibitor (dabrafenib) with a MEK inhibitor (trametinib) did increase progression-free survival to an average of 9.4 months (Flaherty *et al*, 2012). Unfortunately, drug resistance continued to limit overall survival. Resistance mechanisms again mainly involved the reactivation of the MAPK pathway, through alternative splicing or amplification of *BRAF*^*V600E*^ or mutation of *MEK2* (Wagle *et al*, 2014).

From the vast amount of different resistance mechanisms identified so far, it is evident that they vary between individual patients and perhaps depend on the genetic background of the treated tumors. Furthermore, reports of different resistance mechanisms within a patient or even in an individual tumor are now emerging (Shi *et al*, 2014b). Here, we present the in-depth analysis of the resistance mechanisms observed in five vemurafenib-resistant metastases of a single patient. In addition, we obtained patient-derived xenografts of these metastases and studied the extent of heterogeneity in resistance mechanisms.

## Results

### Clinical history of the patient

A 34-year-old male, with a family history of melanoma, was diagnosed in December 2011 with ulcerated melanoma with a Breslow thickness of 8 mm. Three of the thirty connecting lymph nodes were affected, but the accompanying CT scan did not reveal any other lesions. The primary tumor and affected lymph nodes were excised, but four months after this surgery, a local recurrence and several new metastases in the connecting lymph nodes and subcutaneous tissue were detected.

**Figure 1:**
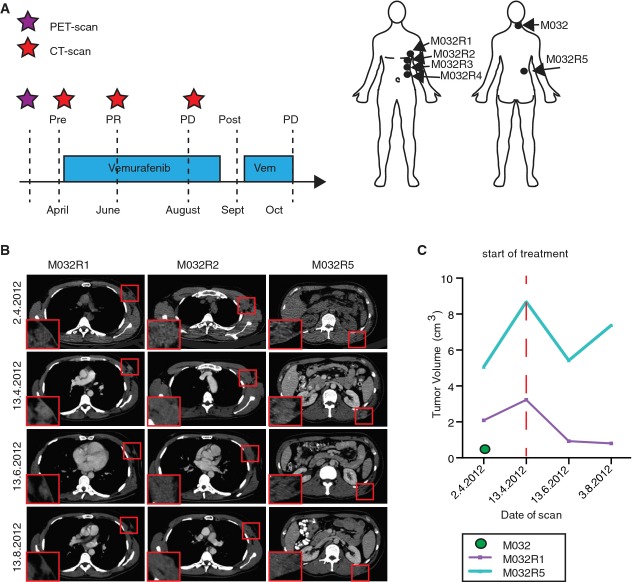
Clinical response of patient M032 to vemurafenib Treatment schedule of patient M032, who received a PET scan two weeks before the start of the treatment (purple asterisk), followed by a baseline CT scan and every two months a follow-up CT scan (red asterisks). Metastases were surgically removed either shortly before the start of the treatment or after resistance occurred (post, M032R1-R5). PR, partial response; PD, progressive disease. Locations of the removed metastases are indicated in the illustration.

The primary tumor was positive for the *BRAF*^*V600E*^ mutation, thereby qualifying the patient for treatment with the BRAF inhibitor vemurafenib. Before the start of the treatment, one subcutaneous metastasis in the neck was excised (referred to as “pre” or M032). In April 2012, the patient started treatment with vemurafenib (960 mg, twice daily) (Fig1A, left panel). The CT scan, taken just prior to the treatment, indicated the presence of several metastases. Initially, a partial response to the treatment was observed, but after four months, the patient progressed on treatment owing to drug resistance. Several progressive metastases were excised after five months of treatment (referred to as “post”). These lesions were derived from distinct locations (armpit left (M032R1), thorax left (M032R2), thorax cranial (M032R3), thorax caudal (M032R4), and back (M032R5) (Fig1A, right panel). After surgery, the patient continued treatment with vemurafenib, but developed recurrent lesions at the surgical excision sites quickly. Therefore, the patient switched to treatment with the anti-CTLA-4 antibody ipilimumab, but deceased shortly thereafter as a result of brain metastases.

Examples of CT scan images of several metastases (M032R1, M032R2, and M032R5).

Volumetric measurements based on the PET and CT scans could be generated from M032, M032R1, and M032R5. Graph illustrates that the metastases R1 and R5 initially expanded before the start of the treatment, but regressed upon vemurafenib treatment. M032R5 showed progressive disease after 4 months, whereas M032R1 still displayed stable disease. M032 was excised and no recurrence was observed.

The individual response to vemurafenib of the obtained metastases was analyzed by performing volumetric tumor measurements on PET and CT scans (Fig1B and C), which could be done for two resistant metastases (M032R1 and M032R5). The pre-treatment tumor was excised before the start of the treatment (and before the baseline CT scan) and did not recur (Fig1C). The other resistant metastases could not be measured due to diffuse borders (M032R2) or to uncertainty of the tumor location on CT scan (M032R3, M032R4). For M032R1 and M032R5, the initial growth of the metastases was analyzed by comparing the PET scan, taken two weeks before the start of the treatment, to the baseline CT scan. M032R5 displayed initial rapid growth, but regressed after the start of the treatment. However, after four months, M032R5 increased in volume again. In contrast, M032R1 regressed upon vemurafenib treatment and displayed continuous inhibition of outgrowth, suggesting stable disease of this specific lesion (Fig1C). Although the response of each of the individual metastases was different, the overall response of the patient, according to RECIST criteria (version 1.1), qualified as a partial response after two months and progressive disease after four months of treatment (Fig1A).

### Vemurafenib-resistant metastases display reactivation of MAPK signaling

**Figure 2:**
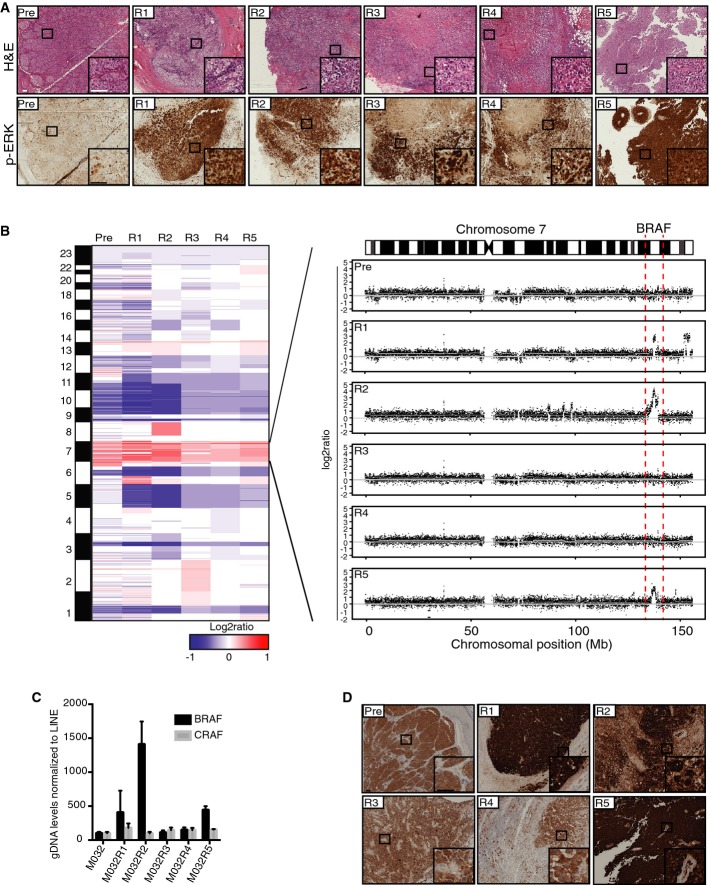
Reactivation of MAPK pathway via amplification of a BRAF-encoding DNA fragment caused resistance to vemurafenib in three metastases Hematoxylin–eosin (H&E) and p-ERK stainings on FFPE material of all metastases showed that all vemurafenib-resistant tumors had reactivation of the MAPK pathway. Scale bar represents 100 μm.

As ERK reactivation is commonly seen in vemurafenib resistance (Shi *et al*, 2014b; Van Allen *et al*, 2014), we first analyzed the activation of BRAF downstream in the MAPK pathway. Therefore, formalin-fixed, paraffin-embedded (FFPE) archival tissue of each of these metastases was stained for phosphorylated ERK (p-ERK) (Fig2A). The pre-treatment tumor displayed a relatively low-level phosphorylation of ERK, but higher than seen in control skin, in agreement with the observation that this concerned a BRAF mutant melanoma (Supplementary Fig S1). All five vemurafenib-resistant metastases showed highly upregulated p-ERK levels compared to the pre-treatment lesion, suggesting that resistance was caused by reactivation of the MAPK pathway.

CNA profiles were generated from the WES data with germ line DNA as a reference. Colors represent segmented log2 ratio values with red for gain and blue for loss. Further inspection of chromosome 7 where *BRAF* is located revealed amplification (7q34) of this region in three vemurafenib-resistant metastases, namely M032R1, M032R2, and M032R5.

qPCR was performed on gDNA retrieved from each of the metastases, using primers for *BRAF* and *CRAF* and normalized on *LINE* levels. Bars represent the mean of three replicates, error bars indicate standard deviation. The results confirmed that *BRAF* was amplified in M032R1, M032R2, and M032R5.

Staining for BRAF^V600E^ with a mutant epitope-specific antibody confirmed the upregulation of BRAF^V600E^ in R1, R2, and R5. Scale bar represents 100 μm.

### Copy number aberration profiling reveals BRAF amplification in three resistant metastases

To study the mechanism of resistance in the metastases, we performed WES on all samples. To this end, DNA was isolated from all snap-frozen tumor specimens and from peripheral blood, as a germ line DNA reference. Next, the DNA samples were analyzed by WES with an average coverage of ∼40× (details in Supplementary Table S1). This revealed an *E318K* germ line mutation in *MITF*, which has been associated with increased melanoma susceptibility (Bertolotto *et al*, 2011; Yokoyama *et al*, 2011). DNA copy number profiles were generated from the WES data using a new tool, “CopywriteR,” which we recently reported (Kuilman *et al*, 2015). The derived copy number aberration (CNA) plots indicated that all metastases likely had a common ancestor, since most of the gains and losses were shared between the pre- and the post-metastases (Fig2B, left panel). In addition, in three of the five resistant metastases (M032R1, M032R2, M032R5), an amplification of the genomic region harboring the *BRAF* locus was observed (Fig2B, right panel), which has been previously identified as a prevalent vemurafenib resistance mechanism (Shi *et al*, 2012; Das Thakur *et al*, 2013; Villanueva *et al*, 2013). Remarkably, each of these tumors harbored differently sized amplicons, suggesting that they had occurred independently, rather than originated from a single parental tumor that already harbored this amplification (Supplementary Fig S2).

To validate the *BRAF* amplification, a quantitative PCR (qPCR) was performed on gDNA derived from the different metastases. This analysis confirmed the presence of the amplification of *BRAF* in M032R1, M032R2, and M032R5 (Fig2C). Staining with a specific BRAF^V600E^ antibody on FFPE archival tissue confirmed the upregulation of BRAF^V600E^ in these lesions (Fig2D). These stainings also revealed that the expression of BRAF^V600E^ is highly heterogeneous; this will be discussed below.

### Aberrant form of BRAF

**Figure 3:**
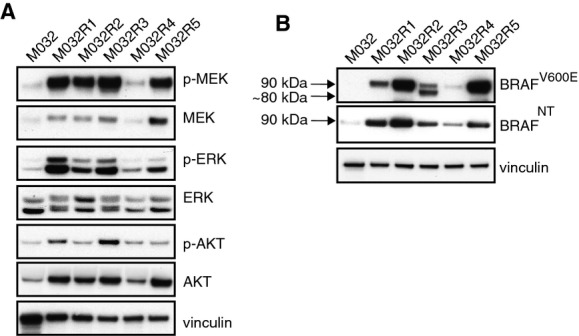
Alternative form of BRAF^V600E^ is correlated with p-ERK and p-MEK reactivation in M032R3 Immunoblotting for MAPK pathway components confirmed reactivation of p-ERK and p-MEK in all resistant metastases.

For further analysis of the mechanisms of vemurafenib resistance in these metastases, protein was extracted from snap-frozen tumor pieces of all the metastases and an immunoblot was performed to determine the activity of the MAPK and AKT/PI3K pathways (Fig3A). Most resistant metastases showed reactivation of the MAPK pathway, determined by increased expression levels of both p-ERK and p-MEK, although some variation was observed among different metastases. Immunoblotting for the mutant BRAF^V600E^ protein revealed, besides higher expression of BRAF^V600E^ in M032R1, M032R2, and M032R5, an aberrant form of BRAF in M032R3. The full length of BRAF^V600E^ is 90 kDa, while this aberrant form had an apparent molecular weight of ∼80 kDa (Fig3B). Previously, it has been shown that alternative splicing of *BRAF*^*V600E*^, which removes the Ras-binding domain (RBD), induces resistance to BRAF inhibition (Poulikakos *et al*, 2011). However, we could not identify an alternatively spliced product in M032R3. Alternatively, this aberrant protein could be a fusion product comprising the C-terminus of BRAF and the N-terminus of an unknown protein. Fusion between *BRAF* and other genes has previously been shown to hyperactivate the MAPK pathway (Ciampi *et al*, 2005; Cin *et al*, 2011; Botton *et al*, 2013) and induce resistance to BRAF inhibition (Botton *et al*, 2013). Therefore, we performed an immunoblot on the M032 protein lysates, using an antibody directed against the N-terminus of the BRAF protein. This blot showed that the 80-kDa aberrant form of BRAF, which could be detected by BRAF^V600E^ antibody, could not be identified with the antibody recognizing the N-terminus of BRAF (Fig3B), suggesting that this protein is encoded by a fusion gene. Although it is conceivable that a gene fusion is responsible for the expression of this aberrant BRAF protein driving vemurafenib resistance, due to poor RNA quality of this metastasis, we were unable to investigate this further.

Immunoblotting for M032 patient’s samples using an antibody directed against either the mutant BRAF^V600E^ epitope or an N-terminal region of BRAF revealed the expression of a shorter variant of BRAF^V600E^ in M032R3, of approximately 80 kDa. This 80-kDa band could not be detected with the N-terminus-recognizing antibody. Source data are available online for this figure.

### A new 3-base pair in-frame insertion in *MEK1* induces resistance to vemurafenib *in vitro*

**Figure 4:**
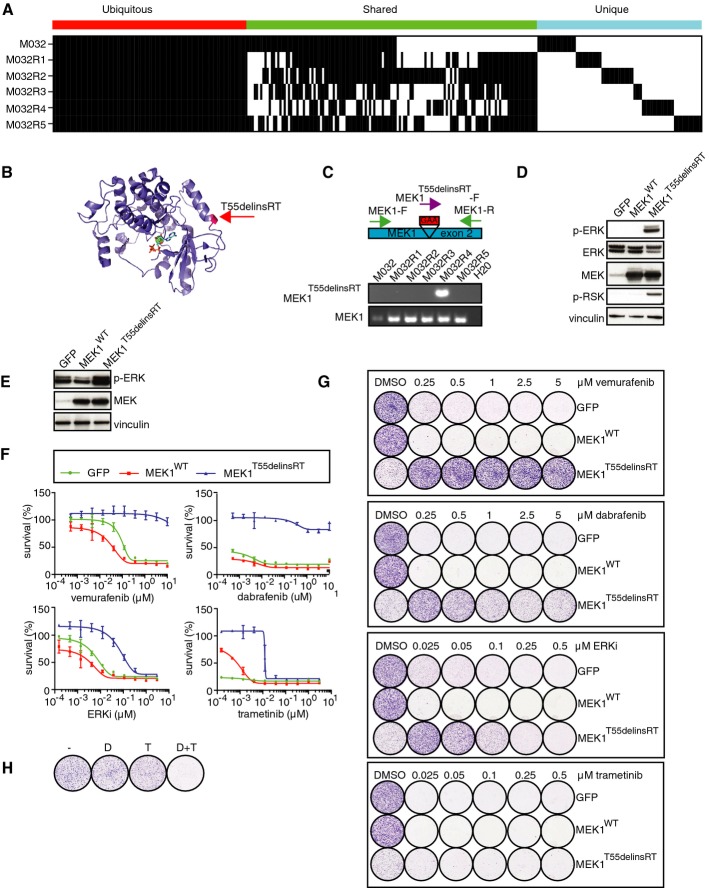
*T55delinsRT* mutation in *MEK1* is responsible for resistance to vemurafenib in M032R4 Somatic mutations present in each of these metastases, revealing that mutations were either shared by the pre-treatment tumor and all metastases, by some metastases, or only by single metastases.

After discovering *BRAF* amplifications and an aberrant form of BRAF in four of the five resistant metastases, which are likely responsible for the vemurafenib resistance, the resistance observed in one metastasis (M032R4) was still unexplained. To elucidate this, we retrieved somatic mutations from the WES data obtained from the metastases. A mutation matrix was generated, distributing the mutations into three groups: (i) mutations that were shared between all metastases (i.e., ubiquitous); (ii) mutations that were shared between two or more metastases (i.e., shared), and (iii) mutations that were present in only one metastasis (i.e., private) (Fig4A**,** Supplementary Table S2).

A 3-bp in-frame insertion in *MEK1* (*T55delinsRT*) was identified in M032R4. This mutation was located at the base of the α-helix at the N-terminal site of MEK1.

Validation of *T55delinsRT* was done by PCR using insertion-specific primers. As a control, primers amplifying exon 2 of MEK1^WT^ were used.

Transfection of pQXCIP-GFP, MEK1-WT, or MEK1-T55delinsRT into HEK293T cells showed that this mutation induces p-ERK and p-RSK.

Validation of expression of pQXCIP-GFP, MEK1^WT^, or MEK1^T55delinsRT^ in A375 melanoma cells by immunoblotting.

Dose–response curves for A375 melanoma cells expressing GFP, MEK1^WT^, or MEK1^T55delinsRT^ with indicated doses of BRAF inhibitor vemurafenib, ERK inhibitor SCH772984, or MEK inhibitor trametinib. Error bars indicate standard deviation.

Colony formation assays with A375 melanoma cells, infected with GFP, MEK1^WT^, or MEK1^T55delinsRT^-encoding lentivirus, and treated with indicated doses and inhibitors.

Treatment of A375 *MEK1*^*T55delinsRT*^ melanoma cells with DMSO (−), 250 nM dabrafenib (D), 10 nM trametinib (T), or a combination (D+T). Source data are available online for this figure.

We identified in M032R4 a unique 3-base pair in-frame insertion in exon 2 of the *MEK1* gene (*MEK1*^*T55delinsRT*^), potentially explaining the resistance observed in this metastasis. Modeling of this mutation revealed that its location was at the base of helix A at the N-terminal part of MEK1 (Fig4B). This region has been previously shown to be important for repression of MEK1 kinase activity (Fischmann *et al*, 2009). Furthermore, mutations that affect the conformation of helix A (e.g., *E203K*) can trigger constitutive intrinsic activation of *MEK1* (Nikolaev *et al*, 2012). PCR analysis of the gDNA of M032R4 using a primer annealing at the insertion site confirmed the *MEK1*^*T55delinsRT*^ insertion. The *MEK1*^*T55delinsRT*^ mutation was uncovered specifically in M032R4 and was not detected in any of the other resistant tumors nor the pre-treatment tumor (Fig4C).

To determine any functional consequences of this mutation, we cloned *MEK1*^*T55delinsRT*^ into an expression plasmid, which was transiently transfected into HEK293T cells. Immunoblotting revealed that the *MEK1*^*T55delinsRT*^ induced hyperphosphorylation of ERK, when compared to the *GFP* control and *MEK1*^*WT*^ (Fig4D). Next, we retrovirally transduced *BRAF*^*V600E*^ melanoma cell lines with either *GFP*, *MEK1*^*WT*^, or *MEK1*^*T55delinsRT*^ and analyzed their growth behavior in the presence of several MAPK pathway inhibitors by dose–response curves and colony formation assays (Fig4E–G**,** Supplementary Figs S3 and S4). Also in this setting, expression of MEK1^T55delinsRT^ caused upregulation of p-ERK when compared to GFP or MEK1^WT^ control (Fig4E, Supplementary Figs S3 and S4). Melanoma cells carrying *MEK1*^*T55delinsRT*^ displayed resistance to vemurafenib as well as to dabrafenib, in contrast to GFP- and MEK1^WT^-expressing cells. MEK1^T55delinsRT^-expressing cells were also more resistant to low levels of the ERK inhibitor SCH772984 than control populations (Fig4F and G, Supplementary Figs S3 and S4). Interestingly, melanoma cells carrying MEK1^T55delinsRT^ actually proliferated faster when treated with low levels of BRAF or ERK inhibitor, when compared to control treatment (Fig4G, Supplementary Figs S3 and S4). Previously, it has been shown that hyperactivation of the MAPK pathway can diminish cell proliferation and survival (Sun *et al*, 2014; Moriceau *et al*, 2015). Upon treatment with MAPK pathway inhibitors, this cytostatic activity could be rescued (Sun *et al*, 2014; Moriceau *et al*, 2015). Consistent with those data, we found by immunoblotting and cell proliferation assays that in MEK1^T55delinsRT^-expressing melanoma cells, normalization of p-ERK levels by moderate BRAF or ERK inhibitor treatment rescued the cytostatic activity of hyperactivated MAPK signaling (Fig4G, Supplementary Figs S3–S5).

On the other hand, relatively low doses of the MEK inhibitor trametinib were sufficient to kill *MEK1*^*T55delinsRT*^ cells, although these cells were less sensitive than control cells (Fig4F and G, Supplementary Figs S3 and S4). This was in contrast to observations for other MEK inhibitors, like PD-0325901, U0126, and selumetinib (AZD6244), which also function as non-ATP-competitive inhibitors. Those MEK inhibitors were less potent to kill melanoma cells carrying the MEK1^T55delinsRT^ mutation (Supplementary Fig S6), which could be caused by less potency of the drug (see Discussion).

Combination therapy of dabrafenib and trametinib, currently used in the clinic, also eradicated all melanoma cells with MEK1^T55delinsRT^ (Fig4H**,** Supplementary Figs S3 and S4). Together, these data demonstrate that this new 3-base pair insertion in MEK1 confers resistance to several MAPK pathway inhibitors *in vitro*, yet can be inhibited relatively effectively by trametinib alone but more profoundly by a combination of dabrafenib and trametinib.

### The *MEK1*^*T55delinsRT*^ mutation induces resistance to dabrafenib and promotes local invasion *in vivo*

**Figure 5:**
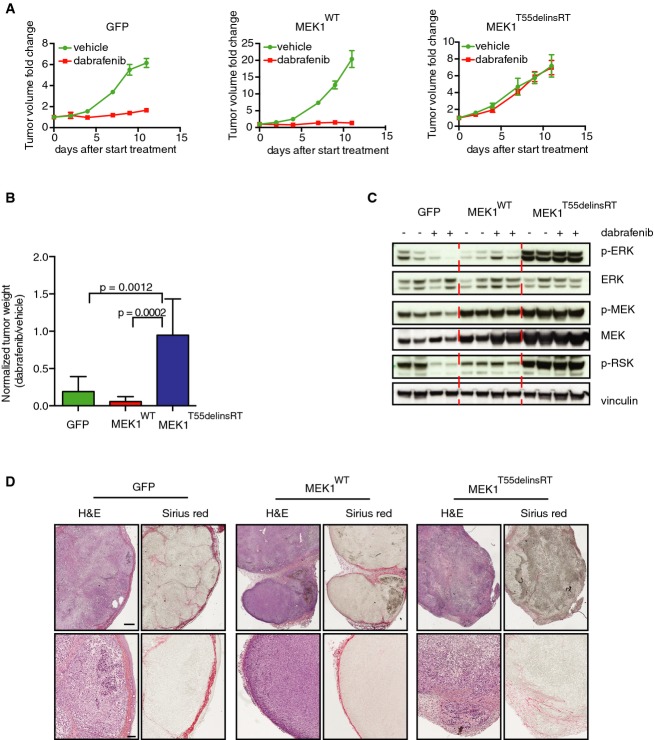
*T55delinsRT* mutation in *MEK1* confers resistance to BRAF inhibition and promotes local invasion *in vivo* A375 melanoma cells expressing GFP, MEK1^WT^, or MEK1^T55delinsRT^ were injected into immune-deficient mice (*n* = 8 per group), and after the tumor size of ˜100 mm^3^ was reached, mice were treated with 30 mg/kg dabrafenib or vehicle. Graphs represent fold change in tumor volume normalized on the tumor volume on the day of the start of the treatment. Error bars indicate standard error of the mean.

In order to determine whether the *MEK1*^*T55delinsRT*^ mutation conferred resistance to BRAF inhibition *in vivo* also, A375 melanoma cells*,* after they had been retrovirally transduced with cDNAs encoding *GFP*, *MEK1*^*WT*^ or *MEK1*^*T55delinsRT*^, were injected subcutaneously into immune-deficient mice. After the tumors had reached ∼100 mm^3^ in size, mice were treated with vehicle or 30 mg/kg dabrafenib six days per week. Treatment with dabrafenib inhibited tumor growth in *GFP* and *MEK1*^*WT*^ tumors, but did not affect the growth of the *MEK1*^*T55delinsRT*^ tumors (Fig5A and B). Analyses of the MAPK signaling pathway by immunoblotting confirmed reduction of p-ERK and p-RSK in *GFP* and *MEK1*^*WT*^ tumors, but not in the *MEK1*^*T55delinsRT*^ tumors. In the latter tumors, phosphorylation of ERK and RSK was already at a higher baseline level compared to the *GFP* and *MEK1*^*WT*^ tumors and this could not be inhibited by the drug (Fig5C).

Average tumor weight of all groups at the end of the experiment described in (A). Error bars indicate standard deviation. Unpaired *t*-test was used to calculate statistical significance. *P*-values are indicated in the graph.

Immunoblotting for MAPK pathway components performed on tumors of all groups.

Immunohistochemistry for Sirius Red (staining for collagen) and H&E on tumors from all groups, to identify grade of demarcation and local invasion. Scale bar represents 500 (upper row) and 100 (lower row) μm. Source data are available online for this figure.

Next, we analyzed the xenografts and discovered a striking difference between the control GFP and MEK^WT^ tumors and the *MEK1*^*T55delinsRT*^ tumors: in the control tumors, the demarcations were well defined by the stromal tissue of mainly collagen (stained by Sirius red histochemistry, Fig5D), whereas the *MEK1*^*T55delinsRT*^ tumors were incompletely demarcated by surrounding stromal tissue (Fig5D). These data show that tumors harboring the *MEK1*^*T55delinsRT*^ mutation have increased local invasion and therefore likely have a higher propensity to spread to distant organs. Altogether, these data confirm that *MEK1*^*T55delinsRT*^ confers resistance to BRAF inhibition *in vivo* and induces local invasion.

### *MEK1*^*T55delinsRT*^ is a preexisting mutation

**Figure 6:**
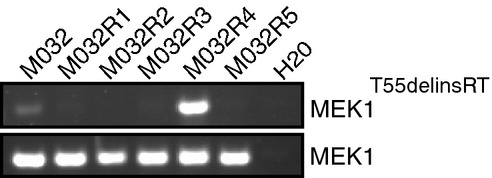
*MEK1*^*T55delinsRT*^ is a preexisting mutation gDNA was isolated from another fragment of the tumor resection, and the PCR with the insertion-specific primers was repeated. Primers amplifying exon 2 of *MEK1*^*WT*^ were used as an input control. Source data are available online for this figure.

It has been suggested previously that resistance-conferring mutations may exist in the tumor prior to therapy (Gerlinger & Swanton, 2010; Diaz *et al*, 2012). However, robust in-depth DNA sequencing analysis is required to detect these low-frequency mutated clones. Therefore, we wondered whether we had inadvertently missed the *MEK1*^*T55delinsRT*^ mutation in the first analysis of the pre-treatment tumor M032. We isolated gDNA from a new sample of snap-frozen biopsy fragments derived from the same pre- and post-vemurafenib metastases of our first analysis and repeated the insertion-specific PCR. In these independent samples, we not only confirmed the *MEK1*^*T55delinsRT*^ insertion in M032R4, but now also identified the insertion in the pre-treatment sample (Fig6). This result indicates that the *MEK1*^*T55delinsRT*^ insertion was preexisting in a subset of the pre-treatment tumor, thereby underscoring the extensive heterogeneity of these tumors.

### Incomplete capture of tumor heterogeneity in patient-derived xenografts

In parallel to the aforementioned analyses, we transplanted fragments of the pre-treatment and all resistant metastases subcutaneously into immune-deficient *NOD-scid IL2Rgamma*^*null*^ mice. Once these patient-derived xenografts (PDX; referred to as .X1) had grafted (without BRAF inhibitor treatment), mice were sacrificed and tumor pieces were either fixed and archived as FFPE material or snap-frozen and stored at −80**°**C for further analyses. Except for M032R3, all the metastases could be grafted successfully.

**Figure 7:**
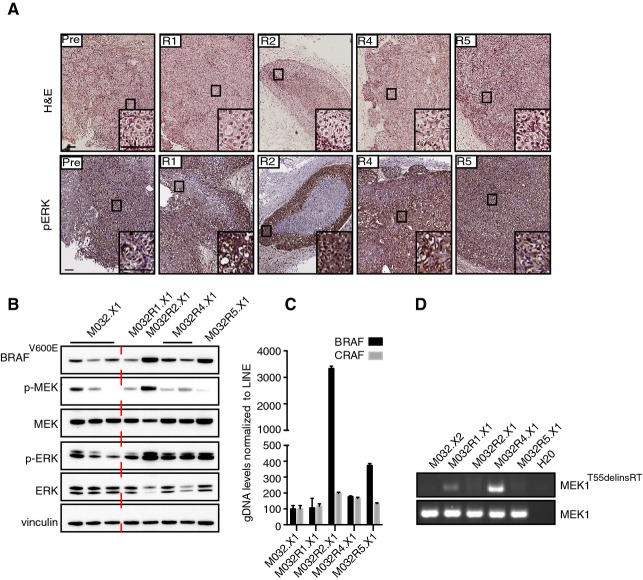
Patient-derived xenografts display similar resistance mechanisms as the parental metastases Staining for hematoxylin–eosin (H&E) and p-ERK on the PDX samples M032.X1, M032R1.X1, M032R2.X1, M032R4.X1, and M032R5.X1. Stainings showed that p-ERK is higher in the PDX derived from the resistant metastases. Scale bar represents 100 μm.

We next determined whether the resistance mechanisms in the patient’s tumors were captured in the PDX. First, FFPE slides from PDX were stained for p-ERK. We found that p-ERK was expressed to higher levels in the PDX derived from the resistant metastases compared to one PDX derived from the pre-treatment sample (Fig7A), comparable to what was seen in the patient’s samples. Next, we performed immunoblotting on the PDX samples (Fig7B). This revealed BRAF^V600E^ protein elevation in M032R2.X1 and M032R5.X1, but not in M032R1.X1 (Fig7B), which was confirmed by qPCR for *BRAF* performed on gDNA samples derived from all M032 PDX samples (Fig7C). Immunoblotting for p-MEK and p-ERK revealed heterogeneity of the MAPK pathway signaling activity, which was already present in the pre-treatment PDX (Fig7B).

Immunoblotting for components of the MAPK pathway confirmed reactivation of p-ERK in the PDX derived from the vemurafenib-resistant metastases, although the p-ERK signal is heterogeneous in the pre-treatment PDX.

Validation of the BRAF amplification was performed by qPCR on gDNA derived from the PDX, using primers for BRAF and CRAF and normalized on LINE expression. Bars represent the mean of three replicates, error bars indicate standard deviation. The results confirmed that BRAF was amplified in PDX derived from M032R2 and M032R5, but not from M032R1.

Presence of the *MEK1*^*T55delinsRT*^ was analyzed by PCR using insertion-specific primers. As a control, primers amplifying exon 2 of *MEK1*^*WT*^ were used. The insertion was found in M032R1.X1 and M032R4.X1. Source data are available online for this figure.

As the presence of the *BRAF* amplification was incompletely overlapping between patient and PDX samples, we decided to study BRAF^V600E^ expression in these tumors in more detail by IHC. We observed that the expression of BRAF^V600E^ was highly heterogeneous within both the parental tumors (Supplementary Fig S7A) and the PDX (Supplementary Fig S7B). For instance, the patient’s tumor M032R1 had regions of both high and low BRAF^V600E^ expression **(**Supplementary Fig S7A). BRAF^V600E^ was found to be overexpressed in M032R1, which was confirmed by qPCR performed on gDNA (Fig2B and C). Remarkably, repeating this experiment on gDNA derived from another set of tumor fragments derived from the same excisions failed to show amplification of *BRAF* in M032R1 (Supplementary Fig S7C). Conceivably, this inconsistency is explained by the heterogeneity of the *BRAF* amplification in this patient’s sample, and the concomitant heterogeneity of BRAF^V600E^ expression in the PDX of M032R1. Of note, this specific lesion displayed disease stabilization rather than progressive disease at the moment of excision, raising the possibility that selection for the resistant clone was still ongoing.

**Figure 8:**
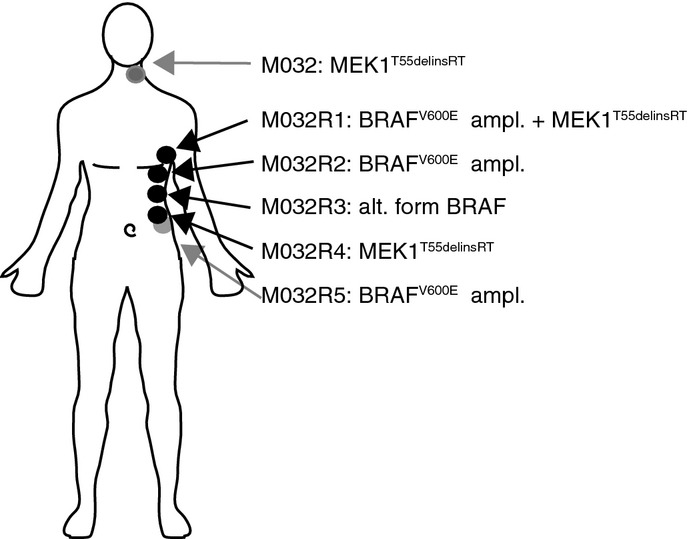
Overview of all identified vemurafenib resistance mechanisms in patient and PDX samples Resistance mechanisms discovered in each of the lesions have been identified: a) in patients samples by WES (*BRAF*^*V600E*^ amplification in M032R1, R2 and R5; *MEK1*^*T55delinsRT*^ in M032R4 and M032) and immunoblotting (of alternative form of BRAF in M032R3); and b) in PDX by IHC and qPCR (*BRAF*^*V600E*^ amplification in M032R2 and R5) and PCR analysis (*MEK1*^*T55delinsRT*^ in M032R1 and R4). Gray arrows indicate that the lesion was at the back side. ampl., amplification; alt., alternative.

The presence of the *MEK1*^*T55delinsRT*^ mutation was analyzed by PCR with the primers specific for the insertion, which confirmed the presence of this mutation in M032R4.X1 (Fig7D). Remarkably, *MEK1*^*T55delinsRT*^ was detected also in PDX M032R1.X1 (Fig7D), suggesting that the original M032R1 tumor harbored, in addition to the *BRAF*^*V600E*^ amplification, other resistance mechanisms, including the *MEK1*^*T55delinsRT*^ mutation. The wide variety of resistance mechanisms identified in both patient and PDX samples has been summarized in Fig8.

From these observations, we conclude that PDX samples can be used to model the patient’s tumors, because they harbor the same resistance mechanisms. However, our results also clearly indicate that it can be challenging to capture the full extent of genetic heterogeneity seen in the original tumor in the PDX setting.

## Discussion

Resistance to targeted therapy, like the BRAF inhibitor vemurafenib, creates a formidable challenge to overcome or prevent. To increase our understanding of its mechanistic basis, and to explore to what extent the heterogeneity of drug resistance can be captured in PDX, we have studied the variation and extent of vemurafenib resistance mechanisms present in a melanoma patient and xenografts derived thereof (Fig8). We have sequenced five resistant metastases and a pre-treatment sample derived from the same *BRAF*^*V600E*^ melanoma patient. We identified an amplification of *BRAF*^*V600E*^ in three resistant metastases (M032R1, M032R2, and M032R5), which likely occurred as independent events. A fourth drug-resistant metastasis harbored an aberrant form of BRAF (M032R3), whereas in yet another metastasis, a new activating in-frame insertion in MEK1 (*MEK1*^*T55delinsRT*^; M032R4) was identified. Validation and characterization *in vitro* and in mice confirmed that this MEK mutant caused vemurafenib resistance. Furthermore, it drove local invasion of melanoma cells.

All resistance mechanisms induced reactivation of the MAPK pathway, but our results suggest that these events occurred independently. Furthermore, the resistance-conferring BRAF amplification was heterogeneous even within a single metastasis, suggesting that multiple resistance mechanisms can be present within a single tumor. Since we sequenced DNA derived from small fragments from these metastases, we may have underestimated the number of vemurafenib-resistant subclones.

The hypothesis of heterogeneous resistance mechanisms is further supported by the findings in our PDX, which we derived from all but one of the resistant metastases. In these PDX, we have studied the presence of the resistance mechanisms identified in the corresponding patient’s biopsies. Importantly, the identified resistance mechanisms in the PDX incompletely shared those uncovered in the samples taken from the patient. For instance, the *BRAF* amplification, whose presence was already heterogeneous in M032R1, was not detected in the corresponding PDX. This could simply be explained by assuming that the particular tumor fragment from which this PDX was derived did not contain the *BRAF* amplification. Strikingly, this PDX did contain the *MEK1*^*T55delinsRT*^ mutation we identified in M032R4. Again, this indicates that the parental tumor harbored multiple resistance mechanisms in distinct subclones. This finding illustrates an important point: as is common for PDX, the melanoma PDX we grew were derived from small tumor fragments, which originated from sometimes large and (highly) heterogeneous lesions. Although this does not disqualify the use of PDX as proxies for studying tumorigenesis and therapy response, it does warrant a note of caution: given the enormous extent of heterogeneity (in melanoma, but in several other tumor types as well for that matter), one cannot safely assume that the PDX data can be projected 1:1 on the behavior of the tumors in patients.

We also describe the identification and validation of a new vemurafenib-resistance-conferring mutation: an in-frame insertion in *MEK1* (*MEK1*^*T55delinsRT*^). Based on its location (Nikolaev *et al*, 2012), the mutation probably causes a conformational change in MEK1, thereby constitutively phosphorylating ERK and activating downstream MAPK signaling. As a consequence, this mutation induced resistance to BRAF and ERK inhibitors alike, but not to the MEK inhibitor trametinib. Other MEK inhibitors, however, like U0126, PD-0325901, and selumetinib, could not eliminate melanoma cells carrying *MEK1*^*T55delinsR*T^ (Supplementary Fig S6), despite the fact that these MEK inhibitors share the mode of action with trametinib: They all are non-competitive with ATP inhibitors, binding MEK1 adjacent to the ATP-binding site (Gilmartin *et al*, 2011). The main difference between trametinib and the other three MEK inhibitors therefore seemed to be the potency of inhibition, at least in this setting.

Other mutations in *MEK1* and *MEK2* conferring resistance to MAPK pathway inhibition have been identified previously. The presence of *MEK1/2* mutations prior to the start of BRAF inhibitor treatment may reduce the therapeutic response in the patient and may therefore act as a prognostic factor. Indeed, it has been suggested that the presence of preexisting *MEK1*^*P124*^ mutations is associated with poor response and shorter progression-free survival (PFS) in melanoma patients treated with BRAF inhibitor (Carlino *et al*, 2015). Together, these data suggest that the decision for therapy should not only be based on the presence of *BRAF*^*V600E*^ mutation, but also preexisting *MEK1/2* mutations should be analyzed in order to guide better therapy choices.

The *MEK1*^*T55delinsRT*^ mutation can be targeted by combination therapy of dabrafenib and trametinib (Fig4H, Supplementary Figs S3 and S4). Recently, companion treatment of a BRAF inhibitor and a MEK inhibitor has been shown to delay the onset of acquired resistance and thereby increase progression-free survival (Larkin *et al*, 2014; Long *et al*, 2014). Unfortunately, resistance continues to occur. A previously identified resistance mechanism to the combination treatment of MEK and BRAF inhibition is amplification of *BRAF* (Wagle *et al*, 2014). This resistance mechanism was also detected in three out of five vemurafenib-resistant metastases of the patient in this study. Therefore, our data suggest that some drug-resistant clones would have responded from targeted inhibition of one or multiple factors of the MAPK pathway, and the patient would have benefitted only little from combination therapies with two targeted BRAF pathway inhibitors due to the number and variety of resistance mechanisms. Conceivably, this represents a common problem that predicts that only cocktails of targeted and/or immune inhibitors will be able to increase patient survival.

Our analyses also showed that the *MEK1*^*T55delinsRT*^ mutation was already present in a fraction of the melanoma prior to the treatment (Fig6). The presence of resistance-conferring mutations or aberrations in a small subpopulation of tumor cells in pre-treatment lesions has been previously suggested to be the cause of resistance (Gerlinger & Swanton, 2010; Diaz *et al*, 2012), as targeted therapy itself is likely not mutagenic. These subclones apparently have a selective proliferative advantage when the patient is on treatment with a specific inhibitor, like vemurafenib, resulting in its clonal expansion. This is remarkable, since there are no data to suggest that in the absence of treatment, constitutive MEK activity can act as a classical driver mutation. Subclones carrying a resistance-inducing mutation are detected only sporadically, probably due to insufficient sequence depth combined with the heterogeneity of melanomas.

The existence of intrinsically resistant subclones before the start of the treatment has been recognized previously in other tumor types. For example, in non-small cell lung cancer (NSCLC), a gefitinib resistance-conferring mutation in *EGFR* (*T790M*) has been identified in the pre-treatment tumor or circulating tumor cells (Inukai *et al*, 2006; Maheswaran *et al*, 2008; Su *et al*, 2012b). Also, mutations in the ABL domain, which confer resistance to imatinib, can be detected in rare subpopulations of *BCR-ABL*-positive chronic myeloid leukemia (CML) (Shah *et al*, 2002). For melanoma, the existence of a rare population of cells in pre-treatment lesions, carrying mutations that induce resistance to targeted therapy, has, to our knowledge, not been described previously.

In conclusion, we have shown here that the cause of resistance against vemurafenib can be strikingly different within distinct metastases of a single patient and even within single lesions. We also describe a new resistance-conferring mutation in *MEK1*, namely *MEK1*^*T55delinsRT*^, and show that this mutation was preexisting in the pre-treatment sample. Finally, we show that PDX, derived from the therapy-resistant metastases, can display similar pleiotropy in resistance mechanisms, making PDX a valuable experimental platform for studying drug responses. However, our data clearly demonstrate that (spatial) heterogeneity of the parental tumor makes it challenging to capture the full extent of it in the context of PDX.

## Materials and Methods

### Patient’s sample

The Medical Ethical Board of the Antoni van Leeuwenhoek has approved the collection and use of human tissue. The patient in this study signed an informed consent for this study. As a germ line control, blood was retrieved from the patient. The tumors were excised by surgery, either before the start of the treatment (pre) or after the patient had become resistant (M032R1-R5), and directly snap-frozen or fixed in formalin and embedded in paraffin for later use.

### Volumetric measurements of metastases

A radiologist was consulted to identify all tumor lesions that were surgically resected on pre-, on-, and post-treatment CT scans. Volumetric assessment was only performed on tumor lesions that displayed clear margins on CT. Three-dimensional (3D) volumetric measurements were performed in the transversal plane, using the semi-automatic software tool EncoreUnFoie v5.0 (Image Sciences Institute, Utrecht, the Netherlands).

### Animals and Patient-Derived Xenografts

Animal experiments were approved by the animal experimental committee of the institute and performed according to Dutch law. PDX were generated in *NOD.Cg-Prkdc*^*scid*^ *Il2rg*^*tm1Wjl*^*/SzJ* mice (male and female, ∼6 weeks) by subcutaneous transplantation of ∼5 mm^3^ melanoma fragments. Mice were kept under IVC conditions. Tumor outgrowth was measured twice per week with a caliper. Before reaching the maximum allowed tumor size of 1000 mm^3^, mice were sacrificed and tumors were removed and either fixed in formalin and embedded in paraffin (FFPE) or snap-frozen and stored at −80°C for further analyses. The *in vivo* experiment was performed with A375 cells infected with pQXCIP-GFP, MEK1^WT^, and MEK1^T55delinsRT^. A total number of 24 mice, resulting in 4 mice per experimental group (male, age 5–7 weeks, 2 flanks per mouse, so *n* = 8 tumors/group) were injected with 250,000 tumor cells per flank. After the tumors reached 100 mm^3^, mice were randomized based on age and cage and daily treatment (6 days per week) by oral gavage was started with control vehicle or dabrafenib (M1988, Abmole). Dabrafenib powder was first dissolved in DMSO and consequently, before injection, dissolved in 0.5% hydroxypropylmethylcellulose (Sigma-Aldrich), 0.2% Tween-80 in pH 8.0 distilled H_2_O. Tumor outgrowth was measured twice per week with a caliper. Before reaching the maximum allowed tumor size of 1,000 mm^3^, mice were sacrificed and tumors were removed and either fixed in formalin and embedded in paraffin (FFPE) or snap-frozen and stored at −80°C for further analyses.

### Staining on patient, PDX, and xenograft samples

Stainings were performed on FFPE archival patient tumor material by our in-house NKI-AVL Core Facility Molecular Pathology & Biobanking (CFMPB) for hematoxylin–eosin, rabbit anti-p-ERK (4370, Cell Signaling), and mouse anti-BRAF^V600E^ (VE1, Spring Bioscience). Xenografted tumors were stained by our in-house Animal Pathology Facility for Sirius Red and hematoxylin–eosin.

### Immunoblotting

Immunoblotting was performed as described previously (Possik *et al*, 2014). The following antibodies were used: mouse anti-p-ERK1/2 (E10, #9106), rabbit anti-ERK1/2 (9102), rabbit anti-p-MEK (41G9, #9154), mouse anti-MEK (L38C12, #4649) from Cell Signaling, mouse anti-BRAF^V600E^ (VE1, Spring Bioscience), rabbit anti-p-RSK (04-419, Millipore), rabbit anti-RSK (9355, Cell Signaling), mouse anti-B-RAF (F7, Santa Cruz), mouse anti-vinculin (V9131, Sigma), and rabbit anti-p-AKT (D9E, #4060, Cell Signaling). Densitometry measurements were analyzed using ImageJ 1.48R software. Band intensities were calculated as relative to control bands (DMSO control of MEK^WT^) and adjusted to loading control band values.

### DNA isolation and sequencing

DNA was isolated from granulocytes derived from peripheral blood and tumor fragments using the DNA Easy Blood & Tissue Kit (Qiagen) according to the manufacturer’s protocol. DNA libraries were prepared using the Illumina Paired-End Sample Prep Kit according to the manufacturer’s protocol. Target enrichment was performed using the Agilent SureSelect Human Exon Kit 50 Mb capture set (Agilent, G3362). Sequencing with 75-base paired-end reads of targeted-enrichment libraries was performed on the HiSeq 2000 sequencer. Reads were mapped to the Sanger human reference (hg19) by bwa (Li & Durbin, 2010) 0–7.5 with default settings. Overview of median coverage and the percentage with > 10× and 30× coverage is provided in Supplementary Table S1. BAM files were processed using Picard [1.101], SAMtools [0.1.18] (Li *et al*, 2009) and the Genome Analysis ToolKit (GATK) release 2.7–4 (DePristo *et al*, 2011). In brief, BAM files were binary compressed, sorted, and indexed by SAMtools (samtools view, sort, and index tools), duplicated reads were removed by Picard (with MarkDuplicates), and base quality score recalibration and local realignment around indels followed the recommended workflow of the GATK toolkit (RealignerTargetCreator, IndelRealigner, BaseRecalibrator and PrintReads). Variants were called by GATK 2.7–4 using the “UnifiedGenotyper” with default settings except for “-minIndelFrac” which was set to 10%. Annotation of the vcf files was performed with ANNOVAR (Wang *et al*, 2010). All variants detected in the germ line (blood) samples with a variant allele frequency (VAF) over 5% were excluded from further analysis. Variants were further filtered: minimum VAF of 5% in at least one of the samples; a minimum of 10x coverage in a least one of the samples; variant positions must not be listed as a single nucleotide polymorphism (SNP) in the 1,000 Genome project except when present in COSMIC; variant position must be annotated as exonic by RefSeq (Release 45); and synonymous/non-synonymous calls were made and the synonymous excluded from further analysis. All filtering was performed with R 3.0.1 using in-house parsers. The sequencing data have been made available through the European Genome-phenome Archive (EGA; http://www.ebi.ac.uk/ega/home; accession number EGAS00001000415).

### Copy number detection

DNA copy number profiles were generated using CopywriteR as described in (Kuilman *et al*, 2015). In short, sequence reads outside the captured genomic regions (off-target reads) were used to generate DNA copy number profiles. A depth-of-coverage method was used for 20-kb bins, and the read count was normalized for GC content and mappability. Log2-transformed ratios were calculated for all tumor samples versus reference (blood) sample. The normalized and corrected profiles were further analyzed by circular binary segmentation (CBS) (Olshen *et al*, 2004).

### PCR and qPCR on gDNA

qPCR was performed as described previously (Vredeveld *et al*, 2012). The following primers were used: LINE-F: 5′-AAAGCCGCTCAACTACATGG-3′; LINE-R: 5′-TGCTTTGAATGCGTCCCAGAG-3′; CRAF-F: 5′-CAACTGATTGCACTGACTGCCAAC-3′; CRAF-R: 5′-CCAGCTTTCTACTCACCGCACAAC-3′; BRAF-F: 5′-CAAGTCACCACAAAAACCTATCGT-3′; and BRAF-R: 5′- AACTGACTCACCACTGTCCTCTGTT-3′. The PCR to detect the MEK1 insertion was performed on gDNA with the following primers: MEK1-exon2-F: 5′-TGATGAGCAGCAGCGAAAGC-3′; MEK1-exon2-R: GAACACCACACCGCCATTGC-3′; MEK1^T55delinsRT^ -F: 5′-CCTTGAGGCCTTTCTTAGAA-3′.

### *MEK1*^*T55delinsRT*^ cloning

The *MEK1*^*T55delinsRT*^ was cloned in a two-step PCR from the pLZRS-MEK1-WT (Addgene plasmid 21196). The first PCR was performed with the following primers: MEK1-cloning-F: 5′- CACCATGCCCAAGAAGAAGCG -3′ and MEK1-ins3 bp-R: 5′-CTGCTTCTGGGTTCTAAGAAAAGGCCTCAAGGCGCT-3′, and the PCR product was isolated by gel extraction and used in the second PCR as a forward primer in combination with the following primer: MEK1-cloning-R: 5′- TTAGACGCCAGCAGCATGGGT-3′. The PCR product was gel-purified and cloned into the pENTR plasmid (K2400-20, Invitrogen) according to the manufacturers’ protocol. MEK1^WT^ was cloned from the pLZRS-MEK1-WT into the pDONR™223 plasmid (Invitrogen) with Gateway BP clonase™ II (11789-20, Invitrogen) according to the manufacturers’ protocol. Lastly, the *MEK1*^*WT*^ and *MEK1*^*T55delinsRT*^ were cloned into the retroviral pQCXIP vector by using Gateway LR clonase™ II (11791-20, Invitrogen) according to the manufacturers’ protocol.

### Cell culture, retroviral and lentiviral infections, colony formation assays, and dose–response curves and inhibitors

Melanoma cell lines (A-375, SK-MEL-28, and 888Mel) were cultured in DMEM supplemented with 9% fetal bovine serum and 2 mM glutamine. Retroviral and lentiviral infections were performed as described previously (Vredeveld *et al*, 2012). Cells were selected with 1.5 μg/ml puromycin for at least 48 hours before the start of the experiments. Colony formation assays were performed in 6-well plates by plating 7,500 cells (A375-*GFP*), 60,000 cells (888Mel- *MEK1*^*T55delinsRT*^), or 10,000 cells (the rest) and refreshing them with drug-containing medium the next day. Drugs were replenished twice per week. Plates were stained with crystal violet (1% dissolved in 50% methanol and 50% H_2_0) between 8 and 10 days after the start of the experiment. Dose–response curves were performed as follows: 3,000–6,000 cells were plated per well in a 96-well plate. For each condition, triplicates were plated. The next day, drug was added to the wells. At day 5 of the assay, medium-containing drug was removed and survival was measured by CellTiter-Blue Cell Viability Assay (G8081, Promega), according to the manufacturer’s protocol. The following inhibitors were used: dabrafenib (GSK2118436, Abmole), vemurafenib (PLX4032, S1267, Selleck Chemicals), trametinib (GSK1120212, S2673, Selleck Chemicals), SCH772984 (Merck), PD-0325901 (S1036, Selleck Chemicals), U0126, and selumetinib (AZD6244, S1008, Selleck Chemicals).

## Supporting Information

**Supplementary Information**

**Review Process File**
