# Supplementary material for: Intra- and inter-tumor heterogeneity in a vemurafenib-resistant melanoma patient and derived xenografts
Source: EMBO Mol Med. 2015 Jun 23;7(9):1104–18. doi: 10.15252/emmm.201404914 (PMC4568946; doi:10.15252/emmm.201404914)
Supplement: Supplementary file 1 [file emmm0007-1104-sd1.pdf]

**Figure S1**

Control staining for p-ERK performed on normal human skin. Here, for comparison, p-ERK stainings for M032 and M032R1 are included. Scale bar represents 100 μm.

**Figure S2**

Amplicons containing BRAF differ between distinct metastases. BRAF is amplified in three of the vemurafenib-resistant metastases (M032R1, M032R2 and M032R5), but the size of the amplicons differs among them, suggesting that the break points were established through independent events.

(**A**) Copy number profiles for the genomic region on chromosome 7 where *BRAF* is located. Blue lines indicate the break points of the amplicons in M032R1, which are different from those in M032R2 and M032R5. (**B**) Segmented $log_2$ratios for the genomic region with amplification of *BRAF* in M032R1, M032R2 and M032R5. Red represents a gain, blue represents a loss.

**Figure S3**

$MEK1^{T55delinsRT}$ mutation confers resistance to vemurafenib, but not to trametinib in 888mel melanoma cells.

(**A**) Validation of expression of pQXCIP-GFP, $MEK1^{WT}$ or $MEK1^{T55delinsRT}$ in 888mel melanoma cells by immunoblotting. (**B**) Dose-response curves for 888mel cells with lentivirus-encoding GFP, $MEK1^{WT}$ or $MEK1^{T55delinsRT}$ with different doses of BRAF inhibitor vemurafenib, ERK inhibitor SCH772984 or MEK inhibitor trametinib. Error bars indicated standard deviation. (**C**) Colony formation assays with 888mel cells, infected with GFP, $MEK1^{WT}$ or $MEK1^{T55delinsRT}$, treated with indicated doses and inhibitors. (**D**) Treatment of 888mel $MEK1^{T55delinsRT}$ melanoma cells DMSO (-), 250

nM dabrafenib (D), 25 nM trametinib (T) or a combination (D+T). (**E**) Immunoblotting of *MEK1*$^{WT}$ and *MEK1*$^{T55delinsRT}$ 888mel melanoma cells for players of the MAPK pathway after 24h of ERK inhibitor, with doses as indicated in the figure. Phosphorylation of ERK is inhibited when using higher doses of ERK inhibitor. Normalized band densities for p-ERK and p-RSK are displayed in the graphs. (**F**) Immunoblotting of *MEK1*$^{WT}$ and *MEK1*$^{T55delinsRT}$ 888mel melanoma cells for players of the MAPK pathway after 24h of vemurafenib, with doses as indicated in the figure. Phosphorylation of ERK remains relatively high despite inhibition with high doses of BRAF inhibitor. Normalized band densities for p-ERK and p-RSK are displayed in the graphs.

**Figure S4**

*MEK1*$^{T55delinsRT}$ mutation confers resistance to vemurafenib, but not to trametinib in SKMEL-28 melanoma cells.

(**A**) Validation of expression of pQXCIP-GFP, MEK1$^{WT}$ or MEK1$^{T55delinsRT}$ in SKMEL-28 melanoma cells by immunoblotting. (**B**) Dose-response curves for SKMEL-28 cells with GFP, MEK1$^{WT}$ or MEK1$^{T55delinsRT}$ with different doses of BRAF inhibitor vemurafenib, ERK inhibitor SCH772984 or MEK inhibitor trametinib. Error bars indicated standard deviation. (**C**) Colony formation assays with SKMEL-28 cells, infected with lentivirus-encoding GFP, MEK1$^{WT}$ or MEK1$^{T55delinsRT}$, treated with indicated doses and inhibitors. (**D**) Treatment of SKMEL-28 *MEK1*$^{T55delinsRT}$ melanoma cells with DMSO (-), 250 nM dabrafenib (D), 25 nM trametinib (T) or a combination (D+T). (**E**) Immunoblotting of *MEK1*$^{WT}$ and *MEK1*$^{T55delinsRT}$ SKMEL-28 melanoma cells for players of the MAPK pathway after 24h of ERK inhibitor, with doses as indicated in the figure. Normalized band densities for p-ERK and p-RSK are

displayed in the graphs. (**F**) Immunoblotting of *MEK1*$^{WT}$ and *MEK1*$^{T55delinsRT}$ SKMEL-28 melanoma cells for players of the MAPK pathway after 24h of vemurafenib, with doses as indicated in the figure. Normalized band densities for p-ERK and p-RSK are displayed in the graphs.

**Figure S5**

Melanoma cells carrying the *MEK1*$^{T55delinsRT}$ mutation have hyperactivated MAPK-pathway which inhibits their growth capacity.

(**A**) Immunoblotting of *MEK1*$^{WT}$ and *MEK1*$^{T55delinsRT}$ A375 melanoma cells for players of the MAPK pathway after 24h of ERK inhibitor, with doses as indicated in the figure. Phosphorylation of ERK and RSK is inhibited when using higher doses of ERK inhibitor. Normalized band densities for p-ERK and p-RSK are displayed in the graphs. (**B**) Immunoblotting of *MEK1*$^{WT}$ and *MEK1*$^{T55delinsRT}$ A375 melanoma cells for players of the MAPK pathway after 24h of vemurafenib, with doses as indicated in the figure. Phosphorylation of both ERK and RSK remains relatively high despite inhibition with high doses of BRAF inhibitor. Normalized band densities for p-ERK and pRSK are displayed in the graphs.

**Figure S6**

*MEK1*$^{T55delinsRT}$ mutation gives resistance to several MEK inhibitors.

(**A**) A375 melanoma cells, retrovirally transduced with GFP, MEK1$^{WT}$ and MEK1$^{T55delinsRT}$, were treated with different doses (as indicated) of the MEK inhibitors PD-0325901, U0126 and selumetinib. (**B**) 888mel melanoma cells, retrovirally transduced with GFP, MEK1$^{WT}$ and MEK1$^{T55delinsRT}$, were treated with different doses (as indicated) of the MEK inhibitors PD-0325901, U0126 and selumetinib. (**C**)

SKMEL-28 melanoma cells, retrovirally transduced with GFP, $MEK1^{WT}$ and $MEK1^{T55delinsRT}$, were treated with different doses (as indicated) of the MEK inhibitors PD-0325901, U0126 and selumetinib.

**Figure S7**

Heterogeneity of the BRAF amplification across different vemurafenib-resistant metastases.

(**A**) Stainings for $BRAF^{V600E}$ of the patient's pre-treatment tumor and the five resistant metastases. Staining is heterogeneous in M032R1 and M032R3, indicating that there could be several populations of tumor cells with either $BRAF^{V600E}$ amplification or an alternative resistance mechanism. Especially for M032R3, two different pieces of the same tumor showed a very different intensity in staining. Scale bar in upper row represents 2 mm, in the lower row 600 μm. (**B**) Stainings for $BRAF^{V600E}$ on PDX derived from the pre-treatment tumor and four resistant metastases. Staining was very heterogeneous in one PDX derived of M032R2, which showed dispersed areas of intense and very light staining, indicating heterogeneity of the $BRAF^{V600E}$ amplification. Scale bar in upper row represents 2 mm, in the lower row 600 μm. (**C**) An independent set of tumor fragments from the pre-treatment tumor and the five resistant metastases were taken for isolation of new gDNA to study heterogeneity of the resistance mechanisms. qPCR was performed for *BRAF*, *CRAF* and as a control *LINE*. Error bars indicated standard deviation. Data showed that BRAF was amplified in M032R2 and M032R5, but not M032R1.

## Figure S1

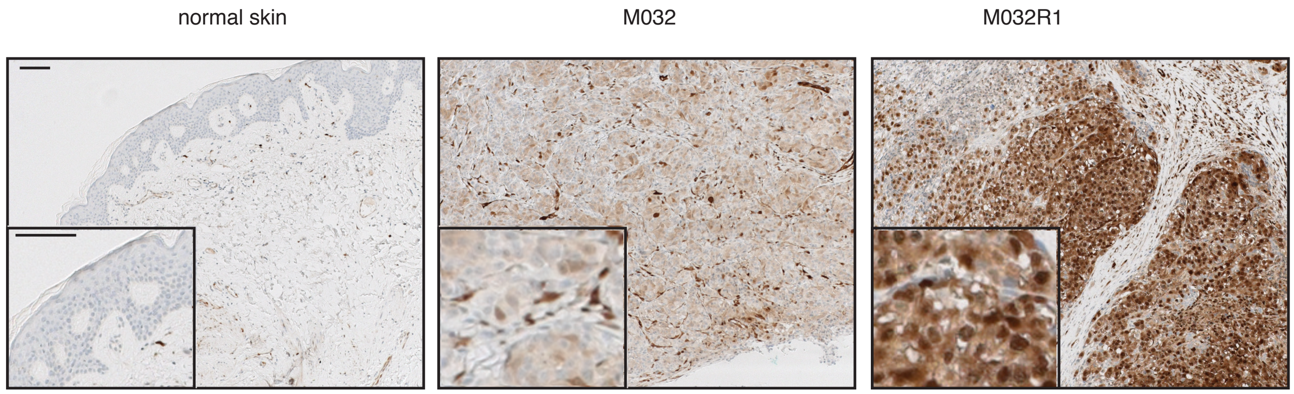

**Figure S2**

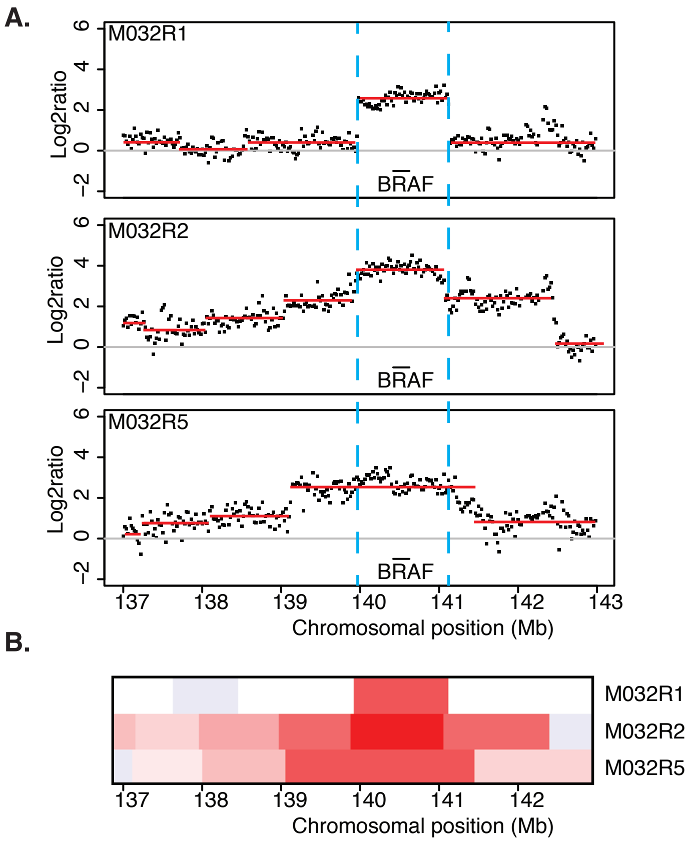
A.
M032R1
M032R2
M032R5
Log2ratio
6
4
2
0
−2
BRAF
137
138
139
140
141
142
143
Chromosomal position (Mb)
B.
M032R1
M032R2
M032R5
137
138
139
140
141
142
Chromosomal position (Mb)

## Figure S3

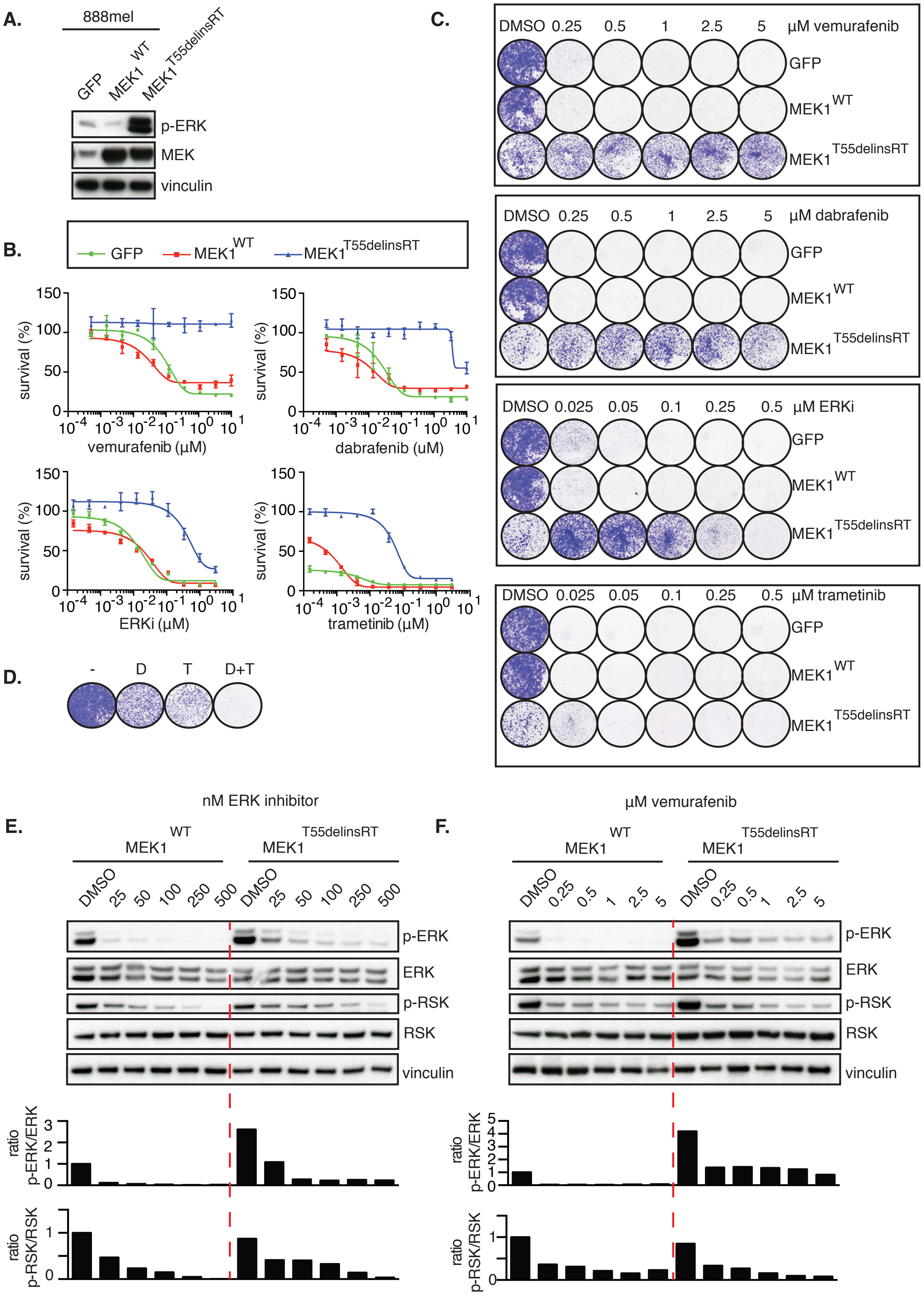

A.
888mel
GFP
MEK1 WT
MEK1 T55delinsRT
p-ERK
MEK
vinculin
B.
GFP
MEK1 WT
MEK1 T55delinsRT
survival (%)
vemurafenib (μM)
dabrafenib (uM)
ERKi (μM)
trametinib (μM)
C.
DMSO 0.25 0.5 1 2.5 5 μM vemurafenib
DMSO 0.25 0.5 1 2.5 5 μM dabrafenib
DMSO 0.025 0.05 0.1 0.25 0.5 μM ERKi
DMSO 0.025 0.05 0.1 0.25 0.5 μM trametinib
D.
- D T D+T
E.
nM ERK inhibitor
DMSO 25 50 100 250 500
p-ERK
ERK
p-RSK
RSK
vinculin
ratio p-ERK/ERK
ratio p-RSK/RSK
F.
μM vemurafenib
DMSO 0.25 0.5 1 2.5 5

Figure S4

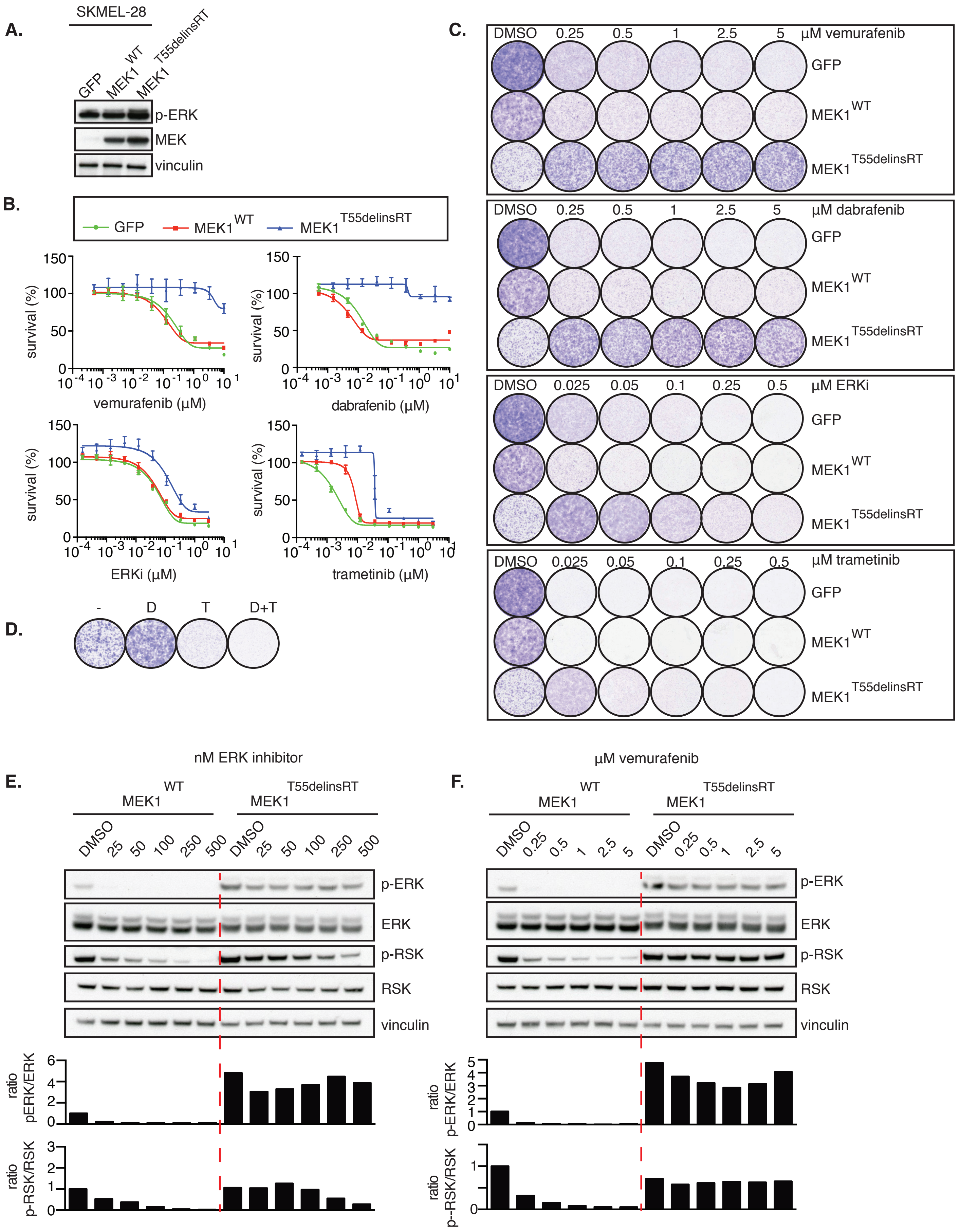

A.
SKMEL-28
GFP
MEK1 WT
MEK1 T55delinsRT
p-ERK
MEK
vinculin
B.
GFP
MEK1 WT
MEK1 T55delinsRT
survival (%)
vemurafenib (µM)
dabrafenib (µM)
ERKi (µM)
trametinib (µM)
C.
DMSO
0.25
0.5
1
2.5
5
µM vemurafenib
µM dabrafenib
0.025
0.05
0.1
µM ERKi
µM trametinib
GFP
MEK1 WT
MEK1 T55delinsRT
D.
-
D
T
D+T
E.
nM ERK inhibitor
MEK1 WT
MEK1 T55delinsRT
DMSO
25
50
100
250
500
p-ERK
ERK
p-RSK
RSK
vinculin
ratio pERK/ERK
ratio p-RSK/RSK
F.
µM vemurafenib
ratio p-ERK/ERK
ratio p--RSK/RSK

## Figure S5

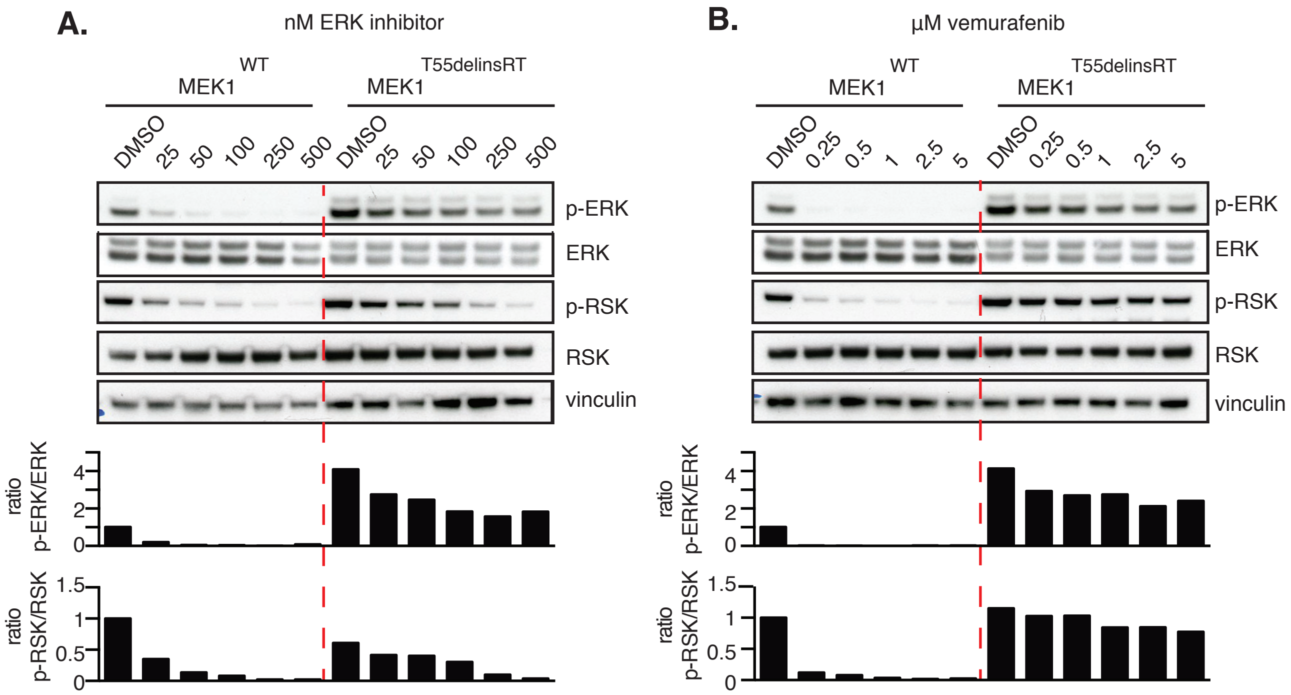
A.
nM ERK inhibitor
MEK1
WT
T55delinsRT
DMSO
25
50
100
250
500
p-ERK
ERK
p-RSK
RSK
vinculin
ratio
p-ERK/ERK
ratio
p-RSK/RSK
B.
µM vemurafenib
0.25
0.5
1
2.5
5

## Figure S6

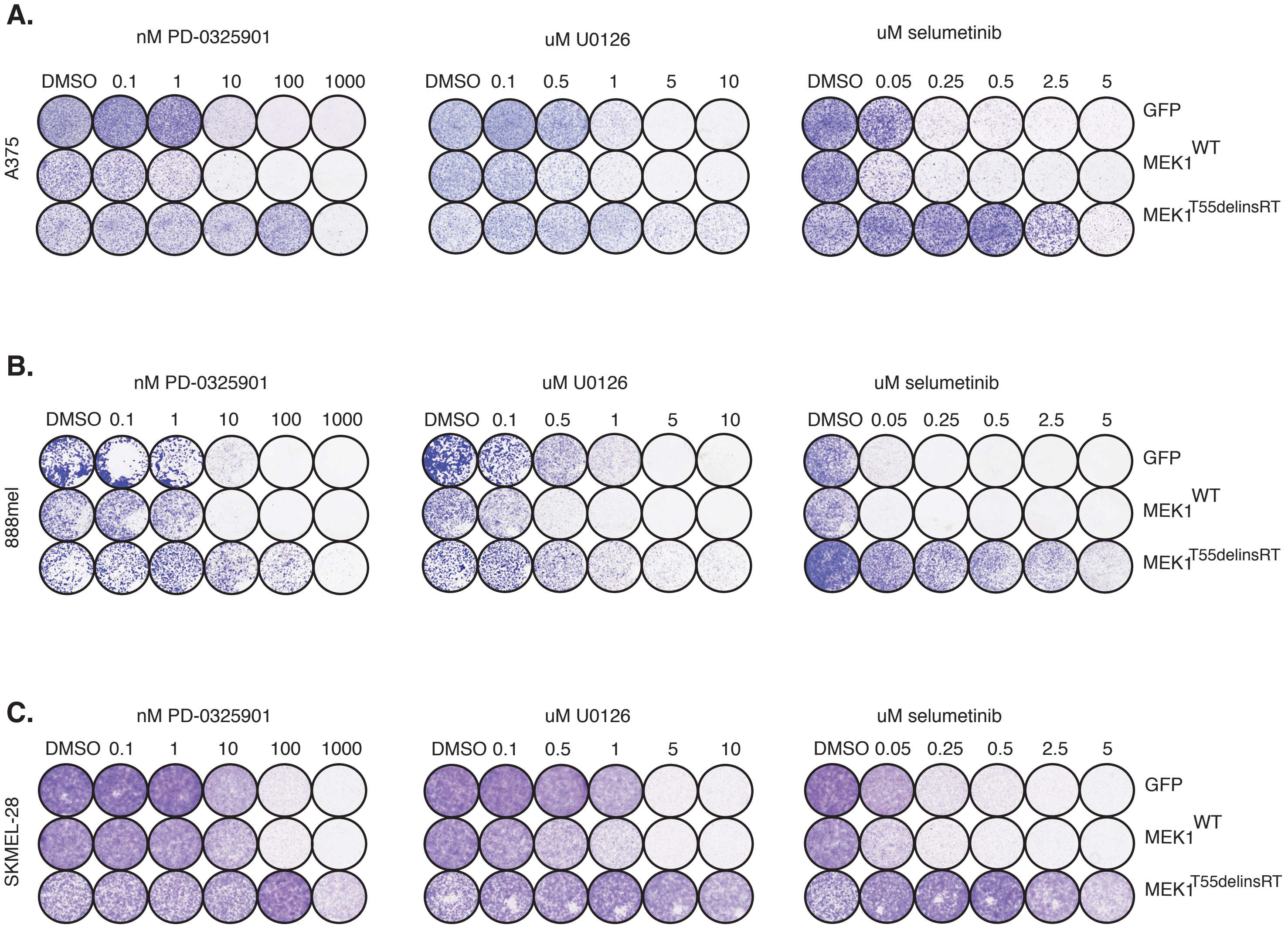
A.
nM PD-0325901
DMSO 0.1 1 10 100 1000
uM U0126
DMSO 0.1 0.5 1 5 10
uM selumetinib
DMSO 0.05 0.25 0.5 2.5 5
A375
GFP
MEK1WT
MEK1T55delinsRT
B.
nM PD-0325901
DMSO 0.1 1 10 100 1000
uM U0126
DMSO 0.1 0.5 1 5 10
uM selumetinib
DMSO 0.05 0.25 0.5 2.5 5
888mel
GFP
MEK1WT
MEK1T55delinsRT
C.
nM PD-0325901
DMSO 0.1 1 10 100 1000
uM U0126
DMSO 0.1 0.5 1 5 10
uM selumetinib
DMSO 0.05 0.25 0.5 2.5 5
SKMEL-28
GFP
MEK1WT
MEK1T55delinsRT

**Figure S7**

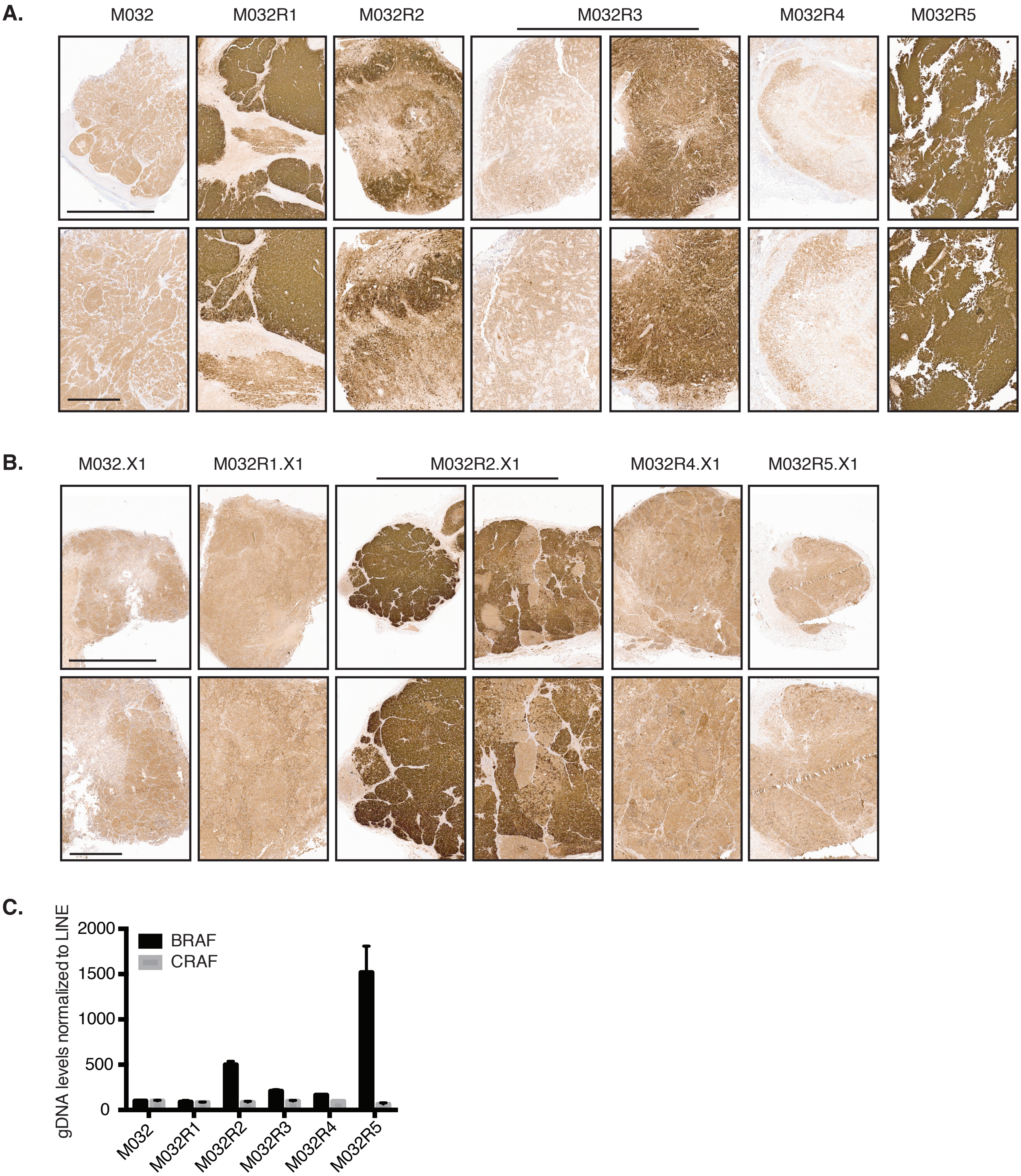
A.
M032
M032R1
M032R2
M032R3
M032R4
M032R5
B.
M032.X1
M032R1.X1
M032R2.X1
M032R4.X1
M032R5.X1
C.
gDNA levels normalized to LINE
2000
1500
1000
500
0
BRAF
CRAF
M032
M032R1
M032R2
M032R3
M032R4
M032R5

**Table S1**: Sequence coverage, median, 10x, 20x, 30x

| Sample | Median coverage | >10x | >20x | >30x |
|---|---|---|---|---|
| Blood | 65x | 0.872 | 0.777 | 0.698 |
| Pre | 41x | 0.810 | 0.681 | 0.582 |
| R1 | 46x | 0.825 | 0.706 | 0.614 |
| R2 | 35x | 0.794 | 0.652 | 0.543 |
| R3 | 43x | 0.816 | 0.691 | 0.597 |
| R5 | 32x | 0.764 | 0.617 | 0.508 |
| R6 | 46x | 0.829 | 0.708 | 0.615 |

## Table S2

| Func | Gene | ExonicFunc | AAChange | Chr | Start | End | Ref | Obs | Pre | R1 | R2 | R3 | R4 | R5 |
|---|---|---|---|---|---|---|---|---|---|---|---|---|---|---|
| exonic | C1orf86 | stoploss SNV | NM_001256946:c.A588G:p.X196W | 1 | 2124297 | 2124297 | T | C | 0,00 | 0,00 | 0,12 | 0,17 | 0,08 | 0,00 |
| exonic | CHD5 | nonsynonymous SNV | NM_015557:c.G3606T:p.K1202N | 1 | 6188911 | 6188911 | C | A | 0,32 | 0,32 | 0,42 | 0,31 | 0,20 | 0,18 |
| exonic | KIF17 | nonsynonymous SNV | NM_001122819:c.G2672A:p.G891D | 1 | 20998481 | 20998481 | C | T | 0,26 | 0,39 | 0,53 | 0,19 | 0,13 | 0,21 |
| exonic | CSMD2 | nonsynonymous SNV | NM_052896:c.G10129A:p.G3377S | 1 | 33987099 | 33987099 | C | T | 0,21 | 0,39 | 0,43 | 0,37 | 0,21 | 0,22 |
| exonic | DLGAP3 | nonsynonymous SNV | NM_001080418:c.A2287C:p.T763P | 1 | 35334404 | 35334404 | T | G | 0,50 | 0,20 | 0,30 | 0,21 | 0,32 | 0,29 |
| exonic | RAD54L | nonsynonymous SNV | NM_003579:c.C1600A:p.R534S | 1 | 46739409 | 46739409 | C | A | 0,36 | 0,00 | 0,00 | 0,00 | 0,00 | 0,00 |
| exonic | CC2D1B | nonsynonymous SNV | NM_032449:c.C1864A:p.Q622K | 1 | 52822705 | 52822705 | G | T | 0,67 | 0,00 | 0,00 | 0,00 | 0,00 | 0,00 |
| exonic | DPYD | nonsynonymous SNV | NM_001160301:c.C514T:p.H172Y | 1 | 98186452 | 98186452 | G | A | 0,32 | 0,41 | 0,50 | 0,27 | 0,25 | 0,23 |
| exonic | ADAM30 | nonsynonymous SNV | NM_021794:c.G929A:p.G310E | 1 | 120438031 | 120438031 | C | T | 0,33 | 0,40 | 0,36 | 0,22 | 0,07 | 0,14 |
| exonic | RPTN | nonsynonymous SNV | NM_001122965:c.A460G:p.R154G | 1 | 152129115 | 152129115 | T | C | 0,10 | 0,08 | 0,11 | 0,06 | 0,16 | 0,15 |
| exonic | FLG | nonsynonymous SNV | NM_002016:c.G11692A:p.G3898S | 1 | 152275670 | 152275670 | C | T | 0,26 | 0,37 | 0,22 | 0,12 | 0,27 | 0,21 |
| exonic | ARHGEF11 | nonsynonymous SNV | NM_014784:c.G229T:p.A77S | 1 | 156950273 | 156950273 | C | A | 0,00 | 0,00 | 0,00 | 0,00 | 0,00 | 0,44 |
| exonic | SELP | nonsynonymous SNV | NM_003005:c.A1332T:p.Q444H | 1 | 169578743 | 169578743 | T | A | 0,00 | 0,04 | 0,00 | 0,00 | 0,00 | 0,14 |
| exonic | PRG4 | nonsynonymous SNV | NM_001127710:c.G1486A:p.E496K | 1 | 186276739 | 186276739 | G | A | 0,00 | 0,04 | 0,02 | 0,00 | 0,14 | 0,00 |
| exonic | PTGS2 | stopgain SNV | NM_000963:c.C721T:p.Q241X | 1 | 186645967 | 186645967 | G | A | 0,30 | 0,28 | 0,28 | 0,15 | 0,15 | 0,19 |
| exonic | PIK3C2B | nonsynonymous SNV | NM_002646:c.G4729T:p.G1577W | 1 | 204394156 | 204394156 | C | A | 0,00 | 0,04 | 0,00 | 0,00 | 0,36 | 0,00 |
| exonic | DNAH14 | nonsynonymous SNV | NM_001373:c.C2776G:p.P926A | 1 | 225268090 | 225268090 | C | G | 0,00 | 0,25 | 0,31 | 0,15 | 0,12 | 0,15 |
| exonic | FMN2 | nonframeshift deletion | NM_020066:c.658_660del:p.220_220del | 1 | 240256067 | 240256069 | CAG | - | 0,00 | 0,00 | 0,25 | 0,00 | 0,00 | 0,15 |
| exonic | OR2M3 | nonsynonymous SNV | NM_001004689:c.G922A:p.G308S | 1 | 248367291 | 248367291 | G | A | 0,20 | 0,22 | 0,20 | 0,14 | 0,11 | 0,17 |
| exonic | SH3YL1 | nonsynonymous SNV | NM_001159597:c.A614G:p.E205G | 2 | 231111 | 231111 | T | C | 0,27 | 0,35 | 0,26 | 0,14 | 0,07 | 0,11 |
| exonic | GAREML | nonsynonymous SNV | NM_001168241:c.C1838T:p.P613L | 2 | 26410339 | 26410339 | C | T | 0,33 | 0,44 | 0,38 | 0,22 | 0,23 | 0,22 |
| exonic | TMEM247 | nonsynonymous SNV | NM_001145051:c.A458G:p.Q153R | 2 | 46707884 | 46707884 | A | G | 0,21 | 0,23 | 0,00 | 0,33 | 0,18 | 0,20 |
| exonic | STON1,STON1-GTF2A1L | nonsynonymous SNV | NM_001198594:c.C164T:p.S55F | 2 | 48807936 | 48807936 | C | T | 0,38 | 0,49 | 0,33 | 0,27 | 0,09 | 0,29 |
| exonic | NRXN1 | nonsynonymous SNV | NM_004801:c.G833A:p.G278E | 2 | 50850753 | 50850753 | C | T | 0,00 | 0,35 | 0,37 | 0,24 | 0,09 | 0,11 |
| exonic | NRXN1 | nonsynonymous SNV | NM_004801:c.G832A:p.G278R | 2 | 50850754 | 50850754 | C | T | 0,00 | 0,35 | 0,34 | 0,23 | 0,09 | 0,11 |
| exonic | STARD7 | stopgain SNV | NM_020151:c.G262T:p.E88X | 2 | 96873911 | 96873911 | C | A | 0,11 | 0,47 | 0,55 | 0,21 | 0,26 | 0,27 |
| exonic | AFF3 | nonframeshift deletion | NM_001025108:c.1330_1332del:p.444_444del | 2 | 100218011 | 100218013 | GCT | - | 0,00 | 0,22 | 0,10 | 0,18 | 0,10 | 0,18 |
| exonic | HS6ST1 | stopgain SNV | NM_004807:c.A199T:p.K67X | 2 | 129075939 | 129075939 | T | A | 0,14 | 0,17 | 0,13 | 0,16 | 0,21 | 0,14 |
| exonic | LRP1B | nonsynonymous SNV | NM_018557:c.C9265T:p.P3089S | 2 | 141243072 | 141243072 | G | A | 0,17 | 0,26 | 0,23 | 0,26 | 0,08 | 0,00 |
| exonic | KIF5C | nonsynonymous SNV | NM_004522:c.G2716A:p.E906K | 2 | 149866814 | 149866814 | G | A | 0,02 | 0,45 | 0,40 | 0,12 | 0,08 | 0,25 |
| exonic | TTN | stopgain SNV | NM_133379:c.C16321T:p.R5441X | 2 | 179610806 | 179610806 | G | A | 0,18 | 0,30 | 0,38 | 0,27 | 0,09 | 0,18 |
| exonic | ITGAV | frameshift deletion | NM_001145000:c.1357delA:p.K453fs | 2 | 187516776 | 187516776 | A | - | 0,06 | 0,24 | 0,21 | 0,08 | 0,11 | 0,17 |
| exonic | ITGAV | nonsynonymous SNV | NM_001145000:c.A1360G:p.T454A | 2 | 187516779 | 187516779 | A | G | 0,04 | 0,23 | 0,19 | 0,07 | 0,09 | 0,16 |
| exonic | ANKAR | nonsynonymous SNV | NM_144708:c.C2947A:p.Q983K | 2 | 190593062 | 190593062 | C | A | 0,26 | 0,28 | 0,36 | 0,17 | 0,15 | 0,26 |
| exonic | DNAH7 | nonsynonymous SNV | NM_018897:c.G5109A:p.M1703I | 2 | 196753643 | 196753643 | C | T | 0,24 | 0,29 | 0,27 | 0,16 | 0,12 | 0,18 |
| exonic | CPS1 | nonsynonymous SNV | NM_001122634:c.C2435T:p.S812F | 2 | 211525240 | 211525240 | C | T | 0,00 | 0,02 | 0,00 | 0,00 | 0,01 | 0,12 |
| exonic | TNS1 | nonsynonymous SNV | NM_022648:c.G2321A:p.G774E | 2 | 218712544 | 218712544 | C | T | 0,35 | 0,25 | 0,51 | 0,09 | 0,13 | 0,37 |
| exonic | DAW1 | nonsynonymous SNV | NM_178821:c.G631A:p.E211K | 2 | 228767808 | 228767808 | G | A | 0,22 | 0,36 | 0,28 | 0,27 | 0,13 | 0,22 |
| exonic | HTR2B | nonsynonymous SNV | NM_000867:c.G1408A:p.E470K | 2 | 231973269 | 231973269 | C | T | 0,00 | 0,03 | 0,00 | 0,00 | 0,00 | 0,16 |
| exonic | RNPEPL1 | nonsynonymous SNV | NM_018226:c.C1949T:p.A650V | 2 | 241517080 | 241517080 | C | T | 0,29 | 0,00 | 0,00 | 0,00 | 0,00 | 0,00 |
| exonic | ENTPD3 | nonsynonymous SNV | NM_001248:c.G980A:p.G327E | 3 | 40464489 | 40464489 | G | A | 0,24 | 0,33 | 0,28 | 0,19 | 0,21 | 0,14 |
| exonic | TRAK1 | nonsynonymous SNV | NM_001042646:c.G2287A:p.G763S | 3 | 42264654 | 42264654 | G | A | 0,14 | 0,00 | 0,00 | 0,00 | 0,00 | 0,00 |
| exonic | ZNF445 | nonsynonymous SNV | NM_181489:c.T1638G:p.C546W | 3 | 44489525 | 44489525 | A | C | 0,26 | 0,34 | 0,20 | 0,15 | 0,08 | 0,25 |
| exonic | SETD2 | nonsynonymous SNV | NM_014159:c.C374T:p.S125F | 3 | 47165752 | 47165752 | G | A | 0,29 | 0,30 | 0,45 | 0,23 | 0,19 | 0,24 |
| exonic | MAP4 | nonsynonymous SNV | NM_001134364:c.G530A:p.G177D | 3 | 47960331 | 47960331 | C | T | 0,00 | 0,23 | 0,38 | 0,34 | 0,11 | 0,16 |
| exonic | PARP3 | nonsynonymous SNV | NM_001003931:c.C1390T:p.P464S | 3 | 51981848 | 51981848 | C | T | 0,20 | 0,26 | 0,15 | 0,14 | 0,10 | 0,15 |
| exonic | TLR9 | nonsynonymous SNV | NM_017442:c.C2674T:p.R892W | 3 | 52255658 | 52255658 | G | A | 0,25 | 0,36 | 0,24 | 0,22 | 0,35 | 0,18 |
| exonic | SPATA12 | nonsynonymous SNV | NM_181727:c.A562G:p.T188A | 3 | 57108284 | 57108284 | A | G | 0,21 | 0,35 | 0,07 | 0,14 | 0,12 | 0,19 |
| exonic | FLNB | nonsynonymous SNV | NM_001457:c.G5168A:p.G1723E | 3 | 58127643 | 58127643 | G | A | 0,32 | 0,35 | 0,18 | 0,22 | 0,11 | 0,13 |
| exonic | EPHA6 | nonsynonymous SNV | NM_001080448:c.A446G:p.N149S | 3 | 96585729 | 96585729 | A | G | 0,38 | 0,29 | 0,29 | 0,08 | 0,14 | 0,21 |
| exonic | UROC1 | nonsynonymous SNV | NM_001165974:c.G379A:p.G127R | 3 | 126227291 | 126227291 | C | T | 0,00 | 0,31 | 0,00 | 0,00 | 0,00 | 0,00 |
| exonic | ASTE1 | frameshift insertion | NM_014065:c.1895_1896insA:p.R632fs | 3 | 130733046 | 130733046 | - | T | 0,17 | 0,02 | 0,10 | 0,08 | 0,10 | 0,00 |
| exonic | TRIM42 | nonsynonymous SNV | NM_152616:c.C220T:p.P74S | 3 | 140397291 | 140397291 | C | T | 0,35 | 0,16 | 0,32 | 0,19 | 0,22 | 0,25 |
| exonic | PRKCI | nonsynonymous SNV | NM_002740:c.C1438T:p.R480C | 3 | 170013719 | 170013719 | C | T | 0,29 | 0,30 | 0,27 | 0,21 | 0,13 | 0,21 |

| | | | | | | | | | | | | | | |
|---|---|---|---|---|---|---|---|---|---|---|---|---|---|---|
| exonic | ATP13A4 | nonsynonymous SNV | NM_032279:c.C1088T:p.T363I | 3 | 193185131 | 193185131 | G | A | 0,21 | 0,26 | 0,03 | 0,17 | 0,13 | 0,22 |
| exonic | MFSD7 | nonsynonymous SNV | NM_032219:c.A1327C:p.T443P | 4 | 676100 | 676100 | T | G | 0,20 | 0,11 | 0,17 | 0,08 | 0,29 | 0,25 |
| exonic | YIPF7 | nonsynonymous SNV | NM_182592:c.C587T:p.S196L | 4 | 44626711 | 44626711 | G | A | 0,21 | 0,37 | 0,48 | 0,25 | 0,33 | 0,26 |
| exonic | FRAS1 | nonsynonymous SNV | NM_025074:c.C9619T:p.P3207S | 4 | 79429999 | 79429999 | C | T | 0,27 | 0,32 | 0,33 | 0,20 | 0,22 | 0,28 |
| exonic | PCDH18 | nonsynonymous SNV | NM_019035:c.C509T:p.S170F | 4 | 138452734 | 138452734 | G | A | 0,28 | 0,36 | 0,23 | 0,13 | 0,15 | 0,14 |
| exonic | SEMA5A | nonsynonymous SNV | NM_003966:c.G2311A:p.D771N | 5 | 9063206 | 9063206 | C | T | 0,30 | 0,53 | 0,67 | 0,31 | 0,33 | 0,27 |
| exonic | DNAH5 | nonsynonymous SNV | NM_001369:c.C8537T:p.A2846V | 5 | 13788935 | 13788935 | G | A | 0,18 | 0,04 | 0,00 | 0,00 | 0,00 | 0,10 |
| exonic | EPB41L4A | nonsynonymous SNV | NM_022140:c.G432T:p.Q144H | 5 | 111601931 | 111601931 | C | A | 0,00 | 0,00 | 0,44 | 0,00 | 0,00 | 0,00 |
| exonic | AP3S1 | frameshift deletion | NM_001284:c.121_124del:p.41_42del | 5 | 115202418 | 115202421 | AAGA | - | 0,03 | 0,11 | 0,17 | 0,02 | 0,05 | 0,08 |
| exonic | FNIP1 | nonsynonymous SNV | NM_001008738:c.C2657T:p.P886L | 5 | 131007396 | 131007396 | G | A | 0,11 | 0,00 | 0,00 | 0,00 | 0,00 | 0,00 |
| exonic | PCDHA3 | nonsynonymous SNV | NM_018906:c.G931A:p.E311K | 5 | 140181713 | 140181713 | G | A | 0,17 | 0,05 | 0,00 | 0,01 | 0,00 | 0,08 |
| exonic | PCDHB1 | nonsynonymous SNV | NM_013340:c.C2326T:p.P776S | 5 | 140433381 | 140433381 | C | T | 0,19 | 0,40 | 0,32 | 0,26 | 0,08 | 0,19 |
| exonic | PCDHGB5 | nonsynonymous SNV | NM_018925:c.G2225A:p.G742E | 5 | 140779919 | 140779919 | G | A | 0,20 | 0,03 | 0,00 | 0,00 | 0,00 | 0,06 |
| exonic | PCDHGA10 | frameshift deletion | NM_032090:c.2467delA:p.K823fs | 5 | 140795209 | 140795209 | A | - | 0,18 | 0,03 | 0,16 | 0,21 | 0,09 | 0,17 |
| exonic | KCTD16 | nonsynonymous SNV | NM_020768:c.G311A:p.G104E | 5 | 143586588 | 143586588 | G | A | 0,15 | 0,00 | 0,00 | 0,00 | 0,00 | 0,04 |
| exonic | KIF4B | nonsynonymous SNV | NM_001099293:c.G3511A:p.E1171K | 5 | 154396930 | 154396930 | G | A | 0,18 | 0,43 | 0,46 | 0,42 | 0,18 | 0,39 |
| exonic | FAM196B | nonsynonymous SNV | NM_001129891:c.G1409T:p.C470F | 5 | 169308384 | 169308384 | C | A | 0,00 | 0,00 | 0,00 | 0,00 | 0,00 | 0,27 |
| exonic | GCNT2 | nonsynonymous SNV | NM_001491:c.C719T:p.S240F | 6 | 10557375 | 10557375 | C | T | 0,27 | 0,26 | 0,39 | 0,18 | 0,21 | 0,14 |
| exonic | HIST1H4L | nonsynonymous SNV | NM_003546:c.G8A:p.G3E | 6 | 27841281 | 27841281 | C | T | 0,17 | 0,31 | 0,29 | 0,24 | 0,14 | 0,25 |
| exonic | HLA-DQA1 | nonsynonymous SNV | NM_002122:c.T231A:p.F77L | 6 | 32609235 | 32609235 | T | A | 0,14 | 0,03 | 0,00 | 0,00 | 0,00 | 0,23 |
| exonic | HLA-DQA1 | nonsynonymous SNV | NM_002122:c.G233A:p.G78E | 6 | 32609237 | 32609237 | G | A | 0,14 | 0,03 | 0,00 | 0,00 | 0,00 | 0,23 |
| exonic | ITPR3 | nonsynonymous SNV | NM_002224:c.G1273A:p.D425N | 6 | 33632854 | 33632854 | G | A | 0,30 | 0,41 | 0,28 | 0,26 | 0,12 | 0,27 |
| exonic | CNPY3 | nonframeshift deletion | NM_006586:c.50_52del:p.17_18del | 6 | 42897358 | 42897360 | TGC | - | 0,38 | 0,00 | 0,00 | 0,11 | 0,07 | 0,25 |
| exonic | CYB5R4 | nonsynonymous SNV | NM_016230:c.C334T:p.H112Y | 6 | 84618731 | 84618731 | C | T | 0,33 | 0,77 | 0,50 | 0,12 | 0,18 | 0,32 |
| exonic | ROS1 | stopgain SNV | NM_002944:c.G63A:p.W21X | 6 | 117746757 | 117746757 | C | T | 0,60 | 0,56 | 0,52 | 0,18 | 0,18 | 0,28 |
| exonic | SERINC1 | nonsynonymous SNV | NM_020755:c.G783T:p.L261F | 6 | 122772876 | 122772876 | C | A | 0,16 | 0,29 | 0,06 | 0,11 | 0,19 | 0,13 |
| exonic | ARID1B | stopgain SNV | NM_017519:c.C2383T:p.Q795X | 6 | 157454212 | 157454212 | C | T | 0,26 | 0,29 | 0,35 | 0,17 | 0,08 | 0,27 |
| exonic | UNC93A | nonsynonymous SNV | NM_001143947:c.G356A:p.G119E | 6 | 167709606 | 167709606 | G | A | 0,01 | 0,27 | 0,42 | 0,15 | 0,17 | 0,17 |
| exonic | THBS2 | nonsynonymous SNV | NM_003247:c.T2734G:p.C912G | 6 | 169625279 | 169625279 | A | C | 0,17 | 0,37 | 0,51 | 0,23 | 0,14 | 0,19 |
| exonic | STK31 | nonsynonymous SNV | NM_001260504:c.C892T:p.L298F | 7 | 23776641 | 23776641 | C | T | 0,39 | 0,60 | 0,45 | 0,27 | 0,27 | 0,45 |
| exonic | INMT | nonsynonymous SNV | NM_001199219:c.A743G:p.N248S | 7 | 30795421 | 30795421 | A | G | 0,35 | 0,43 | 0,46 | 0,21 | 0,33 | 0,33 |
| exonic | NPSR1 | nonsynonymous SNV | NM_207172:c.G1027A:p.E343K | 7 | 34889178 | 34889178 | G | A | 0,20 | 0,24 | 0,35 | 0,19 | 0,09 | 0,17 |
| exonic | AOAH | nonsynonymous SNV | NM_001177507:c.C1120T:p.H374Y | 7 | 36580015 | 36580015 | G | A | 0,30 | 0,41 | 0,50 | 0,08 | 0,11 | 0,25 |
| exonic | PPIA | nonsynonymous SNV | NM_021130:c.G241A:p.E81K | 7 | 44839352 | 44839352 | G | A | 0,43 | 0,54 | 0,51 | 0,32 | 0,36 | 0,27 |
| exonic | ADCY1 | nonsynonymous SNV | NM_021116:c.G2716A:p.E906K | 7 | 45743343 | 45743343 | G | A | 0,19 | 0,25 | 0,30 | 0,16 | 0,16 | 0,18 |
| exonic | PKD1L1 | nonsynonymous SNV | NM_138295:c.C3143T:p.S1048F | 7 | 47925346 | 47925346 | G | A | 0,00 | 0,17 | 0,32 | 0,23 | 0,23 | 0,16 |
| exonic | LANCL2 | nonsynonymous SNV | NM_018697:c.G655A:p.V219M | 7 | 55467774 | 55467774 | G | A | 0,19 | 0,31 | 0,33 | 0,14 | 0,13 | 0,16 |
| exonic | ZNF107 | frameshift insertion | NM_001013746:c.2337_2338insAA:p.E779fs | 7 | 64169019 | 64169019 | - | AA | 0,00 | 0,02 | 0,20 | 0,00 | 0,08 | 0,12 |
| exonic | GTF2IRD1 | nonsynonymous SNV | NM_001199207:c.C1504T:p.R502W | 7 | 73950604 | 73950604 | C | T | 0,05 | 0,21 | 0,18 | 0,17 | 0,08 | 0,14 |
| exonic | AKAP9 | nonsynonymous SNV | NM_005751:c.C1110A:p.N370K | 7 | 91630341 | 91630341 | C | A | 0,00 | 0,00 | 0,21 | 0,06 | 0,18 | 0,10 |
| exonic | ASZ1 | nonsynonymous SNV | NM_130768:c.G413A:p.R138K | 7 | 117060244 | 117060244 | C | T | 0,17 | 0,30 | 0,22 | 0,06 | 0,17 | 0,13 |
| exonic | RBM28 | nonsynonymous SNV | NM_001166135:c.G256A:p.D86N | 7 | 127976031 | 127976031 | C | T | 0,02 | 0,00 | 0,13 | 0,00 | 0,02 | 0,05 |
| exonic | CREB3L2 | stopgain SNV | NM_194071:c.C1257G:p.Y419X | 7 | 137569754 | 137569754 | G | C | 0,00 | 0,00 | 0,13 | 0,00 | 0,01 | 0,00 |
| exonic | MKRN1 | nonsynonymous SNV | NM_001145125:c.C122G:p.A41G | 7 | 140179023 | 140179023 | G | C | 0,00 | 0,00 | 0,28 | 0,25 | 0,00 | 0,00 |
| exonic | MKRN1 | nonsynonymous SNV | NM_001145125:c.A85C:p.T29P | 7 | 140179060 | 140179060 | T | G | 0,50 | 0,11 | 0,18 | 0,50 | 0,00 | 0,00 |
| exonic | BRAF | nonsynonymous SNV | NM_004333:c.T1799A:p.V600E | 7 | 140453136 | 140453136 | A | T | 0,53 | 0,90 | 0,94 | 0,32 | 0,38 | 0,82 |
| exonic | KEL | nonsynonymous SNV | NM_000420:c.G1285A:p.E429K | 7 | 142643323 | 142643323 | C | T | 0,43 | 0,56 | 0,42 | 0,25 | 0,18 | 0,32 |
| exonic | GIMAP1,GIMAP1-GIMAP5 | nonsynonymous SNV | NM_001199577:c.G122A:p.S41N | 7 | 150417214 | 150417214 | G | A | 0,40 | 0,58 | 0,52 | 0,28 | 0,37 | 0,48 |
| exonic | PAXIP1 | nonsynonymous SNV | NM_007349:c.A74C:p.D25A | 7 | 154794566 | 154794566 | T | G | 0,07 | 0,24 | 0,25 | 0,38 | 0,20 | 0,47 |
| exonic | DEFA4 | nonsynonymous SNV | NM_001925:c.C134T:p.S45F | 8 | 6794288 | 6794288 | G | A | 0,17 | 0,21 | 0,32 | 0,17 | 0,21 | 0,10 |
| exonic | SGK223 | frameshift deletion | NM_001080826:c.3501delT:p.A1167fs | 8 | 8176384 | 8176384 | A | - | 0,39 | 0,27 | 0,33 | 0,09 | 0,14 | 0,44 |
| exonic | ADAM28 | nonsynonymous SNV | NM_014265:c.G841A:p.G281R | 8 | 24181467 | 24181467 | G | A | 0,23 | 0,30 | 0,21 | 0,16 | 0,04 | 0,12 |
| exonic | ADAM18 | nonsynonymous SNV | NM_001190956:c.C304T:p.P102S | 8 | 39467040 | 39467040 | C | T | 0,19 | 0,32 | 0,25 | 0,22 | 0,19 | 0,14 |
| exonic | ANK1 | nonsynonymous SNV | NM_000037:c.G4558A:p.E1520K | 8 | 41530410 | 41530410 | C | T | 0,10 | 0,17 | 0,50 | 0,17 | 0,40 | 0,50 |
| exonic | FAM110B | nonsynonymous SNV | NM_147189:c.C442T:p.R148W | 8 | 59059231 | 59059231 | C | T | 0,11 | 0,25 | 0,17 | 0,15 | 0,20 | 0,35 |

| | | | | | | | | | | | | | | |
|---|---|---|---|---|---|---|---|---|---|---|---|---|---|---|
| exonic | PREX2 | nonsynonymous SNV | NM_024870:c.C349T:p.R117C | 8 | 68934283 | 68934283 | C | T | 0,28 | 0,34 | 0,44 | 0,18 | 0,26 | 0,05 |
| exonic | SBSPON | nonsynonymous SNV | NM_153225:c.C566A:p.T189N | 8 | 73982151 | 73982151 | G | T | 0,30 | 0,33 | 0,49 | 0,12 | 0,13 | 0,16 |
| exonic | IL7 | nonsynonymous SNV | NM_000880:c.C71T:p.S24L | 8 | 79710383 | 79710383 | G | A | 0,26 | 0,35 | 0,21 | 0,27 | 0,10 | 0,34 |
| exonic | CNGB3 | nonsynonymous SNV | NM_019098:c.C2179G:p.Q727E | 8 | 87588283 | 87588283 | G | C | 0,00 | 0,03 | 0,09 | 0,05 | 0,14 | 0,03 |
| exonic | CNGB3 | nonsynonymous SNV | NM_019098:c.C2158G:p.Q720E | 8 | 87588304 | 87588304 | G | C | 0,00 | 0,00 | 0,15 | 0,02 | 0,05 | 0,07 |
| exonic | VPS13B | nonsynonymous SNV | NM_017890:c.C6577T:p.L2193F | 8 | 100729446 | 100729446 | C | T | 0,00 | 0,21 | 0,57 | 0,20 | 0,15 | 0,05 |
| exonic | KCNQ3 | nonsynonymous SNV | NM_004519:c.G250A:p.G84S | 8 | 133492530 | 133492530 | C | T | 0,25 | 0,67 | 0,67 | 0,36 | 0,17 | 0,08 |
| exonic | GPR20 | frameshift insertion | NM_005293:c.476_477insC:p.R159fs | 8 | 142367548 | 142367548 | - | G | 0,11 | 0,00 | 0,19 | 0,20 | 0,38 | 0,00 |
| exonic | MAPK15 | nonsynonymous SNV | NM_139021:c.C1196T:p.A399V | 8 | 144803573 | 144803573 | C | T | 0,27 | 0,26 | 0,42 | 0,19 | 0,13 | 0,04 |
| exonic | MPDZ | nonsynonymous SNV | NM_001261406:c.C2405T:p.S802F | 9 | 13186345 | 13186345 | G | A | 0,28 | 0,53 | 0,69 | 0,20 | 0,09 | 0,29 |
| exonic | DNAI1 | nonsynonymous SNV | NM_012144:c.C646T:p.P216S | 9 | 34491517 | 34491517 | C | T | 0,27 | 0,37 | 0,28 | 0,13 | 0,30 | 0,24 |
| exonic | CCL27 | nonsynonymous SNV | NM_006664:c.G89T:p.S30I | 9 | 34662395 | 34662395 | C | A | 0,00 | 0,19 | 0,00 | 0,00 | 0,00 | 0,00 |
| exonic | VCP | frameshift insertion | NM_007126:c.1848_1849insA:p.N616fs | 9 | 35059646 | 35059646 | - | T | 0,07 | 0,01 | 0,03 | 0,05 | 0,16 | 0,06 |
| exonic | HRCT1 | nonframeshift deletion | NM_001039792:c.64_66del:p.22_22del | 9 | 35906348 | 35906350 | CTG | - | 0,00 | 0,00 | 0,16 | 0,14 | 0,09 | 0,00 |
| exonic | C9orf135 | nonsynonymous SNV | NM_001010940:c.G250A:p.D84N | 9 | 72459530 | 72459530 | G | A | 0,23 | 0,37 | 0,27 | 0,14 | 0,17 | 0,25 |
| exonic | SVEP1 | stopgain SNV | NM_153366:c.G6395A:p.W2132X | 9 | 113173596 | 113173596 | C | T | 0,30 | 0,44 | 0,54 | 0,28 | 0,19 | 0,32 |
| exonic | PTBP3 | nonsynonymous SNV | NM_001244897:c.T1481C:p.L494P | 9 | 114982615 | 114982615 | A | G | 0,00 | 0,00 | 0,18 | 0,00 | 0,00 | 0,03 |
| exonic | TTF1 | nonsynonymous SNV | NM_007344:c.A697G:p.T233A | 9 | 135277512 | 135277512 | T | C | 0,00 | 0,00 | 0,08 | 0,02 | 0,04 | 0,16 |
| exonic | TTF1 | nonsynonymous SNV | NM_007344:c.A692T:p.Y231F | 9 | 135277517 | 135277517 | T | A | 0,00 | 0,00 | 0,12 | 0,02 | 0,04 | 0,16 |
| exonic | TTF1 | nonsynonymous SNV | NM_007344:c.G686A:p.R229Q | 9 | 135277523 | 135277523 | C | T | 0,00 | 0,00 | 0,17 | 0,02 | 0,06 | 0,15 |
| exonic | TTF1 | nonsynonymous SNV | NM_007344:c.A682C:p.N228H | 9 | 135277527 | 135277527 | T | G | 0,00 | 0,00 | 0,11 | 0,06 | 0,07 | 0,10 |
| exonic | TTF1 | nonsynonymous SNV | NM_007344:c.G680A:p.S227N | 9 | 135277529 | 135277529 | C | T | 0,00 | 0,00 | 0,13 | 0,05 | 0,09 | 0,13 |
| exonic | GTF3C5 | stopgain SNV | NM_001122823:c.C87A:p.Y29X | 9 | 135906485 | 135906485 | C | A | 0,14 | 0,00 | 0,00 | 0,00 | 0,00 | 0,18 |
| exonic | FAM171A1 | nonsynonymous SNV | NM_001010924:c.G181A:p.A61T | 10 | 15326021 | 15326021 | C | T | 0,20 | 0,39 | 0,40 | 0,24 | 0,26 | 0,34 |
| exonic | KIAA1462 | nonsynonymous SNV | NM_020848:c.C2053T:p.R685W | 10 | 30317024 | 30317024 | G | A | 0,00 | 0,41 | 0,03 | 0,00 | 0,00 | 0,01 |
| exonic | RBP3 | nonsynonymous SNV | NM_002900:c.G2326A:p.A776T | 10 | 48388552 | 48388552 | C | T | 0,33 | 0,42 | 0,29 | 0,39 | 0,08 | 0,38 |
| exonic | ANK3 | nonsynonymous SNV | NM_020987:c.C11915T:p.T3972I | 10 | 61828724 | 61828724 | G | A | 0,36 | 0,52 | 0,52 | 0,15 | 0,31 | 0,26 |
| exonic | GRID1 | nonsynonymous SNV | NM_017551:c.A2848G:p.N950D | 10 | 87362212 | 87362212 | T | C | 0,33 | 0,46 | 0,69 | 0,20 | 0,21 | 0,25 |
| exonic | C10orf32 | nonsynonymous SNV | NM_001136200:c.A53G:p.K18R | 10 | 104614096 | 104614096 | A | G | 0,40 | 0,00 | 0,00 | 0,00 | 0,00 | 0,00 |
| exonic | INA | nonsynonymous SNV | NM_032727:c.G826A:p.A276T | 10 | 105037794 | 105037794 | G | A | 0,00 | 0,50 | 0,00 | 0,00 | 0,00 | 0,00 |
| exonic | CALHM1 | stopgain SNV | NM_001001412:c.G853T:p.E285X | 10 | 105215207 | 105215207 | C | A | 0,00 | 0,00 | 0,14 | 0,00 | 0,00 | 0,33 |
| exonic | SORCS3 | nonsynonymous SNV | NM_014978:c.T3438A:p.F1146L | 10 | 107016677 | 107016677 | T | A | 0,33 | 0,42 | 0,58 | 0,26 | 0,28 | 0,24 |
| exonic | CASP7 | nonsynonymous SNV | NM_001267058:c.C791T:p.P264L | 10 | 115489253 | 115489253 | C | T | 0,44 | 0,48 | 0,52 | 0,23 | 0,14 | 0,21 |
| exonic | FUOM | nonsynonymous SNV | NM_001098483:c.G266T:p.G89V | 10 | 135170202 | 135170202 | C | A | 0,00 | 0,00 | 0,00 | 0,36 | 0,00 | 0,07 |
| exonic | CD151 | nonsynonymous SNV | NM_001039490:c.C674A:p.A225D | 11 | 838000 | 838000 | C | A | 0,00 | 0,44 | 0,00 | 0,00 | 0,00 | 0,00 |
| exonic | STIM1 | stopgain SNV | NM_001277961:c.G451T:p.E151X | 11 | 4076821 | 4076821 | G | T | 0,00 | 0,14 | 0,22 | 0,00 | 0,00 | 0,00 |
| exonic | OR51S1 | nonsynonymous SNV | NM_001004758:c.C497T:p.P166L | 11 | 4869942 | 4869942 | G | A | 0,29 | 0,47 | 0,47 | 0,15 | 0,14 | 0,33 |
| exonic | OR8J3 | nonsynonymous SNV | NM_001004064:c.C8T:p.P3L | 11 | 55905187 | 55905187 | G | A | 0,18 | 0,60 | 0,22 | 0,17 | 0,28 | 0,16 |
| exonic | OR5M9 | nonsynonymous SNV | NM_001004743:c.G858A:p.M286I | 11 | 56230020 | 56230020 | C | T | 0,28 | 0,04 | 0,21 | 0,00 | 0,00 | 0,13 |
| exonic | KDM2A | nonframeshift deletion | NM_001256405:c.1261_1263del:p.421_421del | 11 | 67018079 | 67018081 | GAG | - | 0,00 | 0,06 | 0,06 | 0,00 | 0,17 | 0,00 |
| exonic | PDE2A | nonsynonymous SNV | NM_001143839:c.G2523A:p.M841I | 11 | 72289348 | 72289348 | C | T | 0,32 | 0,13 | 0,20 | 0,00 | 0,00 | 0,02 |
| exonic | FAT3 | nonsynonymous SNV | NM_001008781:c.C1012T:p.P338S | 11 | 92086290 | 92086290 | C | T | 0,23 | 0,09 | 0,20 | 0,00 | 0,00 | 0,11 |
| exonic | MLL | nonsynonymous SNV | NM_001197104:c.A1571T:p.N524I | 11 | 118343445 | 118343445 | A | T | 0,28 | 0,58 | 0,38 | 0,22 | 0,16 | 0,20 |
| exonic | C12orf4 | nonsynonymous SNV | NM_020374:c.T1297C:p.C433R | 12 | 4609447 | 4609447 | A | G | 0,00 | 0,00 | 0,15 | 0,00 | 0,00 | 0,00 |
| exonic | ATN1 | nonsynonymous SNV | NM_001007026:c.T1509G:p.H503Q | 12 | 7045939 | 7045939 | T | G | 0,05 | 0,05 | 0,05 | 0,03 | 0,12 | 0,07 |
| exonic | C1S | nonsynonymous SNV | NM_001734:c.C1348T:p.P450S | 12 | 7177236 | 7177236 | C | T | 0,25 | 0,57 | 0,42 | 0,25 | 0,22 | 0,28 |
| exonic | PRB3 | nonsynonymous SNV | NM_006249:c.G695A:p.G232E | 12 | 11420488 | 11420488 | C | T | 0,10 | 0,00 | 0,14 | 0,00 | 0,00 | 0,00 |
| exonic | SLCO1B7 | stopgain SNV | NM_001009562:c.A313T:p.R105X | 12 | 21174489 | 21174489 | A | T | 0,20 | 0,05 | 0,08 | 0,10 | 0,00 | 0,05 |
| exonic | ABCC9 | nonsynonymous SNV | NM_005691:c.G2114A:p.G705D | 12 | 22025643 | 22025643 | C | T | 0,08 | 0,04 | 0,13 | 0,06 | 0,00 | 0,10 |
| exonic | IPO8 | nonsynonymous SNV | NM_001190995:c.C1624T:p.L542F | 12 | 30802100 | 30802100 | G | A | 0,16 | 0,06 | 0,26 | 0,15 | 0,02 | 0,19 |
| exonic | ACVR1B | nonsynonymous SNV | NM_004302:c.T70G:p.S24A | 12 | 52345597 | 52345597 | T | G | 0,33 | 0,40 | 0,27 | 0,38 | 0,50 | 0,27 |
| exonic | KRT74 | nonsynonymous SNV | NM_175053:c.G1147A:p.E383K | 12 | 52962161 | 52962161 | C | T | 0,00 | 0,14 | 0,27 | 0,12 | 0,07 | 0,17 |
| exonic | KRT4 | nonsynonymous SNV | NM_002272:c.G1418T:p.S473I | 12 | 53200998 | 53200998 | C | A | 0,00 | 0,00 | 0,00 | 0,00 | 0,20 | 0,00 |
| exonic | ESPL1 | nonsynonymous SNV | NM_012291:c.C5461A:p.R1821S | 12 | 53684721 | 53684721 | C | A | 0,44 | 0,00 | 0,00 | 0,00 | 0,00 | 0,00 |
| exonic | ITGA7 | nonsynonymous SNV | NM_001144996:c.G2405A:p.R802Q | 12 | 56088079 | 56088079 | C | T | 0,41 | 0,65 | 0,55 | 0,30 | 0,31 | 0,40 |

| | | | | | | | | | | | | | | |
|---|---|---|---|---|---|---|---|---|---|---|---|---|---|---|
| exonic | GNS | nonsynonymous SNV | NM_002076:c.G1205A:p.G402D | 12 | 65116889 | 65116889 | C | T | 0,28 | 0,68 | 0,63 | 0,38 | 0,31 | 0,50 |
| exonic | TBC1D15 | nonsynonymous SNV | NM_001146213:c.C625T:p.L209F | 12 | 72287072 | 72287072 | C | T | 0,14 | 0,19 | 0,33 | 0,32 | 0,00 | 0,25 |
| exonic | TBC1D15 | nonsynonymous SNV | NM_001146213:c.C749G:p.S250C | 12 | 72288557 | 72288557 | C | G | 0,12 | 0,05 | 0,05 | 0,08 | 0,00 | 0,00 |
| exonic | TBC1D15 | stopgain SNV | NM_001146213:c.C757T:p.Q253X | 12 | 72288565 | 72288565 | C | T | 0,10 | 0,07 | 0,07 | 0,12 | 0,00 | 0,00 |
| exonic | TBC1D15 | nonsynonymous SNV | NM_001146213:c.C802A:p.P268T | 12 | 72288610 | 72288610 | C | A | 0,15 | 0,05 | 0,09 | 0,10 | 0,00 | 0,08 |
| exonic | TMEM116 | nonsynonymous SNV | NM_001193453:c.C73T:p.P25S | 12 | 112441609 | 112441609 | G | A | 0,51 | 0,65 | 0,63 | 0,35 | 0,24 | 0,29 |
| exonic | NOS1 | nonsynonymous SNV | NM_001204213:c.G2823A:p.M941I | 12 | 117660664 | 117660664 | C | T | 0,48 | 0,68 | 0,37 | 0,18 | 0,36 | 0,27 |
| exonic | GJA3 | nonsynonymous SNV | NM_021954:c.G413A:p.R138K | 13 | 20717015 | 20717015 | C | T | 0,27 | 0,47 | 0,48 | 0,15 | 0,29 | 0,50 |
| exonic | FAM194B | nonsynonymous SNV | NM_182542:c.G324T:p.E108D | 13 | 46170817 | 46170817 | C | A | 0,01 | 0,02 | 0,14 | 0,08 | 0,14 | 0,06 |
| exonic | FAM194B | nonsynonymous SNV | NM_182542:c.C302A:p.A101E | 13 | 46170839 | 46170839 | G | T | 0,00 | 0,02 | 0,15 | 0,08 | 0,17 | 0,09 |
| exonic | OR4K1 | nonsynonymous SNV | NM_001004063:c.A848G:p.N283S | 14 | 20404673 | 20404673 | A | G | 0,08 | 0,12 | 0,16 | 0,09 | 0,06 | 0,07 |
| exonic | ZFHX2 | nonsynonymous SNV | NM_033400:c.G7138A:p.G2380R | 14 | 23991752 | 23991752 | C | T | 0,26 | 0,33 | 0,47 | 0,12 | 0,15 | 0,22 |
| exonic | NFATC4 | nonsynonymous SNV | NM_001198965:c.C2123T:p.P708L | 14 | 24845566 | 24845566 | C | T | 0,33 | 0,27 | 0,36 | 0,17 | 0,25 | 0,24 |
| exonic | AP4S1 | nonsynonymous SNV | NM_001254727:c.A370G:p.R124G | 14 | 31552735 | 31552735 | A | G | 0,00 | 0,00 | 0,23 | 0,07 | 0,08 | 0,03 |
| exonic | GPR137C | nonsynonymous SNV | NM_001099652:c.C344T:p.P115L | 14 | 53020209 | 53020209 | C | T | 0,32 | 0,24 | 0,43 | 0,23 | 0,24 | 0,27 |
| exonic | PLEKHD1 | nonsynonymous SNV | NM_001161498:c.C833T:p.P278L | 14 | 69992749 | 69992749 | C | T | 0,00 | 0,22 | 0,30 | 0,26 | 0,23 | 0,19 |
| exonic | PPP4R4 | nonsynonymous SNV | NM_058237:c.G784A:p.D262N | 14 | 94703954 | 94703954 | G | A | 0,26 | 0,29 | 0,29 | 0,22 | 0,21 | 0,17 |
| exonic | FMN1 | nonsynonymous SNV | NM_001103184:c.T2120C:p.L707P | 15 | 33261113 | 33261113 | A | G | 0,40 | 0,06 | 0,00 | 0,20 | 0,14 | 0,09 |
| exonic | AQR | nonsynonymous SNV | NM_014691:c.C3242T:p.P1081L | 15 | 35167061 | 35167061 | G | A | 0,22 | 0,30 | 0,24 | 0,13 | 0,10 | 0,17 |
| exonic | JMJD7,JMJD7-PLA2G4B | nonsynonymous SNV | NM_001114632:c.C208T:p.P70S | 15 | 42127081 | 42127081 | C | T | 0,20 | 0,27 | 0,21 | 0,13 | 0,21 | 0,26 |
| exonic | CASC4 | nonsynonymous SNV | NM_138423:c.C350T:p.S117L | 15 | 44615185 | 44615185 | C | T | 0,27 | 0,42 | 0,19 | 0,11 | 0,15 | 0,18 |
| exonic | B2M | nonsynonymous SNV | NM_004048:c.A1G:p.M1V | 15 | 45003745 | 45003745 | A | G | 0,00 | 0,35 | 0,40 | 0,23 | 0,00 | 0,17 |
| exonic | HERC1 | nonsynonymous SNV | NM_003922:c.C668T:p.S223L | 15 | 64067155 | 64067155 | G | A | 0,21 | 0,27 | 0,42 | 0,14 | 0,01 | 0,18 |
| exonic | IGDCC4 | nonsynonymous SNV | NM_020962:c.C3518T:p.P1173L | 15 | 65676582 | 65676582 | G | A | 0,31 | 0,00 | 0,00 | 0,00 | 0,00 | 0,00 |
| exonic | MAP2K1 | nonframeshift insertion | NM_002755:c.162_163insAGA:p.L54delinsLR | 15 | 66727446 | 66727446 | - | AGA | 0,00 | 0,00 | 0,00 | 0,00 | 0,13 | 0,00 |
| exonic | C15orf40 | nonsynonymous SNV | NM_001160113:c.T481G:p.Y161D | 15 | 83677185 | 83677185 | A | C | 0,00 | 0,09 | 0,38 | 0,07 | 0,00 | 0,08 |
| exonic | ADAMTSL3 | stopgain SNV | NM_207517:c.C3478T:p.Q1160X | 15 | 84651858 | 84651858 | C | T | 0,28 | 0,39 | 0,44 | 0,23 | 0,00 | 0,22 |
| exonic | ALPK3 | nonsynonymous SNV | NM_020778:c.G1733A:p.R578Q | 15 | 85383637 | 85383637 | G | A | 0,35 | 0,35 | 0,40 | 0,00 | 0,00 | 0,32 |
| exonic | ABCA3 | nonsynonymous SNV | NM_001089:c.G175A:p.G59S | 16 | 2376155 | 2376155 | C | T | 0,00 | 0,17 | 0,34 | 0,09 | 0,16 | 0,18 |
| exonic | RBFOX1 | nonsynonymous SNV | NM_145891:c.C1079T:p.A360V | 16 | 7759078 | 7759078 | C | T | 0,20 | 0,35 | 0,35 | 0,08 | 0,19 | 0,16 |
| exonic | SNX29 | nonsynonymous SNV | NM_032167:c.G1510A:p.A504T | 16 | 12223530 | 12223530 | G | A | 0,35 | 0,43 | 0,60 | 0,25 | 0,25 | 0,27 |
| exonic | BFAR | nonsynonymous SNV | NM_016561:c.C793T:p.P265S | 16 | 14755758 | 14755758 | C | T | 0,23 | 0,29 | 0,39 | 0,21 | 0,15 | 0,26 |
| exonic | DNAH3 | nonsynonymous SNV | NM_017539:c.G10913A:p.G3638E | 16 | 20966293 | 20966293 | C | T | 0,24 | 0,36 | 0,30 | 0,13 | 0,18 | 0,22 |
| exonic | DNAH3 | nonsynonymous SNV | NM_017539:c.G10912A:p.G3638R | 16 | 20966294 | 20966294 | C | T | 0,26 | 0,36 | 0,30 | 0,13 | 0,18 | 0,21 |
| exonic | HS3ST2 | nonsynonymous SNV | NM_006043:c.C1015T:p.P339S | 16 | 22926794 | 22926794 | C | T | 0,27 | 0,33 | 0,35 | 0,08 | 0,16 | 0,15 |
| exonic | SCNN1G | nonsynonymous SNV | NM_001039:c.C1690T:p.R564C | 16 | 23226530 | 23226530 | C | T | 0,21 | 0,39 | 0,31 | 0,19 | 0,16 | 0,18 |
| exonic | AQP8 | nonsynonymous SNV | NM_001169:c.G749A:p.G250E | 16 | 25239776 | 25239776 | G | A | 0,15 | 0,35 | 0,39 | 0,11 | 0,36 | 0,15 |
| exonic | APOBR | nonsynonymous SNV | NM_018690:c.G1083C:p.E361D | 16 | 28507445 | 28507445 | G | C | 0,00 | 0,29 | 0,07 | 0,00 | 0,29 | 0,13 |
| exonic | SLC12A3 | nonsynonymous SNV | NM_000339:c.C2516T:p.T839I | 16 | 56926934 | 56926934 | C | T | 0,00 | 0,00 | 0,00 | 0,00 | 0,00 | 0,19 |
| exonic | ELMO3 | nonsynonymous SNV | NM_024712:c.C1840A:p.L614M | 16 | 67237027 | 67237027 | C | A | 0,00 | 0,44 | 0,00 | 0,00 | 0,00 | 0,00 |
| exonic | TCF25 | nonsynonymous SNV | NM_014972:c.A35G:p.E12G | 16 | 89940110 | 89940110 | A | G | 0,15 | 0,12 | 0,05 | 0,12 | 0,32 | 0,06 |
| exonic | C16orf3 | nonsynonymous SNV | NM_001214:c.T131C:p.V44A | 16 | 90095620 | 90095620 | A | G | 0,21 | 0,00 | 0,00 | 0,00 | 0,00 | 0,14 |
| exonic | ZZEF1 | nonsynonymous SNV | NM_015113:c.G8857C:p.A2953P | 17 | 3910213 | 3910213 | C | G | 0,29 | 0,07 | 0,20 | 0,09 | 0,17 | 0,11 |
| exonic | POLR2A | nonsynonymous SNV | NM_000937:c.T5065A:p.S1689T | 17 | 7416648 | 7416648 | T | A | 0,00 | 0,00 | 0,21 | 0,27 | 0,15 | 0,00 |
| exonic | POLR2A | nonsynonymous SNV | NM_000937:c.C5066G:p.S1689C | 17 | 7416649 | 7416649 | C | G | 0,00 | 0,00 | 0,21 | 0,29 | 0,17 | 0,00 |
| exonic | POLR2A | nonsynonymous SNV | NM_000937:c.G5072A:p.S1691N | 17 | 7416655 | 7416655 | G | A | 0,00 | 0,00 | 0,23 | 0,22 | 0,17 | 0,00 |
| exonic | POLR2A | nonsynonymous SNV | NM_000937:c.T5098A:p.S1700T | 17 | 7416681 | 7416681 | T | A | 0,06 | 0,00 | 0,25 | 0,06 | 0,27 | 0,09 |
| exonic | POLR2A | stopgain SNV | NM_000937:c.C5099G:p.S1700X | 17 | 7416682 | 7416682 | C | G | 0,06 | 0,00 | 0,15 | 0,00 | 0,19 | 0,09 |
| exonic | POLR2A | nonsynonymous SNV | NM_000937:c.T5119A:p.S1707T | 17 | 7416702 | 7416702 | T | A | 0,00 | 0,00 | 0,21 | 0,09 | 0,27 | 0,10 |
| exonic | POLR2A | nonsynonymous SNV | NM_000937:c.C5120G:p.S1707W | 17 | 7416703 | 7416703 | C | G | 0,00 | 0,00 | 0,13 | 0,03 | 0,10 | 0,05 |
| exonic | POLR2A | nonsynonymous SNV | NM_000937:c.T5149A:p.S1717T | 17 | 7416732 | 7416732 | T | A | 0,00 | 0,00 | 0,20 | 0,00 | 0,16 | 0,05 |
| exonic | POLR2A | nonsynonymous SNV | NM_000937:c.C5150G:p.S1717C | 17 | 7416733 | 7416733 | C | G | 0,00 | 0,00 | 0,20 | 0,00 | 0,16 | 0,05 |
| exonic | POLR2A | nonsynonymous SNV | NM_000937:c.G5156A:p.S1719N | 17 | 7416739 | 7416739 | G | A | 0,00 | 0,00 | 0,17 | 0,00 | 0,13 | 0,05 |
| exonic | CYB5D1 | nonsynonymous SNV | NM_144607:c.A274T:p.R92W | 17 | 7761960 | 7761960 | A | T | 0,26 | 0,45 | 0,33 | 0,20 | 0,33 | 0,27 |
| exonic | MYH4 | nonsynonymous SNV | NM_017533:c.G5435A:p.G1812E | 17 | 10348324 | 10348324 | C | T | 0,16 | 0,30 | 0,29 | 0,08 | 0,15 | 0,17 |

| | | | | | | | | | | | | | | |
|---|---|---|---|---|---|---|---|---|---|---|---|---|---|---|
| exonic | SHISA6 | nonsynonymous SNV | NM_001173461:c.C1247T:p.P416L | 17 | 11461365 | 11461365 | C | T | 0,13 | 0,50 | 0,44 | 0,43 | 0,17 | 0,25 |
| exonic | KCNH4 | nonsynonymous SNV | NM_012285:c.C869T:p.S290F | 17 | 40327715 | 40327715 | G | A | 0,09 | 0,44 | 0,34 | 0,16 | 0,09 | 0,24 |
| exonic | BCAS3 | nonsynonymous SNV | NM_017679:c.C1901T:p.S634L | 17 | 59115388 | 59115388 | C | T | 0,14 | 0,47 | 0,42 | 0,19 | 0,22 | 0,12 |
| exonic | SDK2 | nonsynonymous SNV | NM_001144952:c.C4511T:p.A1504V | 17 | 71382044 | 71382044 | G | A | 0,00 | 0,36 | 0,00 | 0,00 | 0,00 | 0,00 |
| exonic | FBF1 | nonsynonymous SNV | NM_001080542:c.C2711G:p.A904G | 17 | 73910886 | 73910886 | G | C | 0,20 | 0,75 | 0,20 | 0,43 | 0,09 | 0,20 |
| exonic | SEC14L1 | frameshift insertion | NM_001039573:c.100_101insCGATGTT:p.P34fs | 17 | 75186921 | 75186921 | - | CGATGTT | 0,09 | 0,16 | 0,32 | 0,08 | 0,06 | 0,14 |
| exonic | ENGASE | nonsynonymous SNV | NM_001042573:c.C1541T:p.T514I | 17 | 77080718 | 77080718 | C | T | 0,00 | 0,41 | 0,28 | 0,16 | 0,12 | 0,13 |
| exonic | LRRC30 | nonsynonymous SNV | NM_001105581:c.G64A:p.G22R | 18 | 7231200 | 7231200 | G | A | 0,00 | 0,35 | 0,37 | 0,24 | 0,11 | 0,22 |
| exonic | ANKRD12 | nonsynonymous SNV | NM_001083625:c.A2467C:p.K823Q | 18 | 9255801 | 9255801 | A | C | 0,00 | 0,00 | 0,05 | 0,00 | 0,02 | 0,19 |
| exonic | DSC3 | nonsynonymous SNV | NM_001941:c.G2176A:p.D726N | 18 | 28581643 | 28581643 | C | T | 0,23 | 0,35 | 0,31 | 0,08 | 0,12 | 0,22 |
| exonic | CDH20 | nonsynonymous SNV | NM_031891:c.C353T:p.A118V | 18 | 59166525 | 59166525 | C | T | 0,20 | 0,32 | 0,33 | 0,16 | 0,19 | 0,22 |
| exonic | CDH7 | nonsynonymous SNV | NM_004361:c.G307A:p.D103N | 18 | 63477036 | 63477036 | G | A | 0,25 | 0,30 | 0,23 | 0,26 | 0,07 | 0,24 |
| exonic | NFATC1 | nonsynonymous SNV | NM_006162:c.A1175C:p.H392P | 18 | 77171450 | 77171450 | A | C | 0,21 | 0,44 | 0,18 | 0,40 | 0,14 | 0,39 |
| exonic | APC2 | nonsynonymous SNV | NM_005883:c.G2863A:p.E955K | 19 | 1466163 | 1466163 | G | A | 0,00 | 0,00 | 0,00 | 0,00 | 0,00 | 0,50 |
| exonic | LMNB2 | nonsynonymous SNV | NM_032737:c.G568A:p.G190S | 19 | 2438277 | 2438277 | C | T | 0,00 | 0,62 | 0,40 | 0,15 | 0,44 | 0,15 |
| exonic | STAP2 | nonsynonymous SNV | NM_001013841:c.C902T:p.P301L | 19 | 4325470 | 4325470 | G | A | 0,00 | 0,20 | 0,46 | 0,18 | 0,20 | 0,17 |
| exonic | PLIN4 | nonsynonymous SNV | NM_001080400:c.G2350A:p.A784T | 19 | 4511580 | 4511580 | C | T | 0,18 | 0,11 | 0,13 | 0,12 | 0,15 | 0,08 |
| exonic | KHSRP | nonsynonymous SNV | NM_003685:c.C1649T:p.T550I | 19 | 6415857 | 6415857 | G | A | 0,50 | 0,00 | 0,00 | 0,00 | 0,00 | 0,00 |
| exonic | LRRC8E | nonsynonymous SNV | NM_001268285:c.T1745C:p.L582P | 19 | 7965539 | 7965539 | T | C | 0,20 | 0,35 | 0,12 | 0,19 | 0,08 | 0,35 |
| exonic | MAP2K7 | nonsynonymous SNV | NM_145185:c.C461T:p.S154F | 19 | 7975351 | 7975351 | C | T | 0,23 | 0,25 | 0,43 | 0,17 | 0,31 | 0,28 |
| exonic | MYO1F | nonsynonymous SNV | NM_012335:c.C86T:p.T29I | 19 | 8620598 | 8620598 | G | A | 0,33 | 0,52 | 0,54 | 0,08 | 0,20 | 0,30 |
| exonic | MUC16 | nonsynonymous SNV | NM_024690:c.G19439A:p.G6480E | 19 | 9068007 | 9068007 | C | T | 0,29 | 0,29 | 0,33 | 0,12 | 0,05 | 0,21 |
| exonic | KRI1 | nonsynonymous SNV | NM_023008:c.C668T:p.S223F | 19 | 10671692 | 10671692 | G | A | 0,33 | 0,33 | 0,56 | 0,22 | 0,09 | 0,20 |
| exonic | TECR | nonsynonymous SNV | NM_138501:c.C827A:p.T276N | 19 | 14676583 | 14676583 | C | A | 0,00 | 0,36 | 0,00 | 0,00 | 0,00 | 0,00 |
| exonic | CYP4F12 | nonsynonymous SNV | NM_023944:c.T639G:p.H213Q | 19 | 15793312 | 15793312 | T | G | 0,00 | 0,00 | 0,16 | 0,00 | 0,00 | 0,00 |
| exonic | CPAMD8 | nonsynonymous SNV | NM_015692:c.C4061A:p.A1354D | 19 | 17017869 | 17017869 | G | T | 0,00 | 0,00 | 0,18 | 0,40 | 0,00 | 0,13 |
| exonic | PBX4 | stopgain SNV | NM_025245:c.C1048T:p.Q350X | 19 | 19672911 | 19672911 | G | A | 0,00 | 0,00 | 0,00 | 0,00 | 0,80 | 0,00 |
| exonic | ZNF91 | nonsynonymous SNV | NM_003430:c.G3534C:p.M1178I | 19 | 23542247 | 23542247 | C | G | 0,00 | 0,08 | 0,12 | 0,19 | 0,11 | 0,07 |
| exonic | ATP4A | nonsynonymous SNV | NM_000704:c.G2686A:p.G896R | 19 | 36044004 | 36044004 | C | T | 0,19 | 0,42 | 0,26 | 0,21 | 0,21 | 0,25 |
| exonic | MLL4 | nonsynonymous SNV | NM_014727:c.G6546C:p.E2182D | 19 | 36223996 | 36223996 | G | C | 0,38 | 0,13 | 0,29 | 0,31 | 0,22 | 0,26 |
| exonic | ZNF527 | nonsynonymous SNV | NM_032453:c.C902T:p.P301L | 19 | 37879853 | 37879853 | C | T | 0,10 | 0,03 | 0,02 | 0,11 | 0,02 | 0,01 |
| exonic | WDR87 | nonsynonymous SNV | NM_031951:c.G5092A:p.E1698K | 19 | 38379102 | 38379102 | C | T | 0,27 | 0,23 | 0,40 | 0,14 | 0,07 | 0,19 |
| exonic | RASGRP4 | nonsynonymous SNV | NM_001146206:c.G974A:p.G325E | 19 | 38903451 | 38903451 | C | T | 0,10 | 0,53 | 0,31 | 0,13 | 0,29 | 0,22 |
| exonic | PRX | nonsynonymous SNV | NM_181882:c.C2228T:p.P743L | 19 | 40902031 | 40902031 | G | A | 0,30 | 0,41 | 0,25 | 0,14 | 0,15 | 0,25 |
| exonic | PRX | nonsynonymous SNV | NM_020956:c.T50G:p.V17G | 19 | 40909747 | 40909747 | A | C | 0,40 | 0,00 | 0,33 | 0,14 | 0,29 | 0,36 |
| exonic | TOMM40 | nonsynonymous SNV | NM_001128917:c.C428T:p.P143L | 19 | 45396179 | 45396179 | C | T | 0,27 | 0,46 | 0,28 | 0,13 | 0,11 | 0,21 |
| exonic | GLTSCR2 | nonsynonymous SNV | NM_015710:c.C1289A:p.T430N | 19 | 48259072 | 48259072 | C | A | 0,00 | 0,50 | 0,00 | 0,00 | 0,00 | 0,00 |
| exonic | SPIB | nonsynonymous SNV | NM_001243998:c.C68T:p.T23I | 19 | 50926863 | 50926863 | C | T | 0,00 | 0,30 | 0,27 | 0,00 | 0,20 | 0,23 |
| exonic | FPR1 | nonsynonymous SNV | NM_002029:c.C257T:p.A86V | 19 | 52249991 | 52249991 | G | A | 0,08 | 0,38 | 0,30 | 0,26 | 0,09 | 0,23 |
| exonic | ZNF880 | nonsynonymous SNV | NM_001145434:c.A1133G:p.K378R | 19 | 52887966 | 52887966 | A | G | 0,01 | 0,09 | 0,11 | 0,03 | 0,06 | 0,01 |
| exonic | ZNF534 | nonsynonymous SNV | NM_001143938:c.G392A:p.G131E | 19 | 52941105 | 52941105 | G | A | 0,24 | 0,40 | 0,32 | 0,17 | 0,09 | 0,19 |
| exonic | SGK110 | nonsynonymous SNV | NM_001199824:c.G490A:p.V164I | 19 | 56052802 | 56052802 | C | T | 1,00 | 0,00 | 0,00 | 0,00 | 0,00 | 0,00 |
| exonic | ZSCAN5B | nonsynonymous SNV | NM_001080456:c.C736T:p.P246S | 19 | 56702209 | 56702209 | G | A | 0,24 | 0,26 | 0,39 | 0,19 | 0,07 | 0,21 |
| exonic | ZNF671 | nonsynonymous SNV | NM_024833:c.C1452G:p.C484W | 19 | 58232002 | 58232002 | G | C | 0,00 | 0,00 | 0,03 | 0,00 | 0,13 | 0,01 |
| exonic | ZNF671 | frameshift insertion | NM_024833:c.1449_1450insAT:p.K483fs | 19 | 58232005 | 58232005 | - | AT | 0,00 | 0,00 | 0,04 | 0,00 | 0,16 | 0,03 |
| exonic | ZNF671 | stopgain SNV | NM_024833:c.T1437A:p.Y479X | 19 | 58232017 | 58232017 | A | T | 0,00 | 0,01 | 0,03 | 0,00 | 0,19 | 0,03 |
| exonic | ZNF671 | nonsynonymous SNV | NM_024833:c.C1433T:p.P478L | 19 | 58232021 | 58232021 | G | A | 0,00 | 0,01 | 0,03 | 0,00 | 0,18 | 0,02 |
| exonic | TGM6 | nonsynonymous SNV | NM_001254734:c.G458A:p.R153K | 20 | 2377185 | 2377185 | G | A | 0,18 | 0,28 | 0,32 | 0,24 | 0,12 | 0,24 |
| exonic | CHGB | nonsynonymous SNV | NM_001819:c.C899T:p.P300L | 20 | 5903689 | 5903689 | C | T | 0,31 | 0,33 | 0,37 | 0,33 | 0,26 | 0,10 |
| exonic | HAO1 | nonsynonymous SNV | NM_017545:c.G304A:p.G102R | 20 | 7895052 | 7895052 | C | T | 0,00 | 0,22 | 0,36 | 0,16 | 0,21 | 0,13 |
| exonic | PTPRT | nonsynonymous SNV | NM_007050:c.C1688T:p.S563F | 20 | 40980798 | 40980798 | G | A | 0,14 | 0,29 | 0,17 | 0,20 | 0,06 | 0,16 |
| exonic | PHACTR3 | nonsynonymous SNV | NM_001199505:c.G659A:p.R220K | 20 | 58342367 | 58342367 | G | A | 0,12 | 0,33 | 0,48 | 0,09 | 0,18 | 0,19 |
| exonic | BTG3 | nonsynonymous SNV | NM_001130914:c.C392T:p.A131V | 21 | 18976514 | 18976514 | G | A | 0,00 | 0,06 | 0,19 | 0,12 | 0,09 | 0,07 |
| exonic | BTG3 | nonsynonymous SNV | NM_001130914:c.C345G:p.C115W | 21 | 18976561 | 18976561 | G | C | 0,00 | 0,00 | 0,18 | 0,20 | 0,14 | 0,00 |
| exonic | JAM2 | nonsynonymous SNV | NM_001270408:c.A901G:p.K301E | 21 | 27086579 | 27086579 | A | G | 0,00 | 0,02 | 0,14 | 0,04 | 0,09 | 0,02 |

| | | | | | | | | | | | | | | |
|---|---|---|---|---|---|---|---|---|---|---|---|---|---|---|
| exonic | DYRK1A | nonsynonymous SNV | NM_001396:c.G454A:p.G152R | 21 | 38853066 | 38853066 | G | A | 0,00 | 0,02 | 0,00 | 0,00 | 0,00 | 0,11 |
| exonic | LCA5L | nonsynonymous SNV | NM_152505:c.T2003A:p.I668K | 21 | 40777818 | 40777818 | A | T | 0,00 | 0,00 | 0,24 | 0,00 | 0,00 | 0,00 |
| exonic | SLC19A1 | nonsynonymous SNV | NM_001205206:c.C1349T:p.A450V | 21 | 46935011 | 46935011 | G | A | 0,00 | 0,00 | 0,00 | 0,00 | 0,44 | 0,00 |
| exonic | SLC19A1 | nonframeshift deletion | NM_001205207:c.214_216del:p.72_72del | 21 | 46951916 | 46951918 | CAG | - | 0,00 | 0,26 | 0,00 | 0,00 | 0,15 | 0,00 |
| exonic | IL2RB | nonsynonymous SNV | NM_000878:c.C1391T:p.P464L | 22 | 37524401 | 37524401 | G | A | 0,43 | 0,59 | 0,28 | 0,07 | 0,13 | 0,42 |
| exonic | CACNA1I | nonsynonymous SNV | NM_001003406:c.C4825A:p.L1609M | 22 | 40073421 | 40073421 | C | A | 0,00 | 0,00 | 0,00 | 0,00 | 0,00 | 0,27 |
| exonic | SLC25A17 | nonsynonymous SNV | NM_006358:c.C665T:p.P222L | 22 | 41173072 | 41173072 | G | A | 0,15 | 0,38 | 0,29 | 0,27 | 0,26 | 0,16 |
| exonic | TCF20 | nonframeshift deletion | NM_005650:c.534_536del:p.178_179del | 22 | 42610776 | 42610778 | TGC | - | 0,00 | 0,15 | 0,00 | 0,02 | 0,02 | 0,02 |
| exonic | ARHGAP8,PRR5-ARHGAP8 | nonsynonymous SNV | NM_181335:c.C1207T:p.P403S | 22 | 45258380 | 45258380 | C | T | 0,28 | 0,20 | 0,26 | 0,26 | 0,17 | 0,31 |
| exonic | PKDREJ | stopgain SNV | NM_006071:c.G4742A:p.W1581X | 22 | 46654478 | 46654478 | C | T | 0,21 | 0,00 | 0,00 | 0,00 | 0,00 | 0,00 |
| exonic | ACR | nonsynonymous SNV | NM_001097:c.G394A:p.E132K | 22 | 51178234 | 51178234 | G | A | 0,26 | 0,35 | 0,37 | 0,10 | 0,22 | 0,20 |
| exonic | EFNB1 | nonsynonymous SNV | NM_004429:c.G461A:p.R154H | X | 68059561 | 68059561 | G | A | 0,00 | 0,00 | 0,00 | 0,40 | 0,00 | 0,00 |
| exonic | MED12 | nonsynonymous SNV | NM_005120:c.C5326A:p.P1776T | X | 70356431 | 70356431 | C | A | 0,00 | 0,57 | 0,00 | 0,00 | 0,00 | 0,00 |
| exonic | CYLC1 | nonsynonymous SNV | NM_021118:c.C1139A:p.A380E | X | 83128855 | 83128855 | C | A | 0,00 | 0,00 | 0,04 | 0,00 | 0,06 | 0,27 |
| exonic | COL4A5 | nonsynonymous SNV | NM_000495:c.G4306A:p.G1436S | X | 107930720 | 107930720 | G | A | 0,51 | 0,63 | 0,59 | 0,30 | 0,40 | 0,41 |
| exonic | ATP11C | nonsynonymous SNV | NM_001010986:c.A2417G:p.K806R | X | 138845561 | 138845561 | T | C | 0,00 | 0,00 | 0,00 | 0,00 | 0,17 | 0,00 |
| exonic | MAGEC1 | nonsynonymous SNV | NM_005462:c.C767G:p.T256S | X | 140993957 | 140993957 | C | G | 0,00 | 0,10 | 0,11 | 0,16 | 0,07 | 0,12 |
| exonic | MAGEC1 | nonsynonymous SNV | NM_005462:c.T774A:p.S258R | X | 140993964 | 140993964 | T | A | 0,00 | 0,11 | 0,12 | 0,20 | 0,09 | 0,13 |
| exonic | MAGEC1 | nonsynonymous SNV | NM_005462:c.T779C:p.F260S | X | 140993969 | 140993969 | T | C | 0,00 | 0,13 | 0,14 | 0,22 | 0,10 | 0,16 |
| exonic | MAGEC1 | nonsynonymous SNV | NM_005462:c.C820T:p.P274S | X | 140994010 | 140994010 | C | T | 0,00 | 0,12 | 0,30 | 0,19 | 0,20 | 0,14 |
| exonic | SLITRK4 | nonsynonymous SNV | NM_001184749:c.A458G:p.K153R | X | 142718467 | 142718467 | T | C | 0,49 | 0,65 | 0,72 | 0,30 | 0,31 | 0,46 |
| exonic | PLXNB3 | nonsynonymous SNV | NM_005393:c.G295A:p.V99M | X | 153032577 | 153032577 | G | A | 0,00 | 0,22 | 0,00 | 0,33 | 0,00 | 0,00 |
