# Supplementary material for: Intra- and inter-tumor heterogeneity in a vemurafenib-resistant melanoma patient and derived xenografts
Source: EMBO Mol Med. 2015 Jun 23;7(9):1104–18. doi: 10.15252/emmm.201404914 (PMC4568946; doi:10.15252/emmm.201404914)
Supplement: Supplementary file 2 [file emmm0007-1104-sd2.pdf]

EMBO Molecular Medicine

Intra- and inter-tumor heterogeneity in a vemurafenib-resistant melanoma patient and derived xenografts

Kristel Kemper, Oscar Krijgsman, Paulien Cornelissen-Steijger, Aida Shahrabi, Fleur Weeber, Ji-Ying Song, Thomas Kuilman, Daniel J. Vis, Lodewyk F. Wessels, Emile E. Voest, Ton N.M. Schumacher, Christian U. Blank, David J. Adams, John B. Haanen and Daniel S. Peeper

Corresponding author: Daniel Peeper, Netherlands Cancer Institute -Member

Review timeline:

Submission date:	04 December 2014
Editorial Decision:	07 January 2015
Revision received:	28 April 2015
Editorial Decision:	12 May 2015
Revision received:	13 May 2015
Accepted:	22 May 2015

Transaction Report:

(Note: With the exception of the correction of typographical or spelling errors that could be a source of ambiguity, letters and reports are not edited. The original formatting of letters and referee reports may not be reflected in this compilation.)

Editor: Roberto Buccione

1st Editorial Decision 07 January 2015

Thank you for the submission of your manuscript to EMBO Molecular Medicine. We have now heard back from the three Reviewers whom we asked to evaluate your manuscript. We are sorry that it has taken longer than usual to get back to you on your manuscript due to the partial overlap with the Holiday season.

As you will see, the three Reviewers in aggregate find merits in your manuscript but raise two main issues. I will not dwell into much detail, as the Reviewers' comments are clear. I would like, however, to highlight a few main points.

Reviewer 1 is very positive and raises a few interesting questions concerning general relevance that should be directly addressed.

Reviewer 2 while recognising the clinical implications of your study, does essentially question the novelty of the findings.

Reviewer 3 is more reserved and argues that an important weakness of the study is that although it has the potential for far-reaching conclusions, it is based only on one patient. The Reviewer feels that to confer general relevance and more interest to the manuscript, some basic mechanistic issues need to be investigated.

In conclusion, while publication of the paper cannot be considered at this stage, given the potential interest of your findings and the fact that the Reviewers, although critical, were globally positive, we

have decided to give you the opportunity to address the above concerns. While we agree with both Reviewers 2 and 3, if you can provide additional mechanistic insight to complement your observations, the "novelty" aspect would not be taken into account in our final decision. We are thus prepared to consider a substantially revised submission, with the understanding that the Reviewers' concerns must be addressed as mentioned and that acceptance of the manuscript will entail a second round of review.

Please note that it is EMBO Molecular Medicine policy to allow a single round of revision only and that, therefore, acceptance or rejection of the manuscript will depend on the completeness of your responses included in the next, final version of the manuscript.

As you know, EMBO Molecular Medicine has a "scooping protection" policy, whereby similar findings that are published by others during review or revision are not a criterion for rejection. However, I do ask you to get in touch with us after three months if you have not completed your revision, to update us on the status. Please also contact us as soon as possible if similar work is published elsewhere.

All EMBO Press journals now require a complete author checklist (http://embomolmed.embopress.org/authorguide#editorial3) to be submitted with all revised manuscripts. Provision of the author checklist is mandatory at revision stage; The checklist is designed to enhance and standardize reporting of key information in research papers and to support reanalysis and repetition of experiments by the community. The list covers key information for figure panels and captions and focuses on statistics, the reporting of reagents, animal models and human subject-derived data, as well as guidance to optimise data accessibility.

I look forward to seeing a revised form of your manuscript in due time.

***** Reviewer's comments *****

Referee #1 (Remarks):

This manuscript presents a series of novel and interesting findings that are relevant to our understanding of tumour heterogeneity in targeted therapy resistance. The authors also assess the suitability of PDX analyses and highlight that tumour heterogeneity can cause discrepancies using this approach.
This well presented manuscript describes the identification of yet more novel mechanisms of resistance (aberrant form of mutant BRAF and an exon2 insertion in the MEK1 gene leading to hyperactivation) emphasizes the complications linked to targeted therapy resistance. Moreover, this manuscript demonstrates effectively how tumour heterogeneity adds another level of complexity to the situation.
The MEKT55_Q56insN mutation is present in M032R4 (Figure 4C), the same sample that did not display any BRAF amplification. In this set of samples M032R1 did not display a MEK insertion, but a BRAF amplification. The PDX equivalent M302R1.X1 of this metastasis (M032R1) possessed a MEK insertion, but no BRAF amplification (figure 5C and D). This suggests that these mutations could exist independently in different sub-populations within a heterogeneous tumour. The authors also mention that they expect the insertion to create the constitutive activation of MEK (which would explain its activity in 293T cells. It would be important to test whether the MEK insertion mutant can 1. also activate ERK in WT BRAF/WT RAS melanoma cells and 2. provide resistance to BRAF inhibitor (but not MEK inhibitor) in these cells.
This might also shed more light on the 'stable disease' situation in M032R1, which is very striking.

The high level of ERK phosphorylation in all the resistant tumours is impressive, but I am puzzled by the relatively low level of pERK in the 'pre' sample (see Figure 2A and 3A). The tumour was positive for a BRAFV600E mutation, but this seems to not have been functional? Is there any material available to see whether there was an even lower pERK level on treatment? Do the authors have made any comment that I might have missed?
The MEK insertion leads to resistance to BRAFi but to trametinib, but the authors do not explain why this might be the case. They should discuss whether they expect all MEKi to inhibit this MEK mutant or whether different inhibitors are expected to have different effects.

Minor comments:
The indicted heterogeneity of BRAFV600E expression in Figure 2D is difficult to see in these images. A better higher magnification example area should be shown.
Similarly the CT-scan images shown in Figure 1B would improve by showing a magnification of the relevant area.

Referee #2 (Remarks):

In this study, Kemper and colleagues present an in-depth analysis of the resistance mechanisms observed in 5 vemurafenid-resistant metastases of a single patient using IHC, WES and by generating and characterizing PDX of these lesions. They show that (i) reactivation of the MAPK was observed in all vemurafenib-resistant lesions. (ii) In addition to previously identified resistance mechanisms (amplification of mutant BRAF) the authors identified an aberrant form of BRAF, which is likely to be the product of a BRAF fusion gene, and a -new- 3bp in-frame insertion in the MEK1 gene. Interestingly, (iii) this mutation could be identified in a small fraction of the pre-treatment lesion. (iV) Analysis of PDX from the vemurafenid-resistant metastases revealed that multiple resistance mechanisms were present within one metastasis.

This is a very carefully designed and well-conducted study. The manuscript is well-written. The results are clear and well-documented. I have no specific comments or criticisms to formulate on the design of the experiments nor on the data presented.

The study has numerous and important clinical implications; it suffers, however, (to some extend) from lack of novelty. The study focuses on mechanisms of resistance to Vemurafenid alone. Inhibition of the MAPK using a single BRAFV600E-inhibitor is no longer the standard of care in the clinic. Patients with BRAFV600E receive a combination of BRAFV600E-inhibitor and MEK-inhibitor (i.e. dabrafebnid and trametinib). The observation that different resistance mechanisms within a patient or even a single melanoma lesion is important but not novel (see Shi et al., 2014 Cancer Discovery). The concept that resistance-conferring mutations are present in tumours prior to therapy is equally important but again not novel per se (see for example Diaz et al., 2012 Nature). Based on their analyses the authors also highlight an important limitation of PDX as a model system since such models do not necessarily harbour the full genetic heterogeneity observed in the patients. Although such a conclusion is relatively expected/obvious this study nicely illustrates this point.

Referee #3 (Remarks):

Kemper et al., report an interesting and well-performed study demonstrating the severity of melanoma metastatic heterogeneity following acquired resistance to vemurafenib, caused by re-activation of MAPK pathway, in an individual patient. By performing whole exome sequencing on five resistant metastases, the authors show that vemurafenib resistance may entail different mechanisms, which can occur independently in each metastasis. Interestingly, besides the known aberrations in oncogenic BRAF, the authors identify a new MEK1 mutation that confers resistance to vemurafenib. This mutation was found to be pre-existing in a fraction of pretreated tumors suggesting its selection before treatment. The authors then go on and establish patient derived xenografts (PDX) from these vemurafenib resistant metastases and show high degree of inter and intratumoral heterogeneity in the patient samples and corresponding PDX. The authors also conclude that the PDX may not completely model complex genetic heterogeneity of the patient's melanoma, an important conclusion considering the recent widespread usage of PDX as proxy of therapy responses and to guide real-time treatment decisions in melanoma patients. Overall this study reports an interesting observation underscoring how the genetic complexity of vemurafenib resistance correlates with a high degree of intratumoral heterogeneity and reveal a novel MEK1

mutation possibly implicated in drug resistance. However, while it is assumable that this is the case for other patients as well, the overall impact of this study is unfortunately limited to one patient and the derived PDX and at the mechanistic levels the study remains often rather vague.

Specific Comments:
The IHCs of Fig. 1 A, Fig. 2D and the WB of Fig. 3 do not completely match. Especially when looking at the p-ERK IHC staining of R5 and the related WB where p-ERK in this metastasis is among the lowest. Is this due to heterogeneity within individual patient's samples? Please comment on this and increase the number of analysed samples, when required.

Despite their constitutively heightened MAPK pathway melanoma cells with MEKT55_T56insN mutation show reduced colony formation as compared to their MEK1WT or GFP expressing cells in untreated conditions. What is the cause of this slower proliferation or increased basal cell death in the absence of MAPK inhibition? And how does the presence of BRAF and ERK inhibitors then confer a proliferative advantage to this melanoma cells? which pathways, other than downstream ERK signalling, are important for growth stimulation following BRAF and ERK inhibition? The authors should expand the results of Fig. 4, to test the effects of incorporating this mutation on invasion, tumor growth/metastatic potential in vivo as compared to oncogenic BRAFV600E-expressing melanoma cell line.

Related to the question above, unlike M032R5 (Figure 1c), M032R1 demonstrates disease stabilization and when transferred to a PDX model becomes positive for MEKT55_T56insN as the original sample from M032R4. Although not fully capturing the genetic heterogeneity, one wonders why besides histology and MAPK activation status also the tumor growth and (combined) drug treatment patterns in these PDX models, has not been analysed and correlated to the histological and genetic findings?

The authors show that a novel mutation in MEK, that carries with it resistance to vemurafenib, maybe preexisting in a subset of pretreated tumors and confer resistance to MAPK inhibition. However, the high degree of variability observed within the metastasis of the single patient described in this article, raises the question of how important is this mutation for overall vemurafenib mediated resistance? What is the frequency of this mutation in human melanoma and how does it correlate with patient progression and therapy outcome?

Minor points:
Fig. 3A, showing the WB of the MAPK and AKT activation status of pre-treated and vemurafenib resistant metastasis, supports the conclusion that most of resistant metastasis show re-activation of the MAPK pathway, but it also indicates that this is not the case for all. As authors point out later a notable example is M032R4, which displays a much reduced level of p-MEK and p-ERK and no increase in p-AKT or expression of BRAFV600E. This should be highlighted before and the sentence "All resistant metastases showed reactivation of the MAPK pathway, determined by increased expression levels of both p-ERK and p-MEK" in page 8, should be corrected accordingly. Also the sentence 'After discovering that BRAF amplifications and an aberrant form of BRAF were responsible for inducing resistance in four of the five resistant metastases..." should be changed, in fact although very likely, the authors did not discover that these amplifications are responsible for inducing resistance in the metastasis, but show a correlation between vemuranefib resistance and BRAF amplifications at the transcripts and protein levels.
Figure 3a requires total AKT.
Figure 3b requires loading control and please indicate the BRAF mw in the lower panel as well (90 kDa).
Authors suggest that in PDX models the pERK status of patient material is re-established but it is more that the patient material has kept its heightened pERK.

1st Revision - authors' response 28 April 2015

Referee #1

We thank the referee for his/her helpful suggestions to improve the manuscript.

1. *The MEKT55delinsRT mutation is present in M032R4 (Figure 4C), the same sample that did not display any BRAF amplification. In this set of samples M032R1 did not display a MEK insertion, but a BRAF amplification. The PDX equivalent M302R1.X1 of this metastasis (M032R1) possessed a MEK insertion, but no BRAF amplification (previous submission figure 5C and D; new submission Figure 7C and D). This suggests that these mutations could exist independently in different sub-populations within a heterogeneous tumour. The authors also mention that they expect the insertion to create the constitutive activation of MEK (which would explain its activity in 293T cells. It would be important to test whether the MEK insertion mutant can 1. also activate ERK in WT BRAF/WT RAS melanoma cells and 2. provide resistance to BRAF inhibitor (but not MEK inhibitor) in these cells. This might also shed more light on the 'stable disease' situation in M032R1, which is very striking.*

- We thank the referee for this comment and realize that we have apparently not explained this sufficiently clearly in the first version of the manuscript. First, the metastases studied here all are $BRAF^{V600E}$ mutated (which was confirmed both by sequencing and Western Blotting; Figure 3); the $MEK1^{T55delinsRT}$ was acquired on top of the $BRAF^{V600E}$ mutation. The 293T experiment (shown in Figure 4D) indeed shows that the presence of the $BRAF^{V600E}$ mutation is not required to get hyperactivation of the MAPK-pathway by the $MEK1^{T55delinsRT}$, but we now also infected $BRAF^{WT}NRAS^{WT}$ melanoma cells with retrovirus encoding GFP, $MEK1^{WT}$ or $MEK1^{T55delinsRT}$. Also in these cells, $MEK1^{T55delinsRT}$ activated ERK (Fig. I) $BRAF^{WT}NRAS^{WT}$ melanoma cells however do not harbor the $BRAF^{V600E}$ mutation, which is targeted by vemurafenib and dabrafenib, and are therefore unresponsive to these inhibitors.

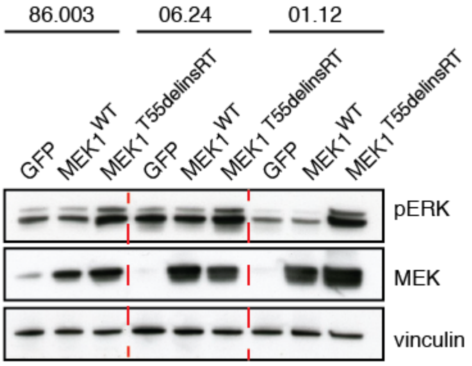

Figure I. Expression of GFP, $MEK1^{WT}$ or $MEK1^{T55delinsRT}$ in three different $BRAF^{WT}NRAS^{WT}$ cell lines.

2. *The high level of ERK phosphorylation in all the resistant tumours is impressive, but I am puzzled by the relatively low level of pERK in the 'pre' sample (see Figure 2A and 3A). The tumour was positive for a BRAFV600E mutation, but this seems to not have been functional?*

- We thank the referee for raising this important point and have provided a control for the pERK staining: we have included normal skin in the new Figure E1. This shows that in M032, the pre-treatment sample, there is already activation of the MAPK-pathway compared to normal skin, which has almost no p-ERK staining; this is now described in the Result section (p6)

3. *Is there any material available to see whether there was an even lower pERK level on treatment? Do the authors have made any comment that I might have missed?*

- Unfortunately, we did not obtain any biopsy from this patient while on treatment and we were therefore unable to check this. However, previously it has been shown by Trunzer et al. (JCO, 2013) that p-ERK levels are indeed reduced in tumor biopsies of melanoma patients, after 15 days of BRAF inhibitor treatment.

4. *The MEK insertion leads to resistance to BRAFi but not to trametinib, but the authors do not explain why this might be the case. They should discuss whether they expect all MEKi to inhibit this MEK mutant or whether different inhibitors are expected to have different effects.*

- We have performed additional colony formation experiments with other MEK inhibitors, i.e., U0126, PD-03259901 and selumetinib (new Figure E6). We found that $MEK1^{T55delinsRT}$ causes broad resistance to these MEK inhibitors. All these inhibitors (including trametinib) have the same mode of action (allosteric inhibitors, non-competitive with ATP), but they have probably different potencies. We have included this figure in the Expanded View (new Figure E6) and discussed this data in the Results (p11) and Discussion (p16-17) section.

5. *The indicted heterogeneity of BRAFV600E expression in Figure 2D is difficult to see in these images. A better higher magnification example area should be shown.*

- The referee is correct: Figure 2D does not show the indicated heterogeneity. We showed the heterogeneity of $BRAF^{V600E}$ expression in Figure E7 (in the previous submission Suppl. Figure 4), which includes multiple pictures and magnifications.

6. *Similarly the CT-scan images shown in Figure 1B would improve by showing a magnification of the relevant area.*

- We have provided the requested magnifications in Figure 1B.

Referee #2:

We would like to thank referee #2 for his/her compliments on our manuscript. Referee #2 did not request any additional experiments.

Referee #3:

We thank the referee for his/her helpful suggestions to improve the manuscript.

1. *The IHCs of Fig. 1A, Fig. 2D and the WB of Fig. 3 do not completely match. Especially when looking at the p-ERK IHC staining of R5 and the related WB where p-ERK in this metastasis is among the lowest. Is this due to heterogeneity within individual patient's samples? Please comment on this and increase the number of analysed samples, when required.*

- The referee is correct: this difference is most likely caused by heterogeneity of the original tumor sample. A different piece of the tumor was used for IHC analysis than the one that was lysed for WB analysis, creating sample bias; this cannot be avoided. This tumor heterogeneity is described in more detail in the Results section “Incomplete capture of tumor heterogeneity in patient-derived xenografts” (p12-13)

2. *Despite their constitutively heightened MAPK pathway melanoma cells with MEKT55_T56insN mutation show reduced colony formation as compared to their MEK1WT or GFP expressing cells in untreated conditions. What is the cause of this slower proliferation or increased basal cell death in the absence of MAPK inhibition? And how does the presence of BRAF and ERK inhibitors then confer a proliferative advantage to this melanoma cells? Which pathways, other than downstream ERK signalling, are*

important for growth stimulation following BRAF and ERK inhibition?

- Again, the referee is correct. As previously shown by Sun et al (Nature 2014) and Moriceau et al. (Cancer Cell, 2015), hyperactivation of the MAPK pathway, e.g. by overexpression of EGFR or ultra-amplification of $BRAF^{V600E}$, reduces cell growth and in fact can be detrimental to the cells. But upon treatment by BRAFi or ERKi, p-ERK levels are reduced to the levels seen in untreated cells without such overexpression or amplification, allowing them to proliferate and survive. In our initial submission we already showed, consistent with the above-mentioned papers, that even low amounts of BRAF, MEK or ERK inhibitors all rescue the cytostatic activity of hyperactive MAPK signaling, allowing for increased proliferation (Figure 4G, E3C, E4C, previous submission Figure 4G, Suppl. Figure 2C and 3C). In response to the referee's suggestion, we have now also performed WB analysis on $MEK1^{T55delinsRT}$ and $MEK1^{WT}$ cells treated with the same concentrations of BRAFi or ERKi as indicated in the colony formations (new Figure E3E-F, E4E-F and E5) for 24h. We found, indeed, that the rescue of cytostatic activity in colony formation by inhibition of BRAF or ERK coincided with normalization of p-ERK and/or p-RSK levels of $MEK1^{T55delinsRT}$ cells relative to those seen in $MEK1^{WT}$ cells (new Figure E3E-F, E4E-F and E5). In conclusion, we observe that moderate inhibition of MAPK signaling is sufficient to enhance proliferation.

3. *The authors should expand the results of Fig. 4, to test the effects of incorporating this mutation on invasion, tumor growth/metastatic potential in vivo as compared to oncogenic BRAFV600E-expressing melanoma cell line.*

- The patient data suggested that there is not a strong role for $MEK1^{T55delinsRT}$ in altering primary tumor characteristics, as the original untreated tumor had only a small fraction of tumor cells carrying this mutation. There is, however, a selective role in the context of drug exposure. In response to the referee's request, we have performed an *in vivo* experiment (new Figure 5) using A375 melanoma cells expressing GFP, $MEK1^{WT}$ or $MEK1^{T55delinsRT}$, treated with the BRAF inhibitor dabrafenib and found that $MEK1^{T55delinsRT}$ indeed induced resistance to this compound *in vivo* (new Figure 5).

  Although no distant metastasis was observed in these mice, we did find signs of local invasion in the tumor margins, specifically in the $MEK1^{T55delinsRT}$ tumors (new Figure 5D). These results indicate that the $MEK1^{T55delinsRT}$ tumor cells are more invasive than their $MEK1^{WT}$ counterparts. We have included this new data as a main figure in the manuscript (new Figure 5D).

4. *Related to the question above, unlike M032R5 (Figure 1c), M032R1 demonstrates disease stabilization and when transferred to a PDX model becomes positive for MEKT55delinsRT as the original sample from M032R4. Although not fully capturing the genetic heterogeneity, one wonders why besides histology and MAPK activation status also the tumor growth and (combined) drug treatment patterns in these PDX models, has not been analyzed and correlated to the histological and genetic findings?*

- In this manuscript, the PDX were merely used to further illustrate the heterogeneity of the tumors from this patient and "issue a warning" about the limitations for using a PDX model as a proxy for the complete human tumor. We are currently performing a comprehensive genetic and histological analysis of a large melanoma PDX library and will share that with the community at a later time.

5. *The authors show that a novel mutation in MEK, that carries with it resistance to vemurafenib, maybe preexisting in a subset of pretreated tumors and confer resistance to MAPK inhibition. However, the high degree of variability observed within the metastasis of the single patient described in this article, raises the question of how important is this mutation for overall vemurafenib mediated resistance? What is the frequency of this mutation in human melanoma and how does it correlate with patient progression and therapy outcome?*

- We have not found this mutation in other sequencing data (e.g. TCGA database and

sequencing data of a large panel of low-passage melanoma cell lines acquired in our lab). However, insertions (like MEK1^{T55delinsRT}) as well as small deletions are notoriously hard to detect and are often missed. Other MEK1 mutations have been identified, some of which are likely to have a similar effect. We have discussed these and their effect on therapy response in the Discussion section of the manuscript (p17).

6. *Fig. 3A, showing the WB of the MAPK and AKT activation status of pre-treated and vemurafenib resistant metastasis, supports the conclusion that most of resistant metastasis show re-activation of the MAPK pathway, but it also indicates that this is not the case for all. As authors point out later a notable example is M032R4, which displays a much reduced level of p-MEK and p-ERK and no increase in p-AKT or expression of BRAFV600E. This should be highlighted before and the sentence "All resistant metastases showed reactivation of the MAPK pathway, determined by increased expression levels of both p-ERK and p-MEK" in page 8, should be corrected accordingly.*

- We thank the referee for pointing this out. We have changed the text accordingly to "Most resistant metastases showed reactivation of the MAPK pathway, as determined by increased expression levels of both p-ERK and p-MEK, although variation was observed among different metastases" (p8).

7. *Also the sentence 'After discovering that BRAF amplifications and an aberrant form of BRAF were responsible for inducing resistance in four of the five resistant metastases..." should be changed, in fact although very likely, the authors did not discover that these amplifications are responsible for inducing resistance in the metastasis, but show a correlation between vemurafenib resistance and BRAF amplifications at the transcripts and protein levels.*

- Again, the referee is correct. We have changed the text accordingly to "After discovering *BRAF* amplifications and an aberrant form of BRAF in four of the five resistant metastases, likely responsible for the vemurafenib resistance, the resistance observed in one metastasis (M032R4) was still unexplained" (p9).

8. *Figure 3a requires total AKT. Figure 3b requires loading control and please indicate the BRAF mw in the lower panel as well (90 kDa).*

- We have provided the requested blots and the indication of the BRAF MW in Figure 3.

9. *Authors suggest that in PDX models the pERK status of patient material is re-established but it is more that the patient material has kept its heightened pERK.*

- We agree with this comment and have changed the text in the figure legend of Figure 7 (previous submission Figure 5) accordingly to "Stainings showed that p-ERK is higher in the PDX derived from the resistant metastases" (p37).

2nd Editorial Decision 12 May 2015

Thank you for the submission of your revised manuscript to EMBO Molecular Medicine. We have now received the enclosed reports from the referees that were asked to re-assess it. As you will see the reviewers are now globally supportive and I am pleased to inform you that we will be able to accept your manuscript pending the following final amendments:

1) As per our Author Guidelines, the description of all reported data that includes statistical testing must state the name of the statistical test used to generate error bars and P values, the number (n) of independent experiments underlying each data point (not replicate measures of one sample), and the actual P value for each test (not merely 'significant' or 'P < 0.05'). I note that for Figure 5B in particular you have not referred to P values at all. Also, you do not appear to have specified in your

manuscript the statistical tests used.

2) Please provide separate files for each figure.

3) We are now encouraging the publication of source data, particularly for electrophoretic gels and blots, with the aim of making primary data more accessible and transparent to the reader. Would you be willing to provide a PDF file per figure that contains the original, uncropped and unprocessed scans of all or at least the key gels used in the manuscript? The PDF files should be labeled with the appropriate figure/panel number, and should have molecular weight markers; further annotation may be useful but is not essential. The PDF files will be published online with the article as supplementary "Source Data" files. If you have any questions regarding this just contact me.

4) Every published paper now includes a 'Synopsis' to further enhance discoverability. Synopses are displayed on the journal webpage and are freely accessible to all readers. They include a short standfirst as well as 2-5 one sentence bullet points that summarise the paper. Please provide the synopsis including the short list of bullet points that summarise the key NEW findings. The bullet points should be designed to be complementary to the abstract - i.e. not repeat the same text. We encourage inclusion of key acronyms and quantitative information. Please use the passive voice. Please attach this information in a separate file or send them by email, we will incorporate it accordingly. You are also welcome to suggest a striking image or visual abstract to illustrate your article. If you do please provide a jpeg file 550 px-wide x 400-px high.

I look forward to receiving the next, final version of your manuscript as soon as possible.

***** Reviewer's comments *****

Referee #1 (Remarks):

All the points that were raised have been addressed. Congratulations on a well performed study.

Referee #2 (Remarks):

This manuscript is of high quality and relevance. Although there may be some concerns about novelty this carefully-conducted study is likely to be well-received and cited in the field.

Referee #3 (Comments on Novelty/Model System):

The manuscript has been improved and the authors have addressed the remaining issues and rendered a more convincing and clear study.

Referee #3 (Remarks):

The manuscript has been improved by the authors who have addressed the remaining issues by adding further experimental evidence that support the main conclusions.

2nd Revision - authors' response 13 May 2015

We are very happy that you have offered to publish our manuscript entitled ‘Intra- and inter-tumor heterogeneity in a vemurafenib-resistant melanoma patient and derived xenografts’ by Kemper et al.

in EMBO Molecular Medicine. We would like to thank you and the reviewers again for the constructive comments during the review process.

We have revised our manuscript according to your requirements. Briefly, we have added the P-values in Figure 5B and added the used statistical test in the Figure Legends. Changes in the manuscript are visualized by track changes. In addition, we have provided the separate Figure Files and Source data for the main figures and included a synopsis.
