# Supplementary figures and images for: Intra- and inter-tumor heterogeneity in a vemurafenib-resistant melanoma patient and derived xenografts
Source: EMBO Mol Med. 2015 Jun 23;7(9):1104–18. doi: 10.15252/emmm.201404914 (PMC4568946; doi:10.15252/emmm.201404914)

Source data for Figure 3

A.

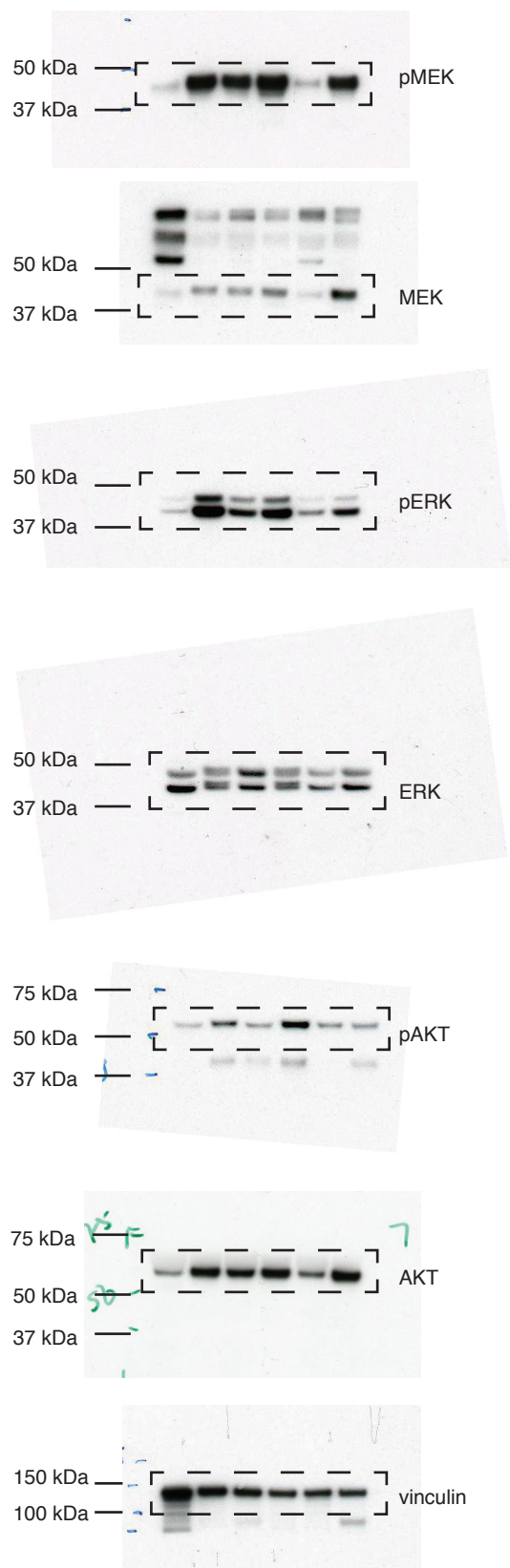

B.

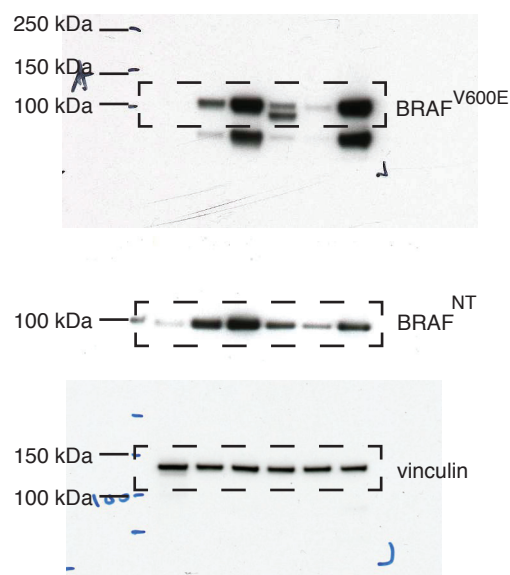

Supplement: Supplementary file 3 [file emmm0007-1104-sd3.pdf]

Source data for Figure 4

C.

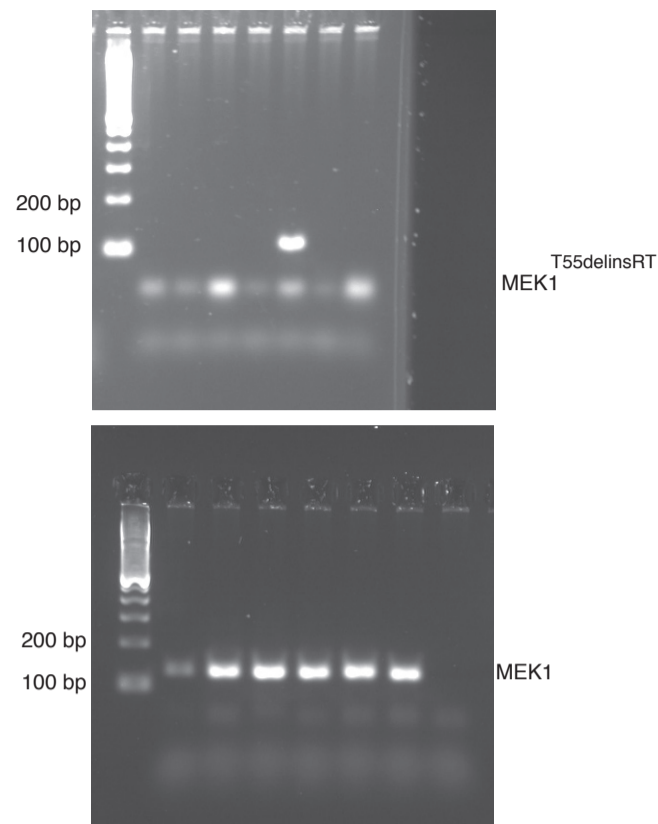

D.

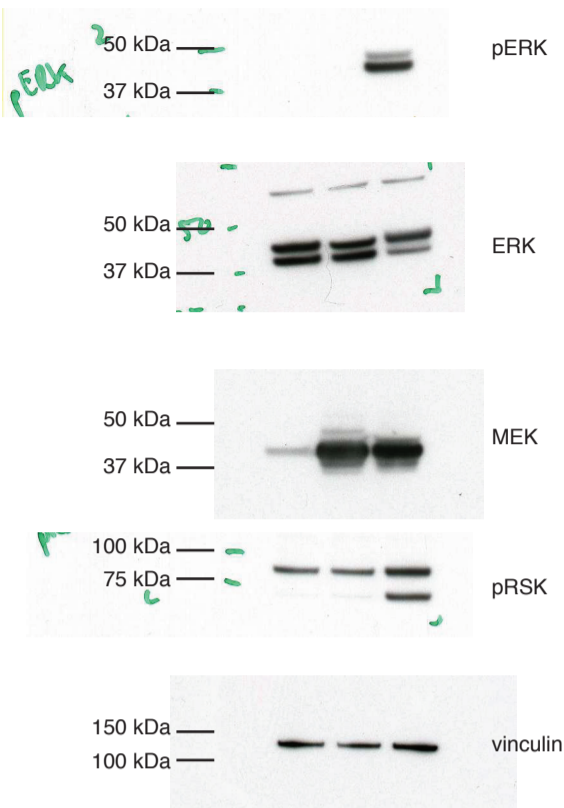

E.

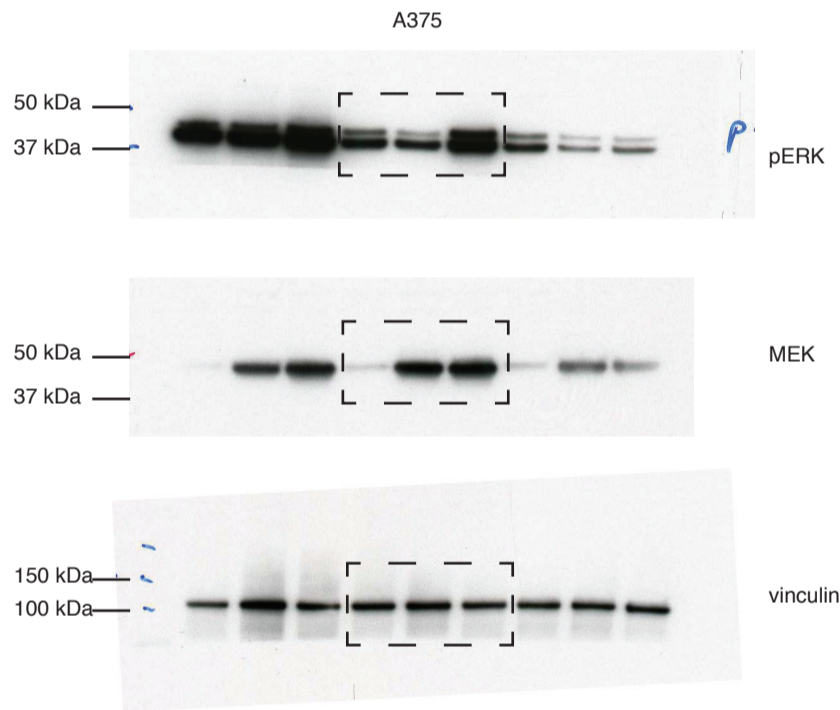

Supplement: Supplementary file 4 [file emmm0007-1104-sd4.pdf]

Source data for Figure 5

C.

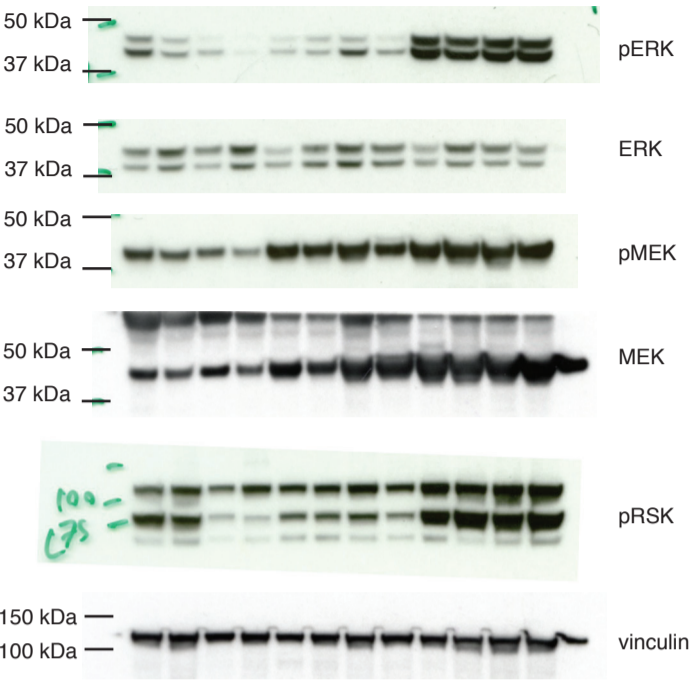

Supplement: Supplementary file 5 [file emmm0007-1104-sd5.pdf]

Source data for Figure 6

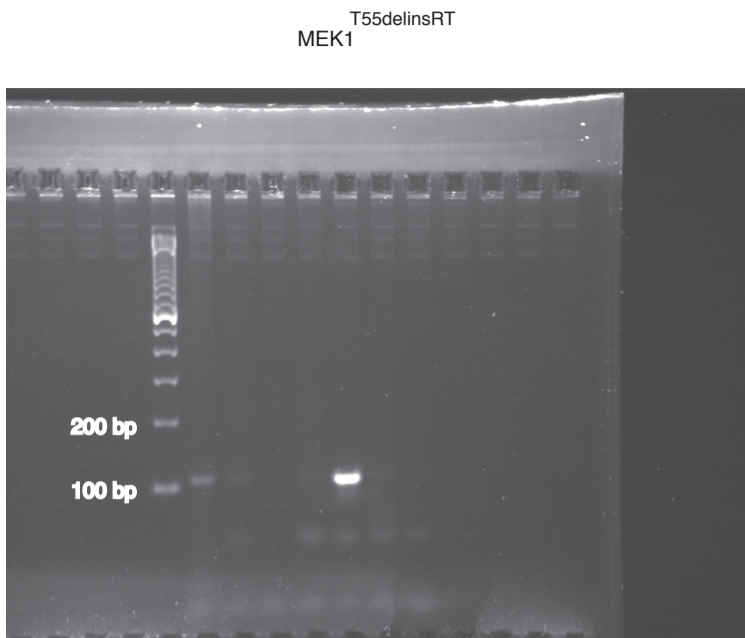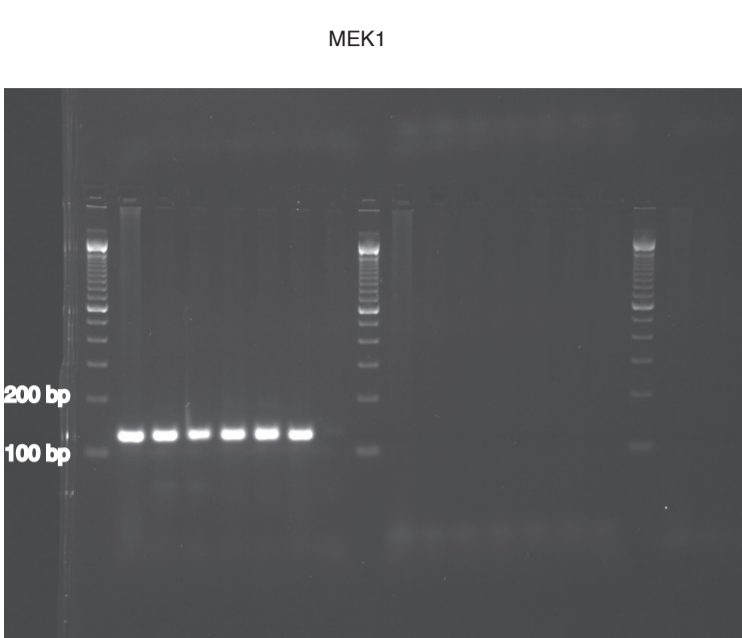

Supplement: Supplementary file 6 [file emmm0007-1104-sd6.pdf]

Source data for Figure 7

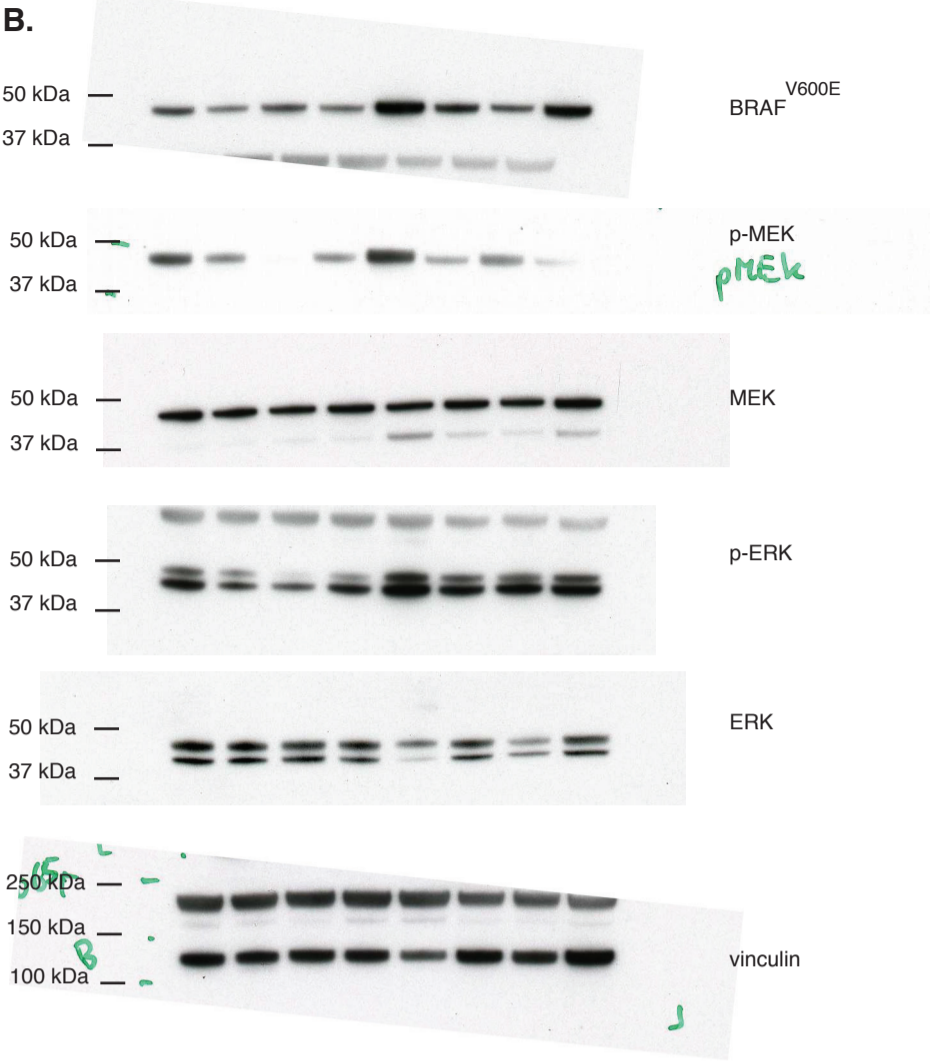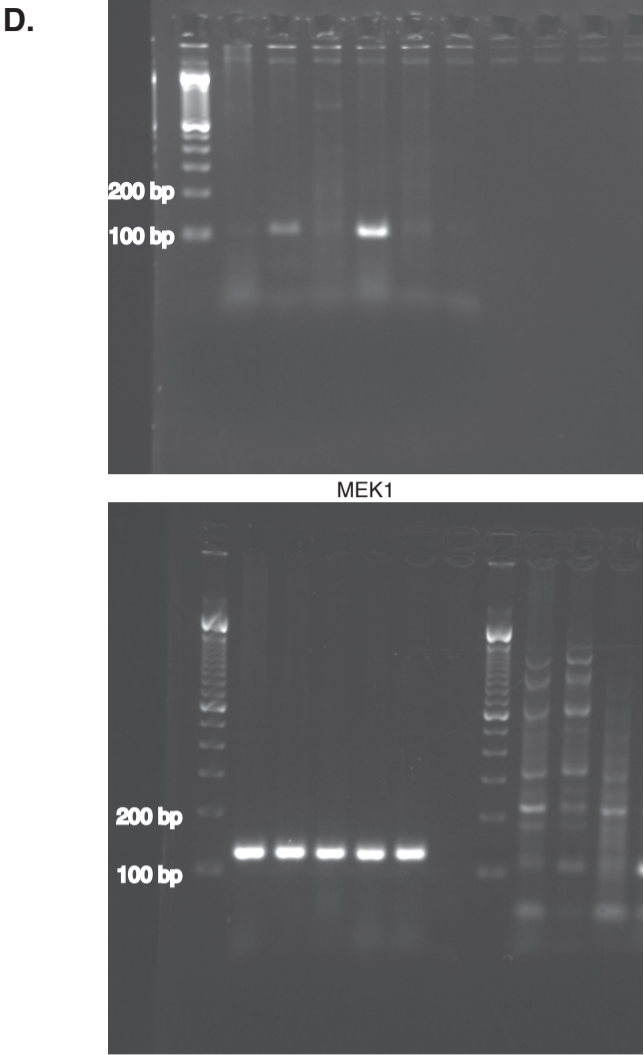

Supplement: Supplementary file 7 [file emmm0007-1104-sd7.pdf]
